# Non-Covalent Binding of Tripeptides-Containing Tryptophan to Polynucleotides and Photochemical Deamination of Modified Tyrosine to Quinone Methide Leading to Covalent Attachment

**DOI:** 10.3390/molecules26144315

**Published:** 2021-07-16

**Authors:** Antonija Erben, Igor Sviben, Branka Mihaljević, Ivo Piantanida, Nikola Basarić

**Affiliations:** 1 Department of Organic Chemistry and Biochemistry, Ruđer Bošković Institute, Bijenička cesta 54, 10 000 Zagreb, Croatia; antonija.erben@irb.hr (A.E.); igor.sviben@irb.hr (I.S.); ivo.piantanida@irb.hr (I.P.); 2 Department of Material Chemistry, Ruđer Bošković Institute, Bijenička cesta 54, 10 000 Zagreb, Croatia; branka.mihaljevic@irb.hr

**Keywords:** DNA, photodeamination, quinone methide, RNA, tripeptide, tryptophane

## Abstract

A series of tripeptides TrpTrpPhe (**1**), TrpTrpTyr (**2**), and TrpTrpTyr[CH_2_N(CH_3_)_2_] (**3**) were synthesized, and their photophysical properties and non-covalent binding to polynucleotides were investigated. Fluorescent Trp residues (quantum yield in aqueous solvent Φ_F_ = 0.03–0.06), allowed for the fluorometric study of non-covalent binding to DNA and RNA. Moreover, high and similar affinities of **2×HCl** and **3×HCl** to all studied double stranded (ds)-polynucleotides were found (log*K*_a_ = 6.0–6.8). However, the fluorescence spectral responses were strongly dependent on base pair composition: the GC-containing polynucleotides efficiently quenched Trp emission, at variance to AT- or AU-polynucleotides, which induced bisignate response. Namely, addition of AT(U) polynucleotides at excess over studied peptide induced the quenching (attributed to aggregation in the grooves of polynucleotides), whereas at excess of DNA/RNA over peptide the fluorescence increase of Trp was observed. The thermal denaturation and circular dichroism (CD) experiments supported peptides binding within the grooves of polynucleotides. The photogenerated quinone methide (QM) reacts with nucleophiles giving adducts, as demonstrated by the photomethanolysis (quantum yield Φ_R_ = 0.11–0.13). Furthermore, we have demonstrated photoalkylation of AT oligonucleotides by QM, at variance to previous reports describing the highest reactivity of QMs with the GC reach regions of polynucleotides. Our investigations show a proof of principle that QM precursor can be imbedded into a peptide and used as a photochemical switch to enable alkylation of polynucleotides, enabling further applications in chemistry and biology.

## 1. Introduction

Non-covalent binding of peptides/proteins and DNA or RNA is an important biological process enabling transcription of DNA and cell replication [1,2]. Understanding the recognition process between polynucleotides and peptides and being able to affect these events [3] provide immense scientific advantage, enabling numerous applications in biochemistry, biology, and medicine. Therefore, binding of peptides to polynucleotides has been intensively investigated [4,5]. Examples of DNA-binding peptides peptides/proteins are numerous, for instance zinc finger [6], or leucine zipper motives [7], as well as many other, even small peptides [4]. Furthermore, a special attention was devoted to synthetic peptides that selectively recognize DNA [8,9] or DNA-binding oligopeptides which can be modulated by light [10]. Peptide-binding units can easily be functionalized by fluorophores [11,12], providing facile methods for DNA fluorescent labeling and visualization [13].

Schmuck et al. designed a structurally modified peptide with incorporated aminonaphthalimide as a fluorophore, which binds to nucleoside triphosphates [14]. Furthermore, Schmuck et al. used peptides rich in lysine for the binding to polynucleotides, whereas fluorescence response was enabled by tryptophan and pyrene [15] or fluorescence resonance energy transfer (FRET) between naphthalene and dansyl [16]. Piantanida et al. have recently explored the binding of a series of structurally modified fluorophore-peptides to polynucleotides [17,18,19,20]. The polynucleotide-binding units in these molecules were phenanthridines [19], pyrenes [20], or guanidinocarbonylpyrroles [17,18] connected to the peptide scaffolds. The binding was characterized by generally high association constants and significant changes in fluorescence intensity. However, canonic amino acids can also be involved in the polynucleotide recognition, as it is the case in nature. For example, binding to DNA was demonstrated for tripeptides containing bis-tryptophan units [21,22]. Owing to tryptophan fluorescence, the binding can also be monitored by fluorescence spectroscopy.

Quinone methides (QMs) are reactive intermediates in the chemistry and photochemistry of phenols [23], that have received scientific attention due to applications in synthesis [24,25] and biological activity [26,27]. Particularly appealing for the application in biological systems is the fact that QMs can be generated in photochemical reactions under mild conditions [28,29]. The biological effects of QMs were connected to their reactivity with proteins [30,31,32], nucleobases [33], DNA [34,35,36,37], and G-quadruplexes [38,39,40,41]. Reversible DNA cross-linking by QMs [42,43,44] and intracellular formation of QMs followed by DNA cross-linking is believed to lead to the antiproliferative action of the anticancer antibiotic mitomycine [45,46,47]. However, for the efficient DNA alkylation and cross-linking, it is important to attach to the QM precursor units to groups that can bind to DNA by non-covalent interactions [35]. In that way, the QM precursor is positioned in the proximity to DNA making the reactivity with DNA more likely in competition with hydrolysis [48,49,50,51].

**Figure 1:**
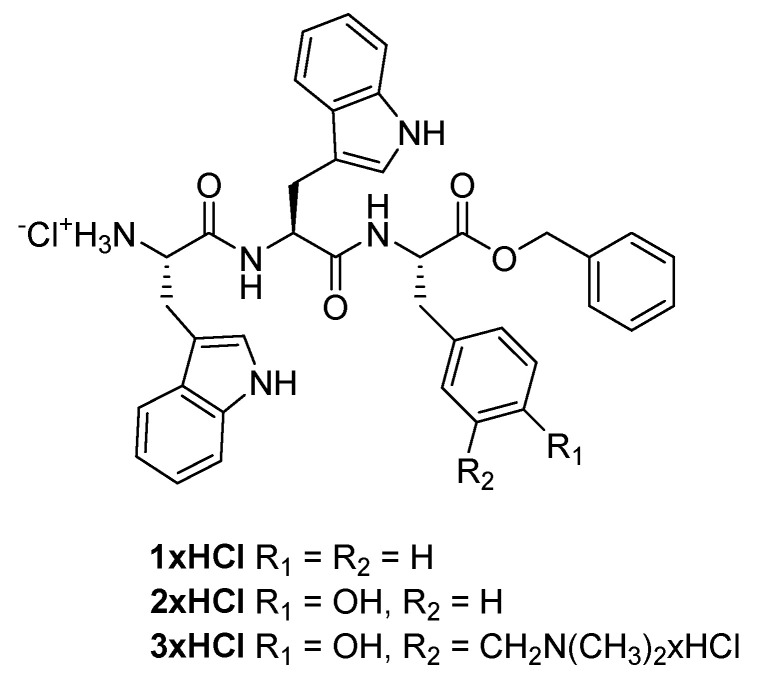
Investigated tripeptides **1×HCl**–**3×HCl**.

These findings prompted us to investigate the non-covalent binding to polynucleotides for a series of tripeptides **1×HCl**–**3×HCl** (Figure 1) containing bis-tryptophan units, wherein the C-terminus contained Phe, Tyr, or modified Tyr, which is photochemically reactive [52]. Upon excitation by light, the modified tyrosine undergoes photodeamination delivering QM [53,54], and if such peptide is non-covalently bound to DNA or RNA, that would allow QM to react with DNA/RNA and induce permanent covalent damage. Herein we demonstrate that tripeptides **1**–**3** bind to DNA by non-covalent interactions. The additional positive charge in the photoreactive group in **3** increases its solubility in H_2_O, and enhances its binding ability with DNA. Furthermore, by employing preparative irradiations we show that **3** undergoes photodeamination and delivers QMs. Laser flash photolysis (LFP) was conducted to detect the reactive intermediates in photochemistry of **3**, whereas fluorescence spectroscopy was utilized to characterize photophysical properties of all tripeptides, which is important for the study of binding to polynucleotides. Although photogenerated QM from **3** does not react with nucleotides, photoinduced alkylation of polynucleotides by **3** is feasible, as demonstrated by the reaction of double-stranded oligonucleotides. Therefore, incorporation of the photoswitchable unit in peptides increases the ability for DNA binding and can be used in photoinduced attachment of fluorophores in DNA labeling.

## 2. Results and Discussion

### 2.1. Synthesis

**Scheme 1:**
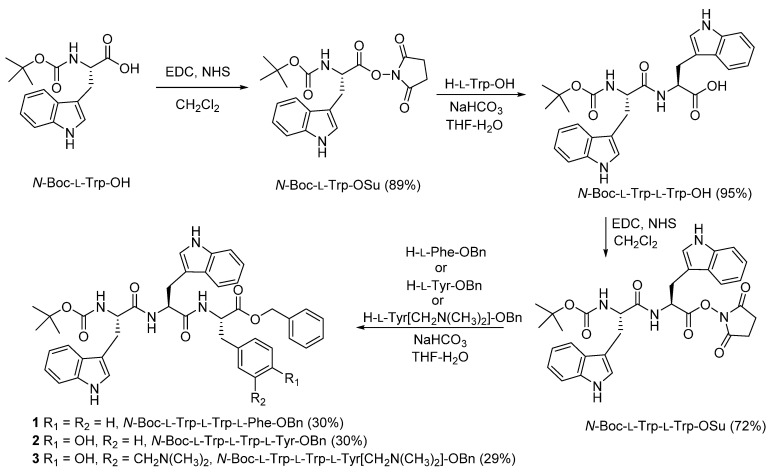
Synthesis of **1**–**3**.

The synthetic protocol for the preparation of tripeptides **1**–**3** was based on the standard Boc-chemistry in solution where the activation of carboxylic acids was achieved by the use of *N*-hydroxysuccinimide (NHS) and 1-ethyl-3-(3-dimethylaminopropyl)carbodiimide (EDC) [55]. In the first step, *N*-Boc-L-Trp-OH was activated by EDC and transformed to the succinimide ester *N*-Boc-L-Trp-OSu, which was isolated (Scheme 1). The following coupling with H-L-Trp-OH afforded dipeptide *N*-Boc-L-Trp-L-Trp-OH in excellent yield. The free carboxylic functional group of the dipeptide was activated again by transforming it to a succinimide ester, which was coupled with Phe, Tyr, or modified Tyr, prepared according to the literature precedent [52]. The coupling afforded desired peptides **1**–**3** in moderate yields. For the study with polynucleotides removal of the Boc was facilitated by the treatment with HCl dissolved in dry EtOAc, whereupon HCl salts were obtained.

### 2.2. Photophysical Properties

**Figure 2:**
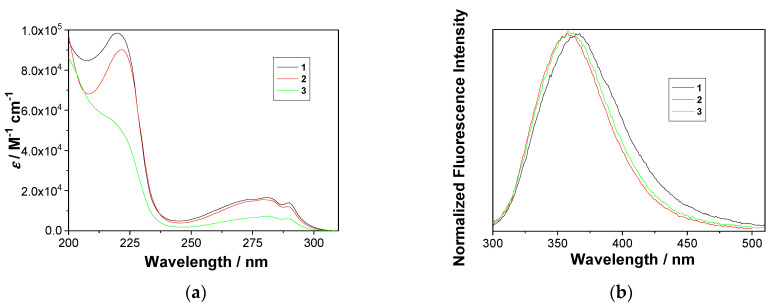
Absorption (**a**) and normalized emission spectra (**b**, *λ*_ex_ = 280 nm) of **1**–**3** in CH_3_CN. Emission properties of **1**–**3** are dominated by Trp fluorescence, whereas the third amino acid has minor effect on the absorption spectra only.

Photophysical properties of **1**–**3**, and the corresponding HCl salts, are important for the study of their binding to polynucleotides, as well as for deeper understanding of the photochemical reactivity of **3**. Absorption and fluorescence spectra for **1**–**3** were measured in CH_3_CN (Figures S1–S8 in the SI), whereas assays with biomacromolecules for the corresponding salts were conducted in aqueous cacodylate buffer (pH = 7, 50 mM), to which some DMSO was added to assure solubility. Absorption spectra of **1**–**3** are shown in Figure 2. In the low energy region, the absorption band with a maximum at 280 nm and a shoulder at 290 nm dominate for all tripeptides, the typical for tryptophan. The third amino acid, Phe, Tyr, or Tyr[CH_2_N(CH_3_)_2_], has only a minor influence on the absorption spectra, with **1** and **2** showing higher absorptivity than H-Trp-Trp-OH, and **3** lower (Figures S2, S5 and S7 in the SI). The finding suggests that there is some electronic interaction between the chromophores in the ground state. Due to large aromatic residues, it is plausible that tripeptides **1**–**3** form α-helix where the first and the third amino acid are positioned in the proximity to interact, leading to the hyperchromic effect with **1** and **2**, and hypochromic effect with **3**.

Fluorescence spectra of **1**–**3** in CH_3_CN are characterized by one band with a maximum at 358 nm for **2** and **3**, and 366 nm for **1**, the typical for Trp. The position of the maxima and the shape of the spectra do not depend on the excitation wavelength, indicating that the emission originates from Trp only. However, fluorescence spectra of **1** in cacodylate buffer at higher concentration (2 mM) exhibit bathochromic shifts of 10 nm (Figure S3 in the SI), suggesting aggregation of the molecules. Such an aggregation was not observed for **2** and **3**. Fluorescence spectra of **3** exhibit solvatochromic properties (Figure S8, left, in the SI), which are in accordance with reports on Trp photophysics [56]. Thus, **3** in CH_3_CN and **3×HCl** in CH_3_CN-H_2_O (1:9), show difference of 23 nm between the maxima of emission spectra, which is due to different spectral properties of Trp derivatives in solvents of different polarity and proticity [57]. Understanding spectral and photophysical properties, which are dependent on solvent polarity and proticity, is important for the study of non-covalent binding of these peptides to macrobiomolecules including polynucleotides.

Quantum yields of fluorescence (Φ_f_) were measured for **1**–**3** in CH_3_CN, and for the corresponding salts Φ_f_ was measured in aqueous solution CH_3_CN-H_2_O (1:9), containing sodium cacodylate buffer (pH = 7.0, 50 mM), (Table 1). *N*-acetyltryptophanamide (NATA) in H_2_O was used as a reference (Φ_f_ = 0.14) [58]. Note that Φ_f_ for multichromophoric systems **1**–**3** depends on the excitation wavelength, and we report the average value. However, for aqueous and non-aqueous solution, Φ_f_ is similar for **1** and **2**, and about double than Φ_f_ for **3**, presumably due to photochemical deactivation delivering QMs from **3**.

Decays of fluorescence for **1**–**3** in CH_3_CN, as well as for **2×HCl** and **3×HCl** in CH_3_CN-H_2_O (1:9) were measured by time-correlated single-photon counting (TC-SPC) upon excitation at 280 nm (Table 1 and Figures S9 and S10 in the SI). None of the fluorescence decays could be fit to single exponential function, which is anticipated for multichromophoric systems. Furthermore, it is known that Trp derivatives often do not exhibit single exponential decay due to the presence of two excited states, L_a_ and L_b_ [59].

### 2.3. Photochemistry

**Scheme 2:**
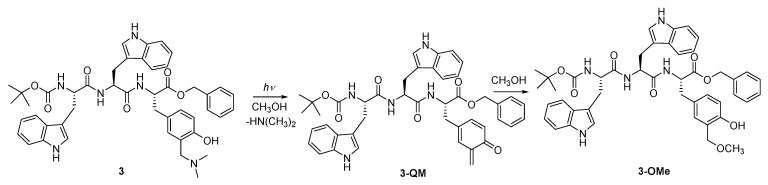
Photomethanolysis of **3**.

Photodeamination of aminomethylphenoles in aqueous CH_3_OH leads to photomethanolysis products via QM intermediates [52,53]. To demonstrate that photometahnolysis of tripeptide **3** also takes place, we performed preparative irradiation of the fully protected compound (bearing Boc and Bn) to make the photoproduct isolation easier. The irradiations were conducted in CH_3_OH solutions at 300 nm and the composition of the solutions was analyzed by HPLC. Methanolysis of **3** run to the conversion of 65% gave methyl ether **3-OMe**, which was isolated in 22% yield (Scheme 2). In addition, photomethanolysis of salt **3×HCl** was conducted, but it was analyzed by HPLC only. It gave cleanly only one photoproduct, presumably the photomethanolysis ether.

Efficiency of the photomethanolysis reaction (Φ_R_) was measured by the use of a primary actinometer, KI/KIO_3_ (Φ_254_ = 0.74) [58,60], and the samples were excited at 254 nm. The efficiency was measured for **3** and **3×HCl**, providing similar values of Φ_R_ = 0.13 ± 0.01 and Φ_R_ = 0.11 ± 0.02, respectively. Relative efficient formation of the photomethanolysis product is highly indicative that deamination takes place via a QM intermediate, as reported in the literature precedent [52,53].

### 2.4. Laser Flash Photolysis (LFP)

To detect QM intermediates and other plausible intermediates in the photochemistry of tripeptide **3**, LFP was used. The samples were excited with a Nd:YAG laser at 266 nm. The measurements were performed in N_2_ and O_2_-purged CH_3_CN solution, where O_2_ was expected to quench triplets and radicals, but not QMs. Moreover, the spectra and decay kinetics were measured in CH_3_CN and CH_3_CN-H_2_O (1:1) where the difference was expected due to excited state proton transfer (ESPT) pathways, possible in aqueous solvent only [61,62,63,64] (for all transient absorption spectra and the associated decay kinetics see Figures S11–S37).

In Ar-purged CH_3_CN solution of **3** we detected two transients absorbing over the whole spectrum with a maximum at ≈350 nm. The lifetimes of the transients were 200 ± 50 ns, probably corresponding to the triplet excited state or some radical (assignment based on the quenching by O_2_), and 450 ± 100 μs tentatively assigned to **QM-3** since in the O_2_-purged solution it has a similar lifetime, ≈400 μs. The spectra show additional transient absorbing at ≈350 nm and ≈550 nm that is not quenched by O_2_. It may correspond to species generated from Trp, such as indolyl radical-cation and *N*-radical [65,66,67]. In the aqueous Ar-purged solution of **3** two transients were detected with the lifetimes of 10–30 ns, probably corresponding to the triplet excited state or some radical and the one with the lifetime 190 ± 10 μs, tentatively assigned to the QM since it is not quenched by O_2_. In O_2_-purged solution the longest-lived transient has a similar lifetime, ≈150–200 μs.

In the LFP experiments for the salt **3×HCl**, in Ar-purged solution we detected three transients with the lifetimes of ≈10 ns, probably corresponding to the triplet excited state or some radical, 10.6 ± 0.6 μs, not assigned and 1.3 ± 0.6 ms, tentatively assigned to the QM. In O_2_-purged solution we detected a similar long-lived transient with the lifetime, ≈0.5–10 ms whose precise determination of decay kinetics was difficult due to very low intensity and long decay time.

**Figure 3:**
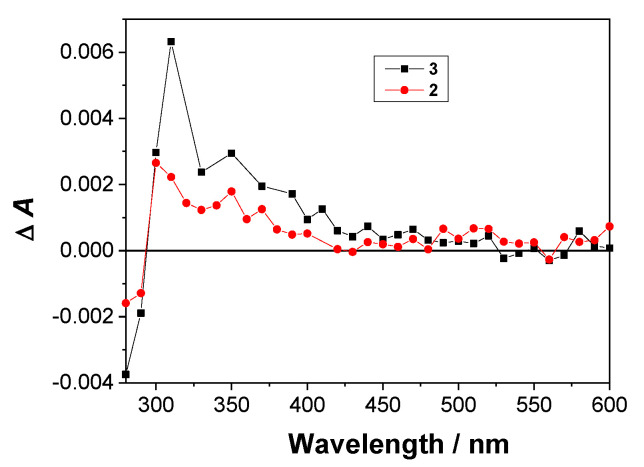
Transient absorption spectra of optically matched (*A*_266_ = 0.30) O_2_-purged solution of **3** in CH_3_CN (*c* = 6.1 × 10^−5^ M) and **2** in CH_3_CN (*c* = 2.95 × 10^−5^ M). The spectra were measured 27 μs after the laser pulse at 266 nm, laser power ≈19 mJ/pulse. The difference in the transient absorption spectra of photochemically nonreactive compound **2** and reactive **3** were assigned to the presence of QM.

Since the very low intensity of the transient (Δ*A* < 10^−3^) that was tentatively assigned to QM-precluded quenching experiments, we performed control LFP experiments for **2**, where the formation of QM is not possible. Figure 3 shows transient absorption spectra of **2** and **3** recorded after a delay of 27 μs after the laser pulse, revealing some difference at 300–400 nm, which may be assigned to the presence of QM based on the position of the anticipated maximum in the absorption spectra [52,53]. This long-lived transient with the lifetime of ≈400 μs was seen from **3** (but not from **2**), we therefore assigned to **QM-3**. Interestingly, in the aqueous solution, a difference between the transient absorption spectra between **2** and **3** was not observed, presumably due to pronounced formation of *N*-Trp radicals from both peptides, in the process that involves photoionization to radical cation and its deprotonation [65,66,67].

### 2.5. Non-Covalent Binding to Polynucleotides

#### 2.5.1. Fluorescence Titrations

The moderate fluorescence of the studied **1×HCl**–**3×HCl** allowed fluorescence titrations with various double stranded (ds)-DNA/RNA, whereby we used for excitation *λ*_exc_ = 295 nm (selective for tryptophan) to avoid absorbing the light by increasing the amounts of DNA/RNA (for all data see Figures S38–S54 in the SI).

**Figure 4:**
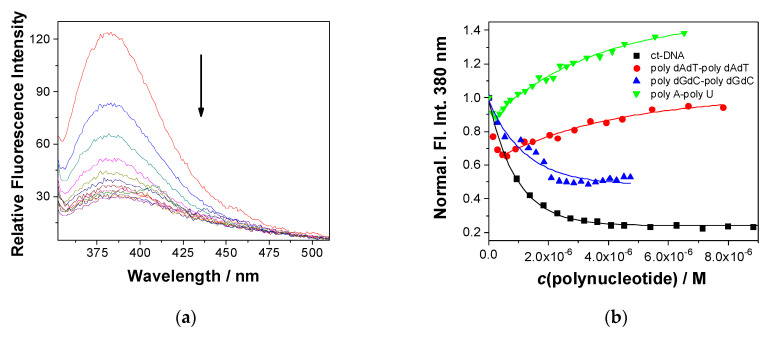
(**a**): Fluorescence titration of **3×HCl** (*c* = 3.0 × 10^−6^ M, *λ*_exc_ = 295 nm) with ct-DNA; (**b**): dependence of the fluorescence intensity at *λ*_em_ = 380 nm (normalized to starting emission) on the *c*(polynucleotide); dots are experimental values and the lines represent calculated fit to the Scatchard model [68], with the fixed value of *n* = [bound **3×HCl**]/[polynucleotide] = 0.3. The measurements were performed in cacodylate buffer (pH 7.0, 50 mM, at 25 °C). Depending on the polynucleotide, different fluorescence responses were obtained which were used to estimate the binding constants of the complexes with polynucleotides.

For **1×HCl**, 20% DMSO had to be added to the solution to increase the solubility of the peptide in H_2_O and for that reason only preliminary titration with *calf thymus*-DNA (ct-DNA) was performed (Figure S38). For better solubility **2×HCl** and **3×HCl** titrations were performed with all DNA/RNA, resulting in quite different emission response. Namely, addition of guanine-containing DNAs (ct-DNA and GC-DNA) exclusively quenched emission of both, **2×HCl** (Figure S39) and **3×HCl** (Figure 4). At variance, addition of AT-DNA caused either very small changes (**2×HCl**, Figure S40) or bisignate changes: (a) quenching of emission of **3×HCl** (Figure 4) at excess of dye (*r*[dye]/[DNA] > 0.5) followed by (b) emission increase at an excess of DNA (*r* < 0.5). The addition of AU-RNA yielded also biphasic changes with much more pronounced emission increase (**3×HCl** Figure 3 and **2×HCl** Figure S42).

Further, we processed the titration data collected at excess of DNA/RNA-binding sites over dye (*r*[dye]/[DNA] > 0.25) by nonlinear regression analysis according to the Scatchard model (McGhee, von Hippel formalism) [68], to calculate the binding constants (Table 2). The affinities of **2×HCl**, **3×HCl** to all studied ds-polynucleotides were similar (log*K*_a_ = 6.0–6.8), thus suggesting that the observed difference in emission response (Figure 4b, Table 2) is not a result of the binding strength, but more likely the result of fine differences in positioning of the fluorophore within the DNA/RNA-binding site, affecting the Trp photophysical properties.

Comparison of fluorescence responses of synthetic DNA/RNAs can be correlated to the corresponding secondary structures. Namely, AT-DNA minor groove size and shape is excellently suited for small molecule binding [69,70], allowing also the insertion of dimeric species at excess of dye over DNA-binding sites (*r*[dye]/[DNA] > 0.5) [71], which can at excess of DNA (*r* < 0.25) de-aggregate and each molecule binds to individual binding site. At contrast to AT-DNA, GC-DNA is characterized by guanine amino groups protruding into minor groove, which sterically allows insertion of only single molecules. Moreover, guanine is the nucleobase with the lowest oxidation potential [72], allowing for photoinduced electron transfer (PET) between the peptide and guanine and leading exclusively to the fluorescence quenching. PET has been also documented for some 4,9-pyrene and acridine derivatives, which showed guanine-induced quenching of emission [73]. Finally, AU-RNA is well-known for narrow and deep, exceedingly hydrophobic major groove, which can bind small molecules, and due to efficient H_2_O exclusion yield strong emission increase of hydration-sensitive fluorophores (like Trp in this case).

#### 2.5.2. Thermal Denaturation

The thermal denaturation experiments provide information about the ds- polynucleotide helix thermal stability as a function of interaction with added small molecules [74]. The difference between the *T*_m_ value of free ds-polynucleotide and a complex with a small molecule (Δ*T*_m_ value) is an important factor in the characterization of small molecule/ds-polynucleotide interactions. For instance, moderate to strong stabilization (Δ*T*_m_ > 5 °C) supports pronounced intercalative or minor groove-binding interaction [70], whereas weak or negligible stabilization (Δ*T*_m_ = 0–5 °C) suggests a binding process driven mostly by hydrophobic effect accompanied by weak H-bonding and/or electrostatic interactions—usually excluding classical intercalation as a binding mode.

Since only **3×HCl** showed measurable interaction with ds-DNA/RNA in fluorescence titrations, we investigated the impact of **3×HCl** on thermal denaturation of ct-DNA. The compound had only negligible effect to the stability of double helix (Figure S49 and Table S3 in the SI), increasing the melting temperature (*T*_m_) by 0.5 °C only, which is within the experimental error. However, upon irradiation (300 nm, 5 min) of ct-DNA in the presence of the compound, the thermal stability of ds-DNA decreased (Δ*T_m_* = −2.2 °C), indicating destabilization of double helix, which may be due to photochemical alkylation of a single nucleobase, consequently resulting in steric hindrance of base pair recognition and also adjacent base pair stacking and thus decreasing the thermal stability of polynucleotide double helical structure.

#### 2.5.3. CD Experiments

To investigate the mode of binding of peptides **1×HCl**–**3×HCl** to ct-DNA, circular dichroism (CD) spectroscopy was used. CD spectroscopy is an useful analytical tool in the binding study of small molecules to chiral macromolecules such as DNA [75] since it can provide information on the binding mode to polynucleotide, with distinctive spectral differences for intercalators and groove-binding derivatives [76,77]. Compounds are chiral and possess CD spectra, and measurable positive signals are located around 235 nm, thus not interfering with the positive CD bands of DNA/RNA in the 260–290 nm range.

**Figure 5:**
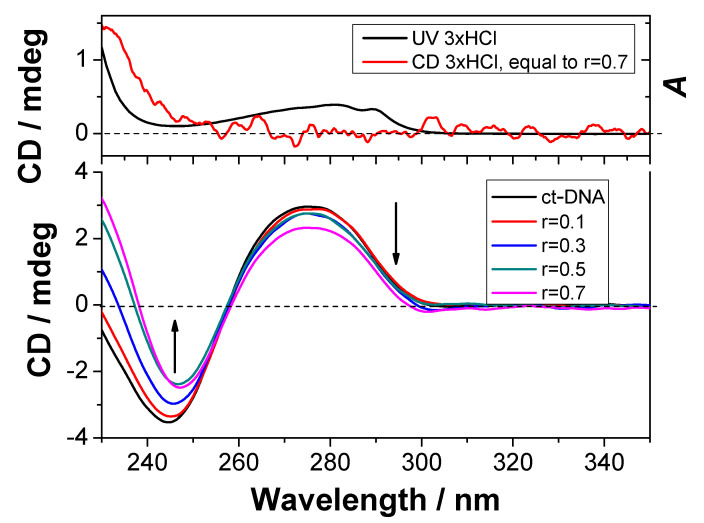
UP: CD and UV spectrum of **3×HCl**, *c* = 2.0 × 10^−5^ M. DOWN: CD spectra of ct-DNA (*c* = 3.0 × 10^−5^ M) in the presence of different ratios *r*[**3 ×HCl**]/[ct-DNA]. The measurements were performed in cacodylate buffer (pH 7.0, 50 mM, at 25 °C). A small decrease of the CD signal of DNA at 275 nm upon addition of **3×HCl** suggests a minor loss of DNA helicity due to the binding.

Thus, the small decrease of ct-DNA positive band at 275 nm caused by the addition of **3×HCl** (Figure 5) can be attributed to binding of small molecule to DNA and consequent unwinding of DNA double helix to accommodate small molecule, resulting in minor loss of helicity (noted as CD band decrease). Changes in 230–260 nm range are mostly due to the additive impact of CD of **3×HCl** (Figure 5 UP). Analogously, upon addition of **3×HCl** to GC-DNA and AU-RNA minor decrease of positive bands at 260–290 nm range was observed (Figures S53 and S55 in the SI), while for AT-DNA the change was negligible (Figure S52 in the SI). Similar trend was observed in the CD spectra of ct-DNA with **1×HCl** (with 20% DMSO) and **2×HCl** (Figures S50 and S51 in the SI).

In summary, CD and fluorescence titration results, as well as thermal denaturation experiments, support binding of **1**–**3** into minor groove of DNAs and major groove of RNA. Sterically more restricted GC-DNA groove allows only binding of single molecules, at variance to AT-DNA minor and AU-RNA major groove, which also allow binding of dimers (only at excess of peptide over DNA/RNA-binding sites).

### 2.6. Covalent Binding to Polynucleotides

Covalent binding of tripeptide **3** (1 mM) to nucleotides was essayed by irradiation (300 nm) in the presence of the following nucleotides (10 mM): 2′-deoxyadenosine, 2′-deoxycytidine, 2′-deoxyguanidine, 2′-deoxyadenosine-5′-diphosphate, 2′-deoxycytidine-5′-monophosphate, and 2′-deoxyguanidine-5′-monophosphate. However, HPLC-MS and NMR analyses revealed no trace of any adduct to nucleotide. The finding is in accord with the Rokita’s results that non-covalent binding of the QM precursors was essential for the alkylation to occur [35]. The affinity of peptides toward mononucleotides was too low to ensure close contact in the moment of QM generation. Therefore, we investigated aptitude of photogenerated QM from **3** to alkylate double stranded DNA strains.

Models for double stranded (ds) DNA were obtained by annealing complementary oligonucleotides (dG_12_ with dC_12_, or dA_10_ with dT_10_). After mixing oligonucleotides in the appropriate concentrations their solutions were heated to 90 °C and allowed to cool slowly to rt to initiate the annealing. Formation of the ds-dG_12_-dC_12_ and dA_10_-dT_10_ was demonstrated by the thermal denaturation experiment (Figure S55 in the SI) and CD spectroscopy (Figure S56 in the SI). The solutions containing the annealed ds-oligonucleotides in ammonium acetate buffer were analyzed by HPLC (for details see Table S4 in the SI and Figures S57–S75), where dG_12_-dC_12_ shows up as one peak with the retention time of 7 min (Figure S57) and dA_10_-dT_10_ as two signals with the retention times of 7 min and 19 min, corresponding to the non-protonated and protonated form, respectively.

The addition of **3×HCl** to the solutions of ds-oligonucleotides affected their retention times on HPLC due to the non-covalent binding and formation of complexes. Thus, in the presence of **3×HCl** the retention time of dG_12_-dC_12_ was retarded by ≈0.3 min (Figure S60), whereas the effect was opposite for the dA_10_-dT_10_ where the signal at 7 min appeared ≈0.5 min earlier (Figure S72). Further, we performed irradiations of the mixtures of **3×HCl** with ds-oligonucleotides (300 nm, 15 or 60 min), and the composition of the irradiated solutions was analyzed by HPLC. As control experiments we performed irradiation of **3×HCl** under the same conditions in the same buffered solutions, as well as irradiations of ds-oligonucleotides. Interestingly, overlapped HPLC chromatograms after the irradiation **3×HCl** and dG_12_-dC_12_ do not show any new signal which could be attributed to the photoinduced alkylation of the oligonucleotides (Figures S61–S64), whereas upon the irradiation of **3×HCl** and dA_10_-dT_10_ a new broad signal at ≈16.5 min appeared, not seen in the control irradiation experiments (Figures S71, S73 and S74). This finding is at variance to previous reports on alkylation of polynucleotides by *o*-QMs, where the reaction at the exocyclic guanine NH_2_ group predominated [33]. The observed preferable alkylation of AT may be explained by specific binding of **3×HCl** to AT-sequence, probably as an aggregate inserted deeply within the minor groove, whereupon the adenine residues are the site of attack by QMs. On the contrary, binding of single molecules **3×HCl** to the minor groove of GC-sequence sterically blocked by protruding amino groups left the peptide much more exposed to the aqueous environment, which upon exposure to irradiation and formation of QMs lead primarily to hydrolysis [48,49], instead of the alkylation. The results further indicate that positioning of the QM-precursor in the proximity of the ds-helices probably plays more important role than the intrinsic reactivity of the QM.

## 3. Materials and Methods

### 3.1. General

^1^H and ^13^C NMR spectra were recorded at 300 or 600 MHz at rt, using TMS as a reference and chemical shifts were reported in ppm. Melting points were determined using a Mikroheiztisch apparatus and were not corrected. IR spectra were recorded on a spectrophotometer in KBr and the characteristic peak values were given in cm^−1^. HRMS were obtained on a MALDI TOF/TOF instrument. Semipreparative HPLC separations were performed on a Varian Pro Star instrument equipped with a Phenomenex Jupiter C18 5μ 300A column, using CH_3_OH/H_2_O + TFA as an eluent. Analysis of samples was performed on a Shimadzu HPLC equipped with a diode-array detector, and a Phenomenex Luna 3u C18(2) column was used. Mobile phase was CH_3_OH/H_2_O + TFA. Analyses of the solutions containing annealed oligonucleotides and **3×HCl** were performed on a HPLC equipped with a PLRP-S 5 µm column and the mobile phase was CH_3_CN/ammonium acetate aqueous buffer. Details on methods for the sample analysis are given in the SI. Irradiation experiments were performed in a reactor equipped with 8 lamps with the output at 254 or 300 nm (1 lamp 8 W). Solvents for the irradiations were of HPLC purity. Chemicals were purchased from the usual commercial sources and were used as received. Solvents for chromatographic separations were used as they are delivered from supplier (p.a. or HPLC grade) or purified by distillation (CH_2_Cl_2_). Preparation of known compounds, *N*-Boc-L-Trp-OSu [78], *N-*Boc-L-Trp-L-Trp-OH [21], *N-*Boc-L-Trp-L-Trp-OSu [78], *N*-Boc-L-Trp-L-Trp-L-Phe-OBn [21], *N-*Boc-L-Trp-L-Trp-L-Tyr-OBn [21] are given in the supporting information. Polynucleotides were purchased as noted: poly A-poly U, poly (dGdC)_2_, poly dG-poly dC, poly (dAdT)_2_, ct-DNA (Sigma, St. Louis, MO, USA). Polynucleotides were dissolved in Na-cacodylate buffer, *I* = 0.05 M, pH 7.0. ct-DNA was additionally sonicated and filtered through a 0.45-mm filter. Polynucleotide concentration was determined spectroscopically as the concentration of phosphates (nucleobases), as described by producer.

### 3.2. Preparation of N-Boc-L-Trp-L-Trp-L-Tyr[CH_2_N(CH_3_)_2_]-OBn *(**3**)*

A solution of *N*-Boc-L-Trp-L-Trp-OSu (320 mg, 0.65 mmol) in THF-u (6 mL) was added slowly to a suspension of the salt TFA×H-Tyr[CH_2_N(CH_3_)_2_]-OBn (315 mg, 0.6 mmol) and NaHCO_3_ (252 mg, 3.0 mmol) in a mixture of THF and H_2_O (1:1, 12 mL). The reaction mixture was stirred at rt over 3 days. THF was removed on a rotary evaporator and the aqueous residue was acidified by HCl (0.5 M) to the pH value of 2–3. The extraction with EtOAc (3 × 50 mL) was carried out and the extracts were dried over anhydrous sodium sulfate. After filtration, the solvent was removed on a rotary evaporator and the crude residue was purified on a column of silica gel using CH_3_OH/CH_2_Cl_2_ (1→20%) as eluent to afford the pure oily product (0.14 g, 29%).

^1^H NMR (CD_3_OD, 600 MHz) *δ*/ppm: 7.52 (d, *J =* 7.5 Hz, 1H), 7.36–7.29 (m, 5H), 7.29–7.25 (m, 3H), 7.11–7.06 (m, 2H), 7.02–6.98 (m, 1H), 6.94–6.89 (m, 4H), 6.86 (d, *J =* 7.5 Hz, 1H), 6.68 (d, *J =* 8.0 Hz, 1H), 5.06 (s, 2H), 4.58–4.49 (m, 2H), 4.23–4.18 (m, 1H), 3.90 (s, 2H), 3.10–3.03 (m, 3H), 2.95 (dd, *J =* 6.4, 14.0 Hz, 1H), 2.90 (dd, *J =* 6.4, 14.7 Hz, 1H), 2.81 (dd, *J =* 6.7, 14.0 Hz, 1H), 2.50 (s, 6H), 1.27 (s, 9H); ^13^C NMR (CD_3_OD, 75 MHz) *δ*/ppm: 177.0 (s, 1C), 173.5 (s, 1C), 172.0 (s, 1C), 156.9 (s, 1C), 138.1 (s, 1C), 138.0 (s, 1C), 137.0 (s, 1C), 133.5 (d, 1C), 132.8 (d, 1C), 129.60 (d, 2C), 129.58 (d, 2C), 129.4 (d, 1C), 128.8 (s, 2C), 128.7 (d, 1C), 124.9 (d, 1C), 124.8 (d, 1C), 122.56 (d, 1C), 122.54 (d, 1C), 119.96 (d, 1C), 119.95 (d, 1C), 119.4 (d, 1C), 119.2 (d, 1C), 116.5 (d, 1C), 112.4 (d, 2C), 110.6 (s, 1C), 110.2 (s, 1C), 81.0 (s, 1C), 68.0 (t, 1C), 59.1 (t, 1C), 57.5 (d, 1C), 55.6 (d, 1C), 55.2 (d, 1C), 43.6 (q, 2C), 37.2 (t, 1C), 28.7 (t, 1C), 28.5 (q, 3C), 28.2 (t, 1C), two singlets were not seen; HRMS (MALDI-TOF) *m*/*z* [M + H]^+^ calculated for C_46_H_52_N_6_O_7_ 801.3976; found 801.3989.

### 3.3. Preparation of HCl×H-L-Trp-L-Trp-L-Tyr[CH_2_N(CH_3_)_2_]-OBn *(**3×HCl**)*

***N-*Boc-L-Trp-L-Trp-L-Tyr[CH_2_N**(**CH_3_**)**_2_]-OBn** (**3**, 23 mg, 0.03 mmol) was dissolved in anhydrous EtOAc to which a saturated solution of HCl in EtOAc was added (5 M, 1 mL). The reaction mixture was stirred 1 h at rt and the solvent was removed in vacuum. The remaining crystals were washed with Et_2_O and dried on a rotary evaporator. The product was purified by preparative TLC on silica gel using 20% MeOH/CH_2_Cl_2_ as eluent to afford the oily product (14 mg, 66%). Alternatively, the product was purified by semipreparative HPLC.

^1^H NMR (CD_3_OD, 600 MHz) *δ*/ppm: 7.57 (d, *J* = 8.0 Hz, 1H), 7.38–7.21 (m, 9H), 7.09 (dd, *J =* 7.0, 14.0 Hz, 2H), 7.05–6.97 (m, 2H), 6.91 (d, *J* = 7.0 Hz, 1H), 6.89–6.85 (m, 1H), 6.76 (dd, *J* = 1.8, 7.8 Hz, 1H), 6.68 (d, *J =* 1.8 Hz, 1H), 6.57 (d, *J* = 8.0 Hz, 1H), 5.05 (s, 2H), 4.66–4.55 (m, 2H), 3.59–3.49 (m, 3H), 3.07 (dd, *J* = 5.4, 14.6 Hz, 1H), 3.02 (dd, *J =* 7.0, 14.6 Hz, 1H), 2.96–2.76 (m, 4H), 2.28 (s, 6H); ^13^C NMR (CD_3_OD, 150 MHz) *δ*/ppm: 177.0, 173.6, 172.3, 157.5, 138.2, 138.0, 137.0, 131.5, 131.1, 129.6 (2C), 129.5 (2C), 129.4, 129.3, 128.3, 128.2, 124.9, 124.8, 122.6, 122.5, 122.4, 119.94, 119.86, 119.6, 119.4, 116.6, 112.42, 112.37, 111.0, 110.3, 68.0, 61.7, 56.4, 55.4, 55.0, 44.4, 37.6, 31.5, 28.7; HRMS (MALDI-TOF) *m*/*z* [M + H]^+^ calculated for C_41_H_44_N_6_O_5_ 701.3451; found 701.3445.

### 3.4. Irradiation of N-Boc-L-Trp-L-Trp-L-Tyr[CH_2_N(CH_3_)_2_]-OBn *(**3**)*

Four quartz test tubes were filled with a solution of **3** (4 × 10 mg, 4 × 0.013 mmol) in CH_3_OH (4 × 15 mL). The solutions were purged with N_2_ for 30 min, sealed and irradiated in a reactor equipped with 8 lamps with the output at 300 nm over 15 min. The composition of the irradiated solution was analyzed by HPLC. After the irradiation, the solvent was removed on a rotary evaporator and the residue was purified by TLC on silica gel using 10% CH_3_OH/CH_2_Cl_2_ as an eluent. The pure photoproduct was isolated in the form of oily substance (9 mg, 22%), and some starting compound was regenerated (8 mg, 20%).

^1^H NMR (CDCl_3_, 600 MHz) *δ*/ppm: 8.01 (br. s., 1H), 7.93 (br. s., 1H), 7.66 (d, *J =* 8.5 Hz, 1H), 7.41–7.30 (m, 7H), 7.27 (d, *J =* 8.2 Hz, 1H), 7.22 (dd (t), *J =* 7.5 Hz, 2H), 7.13 (dd (t), *J =* 7.5 Hz, 2H), 6.92–6.86 (m, 1H), 6.82 (br. s., 1H), 6.63 (d, *J =* 8.0 Hz, 1H), 6.58 (d, *J =* 8.0 Hz, 1H), 6.43 (br. s., 1H), 6.22–6.16 (m, 2H), 5.14 (d, *J =* 12.6 Hz, 1H), 5.08 (d, *J =* 12.6 Hz, 1H), 4.70 (dd, *J =* 6.6, 13.8 Hz, 1H), 4.59 (dd, *J =* 6.4, 12.3 Hz, 1H), 4.43–4.33 (m, 3H), 3.42 (s, 3H), 3.37–3.29 (m, 1H), 3.16–3.10 (m, 1H), 3.07 (dd, *J =* 7.7, 14.8 Hz, 1H), 2.88 (dd, *J =* 5.8, 14.1 Hz, 1H), 2.88–2.76 (m, 1H), 2.73–2.67 (m, 1H), 1.33 (s, 9H), all NH and OH signals were not seen; ^13^C NMR (CDCl_3_, 75 MHz) *δ*/ppm: 171.6 (s, 1C), 171.1 (s, 1C), 170.7 (s, 1C), 155.1 (s, 1C), 136.4(s, 1C), 136.1 (s, 1C), 130.4 (d, 1C), 129.3 (d, 1C), 128.80 (d, 2C), 128.77 (d, 2C), 128.7 (d, 1C), 127.4 (s, 1C), 127.2 (s, 1C), 123.5 (d, 1C), 123.4 (d, 1C), 122.6 (d, 1C), 122.3 (d, 1C), 122.2 (s, 1C), 120.0 (d, 1C), 119.8 (d, 1C), 119.3 (d, 1C), 118.7 (d, 1C), 116.6 (d, 1C), 111.4 (d, 1C), 111.3 (d, 1C), 110.6 (s, 1C), 109.8 (s, 1C), 73.8 (t, 1C), 67.3 (t, 1C), 58.6 (q, 1C), 55.2 (d, 1C), 53.8 (d, 1C), 53.5 (d, 1C), 36.9 (t, 1C), 28.2 (q, 3C), 27.30 (t, 1C), 27.29 (t, 1C), 3 singlets were not observed; HRMS (MALDI-TOF) *m/z* [M + H]^+^ calculated for C_45_H_49_N_5_O_8_ + Na^+^ 810.3479; found 810.3480.

### 3.5. Quantum Yield of Methanolysis

Quantum yields for the photomethanolysis reactions for **3** and **3×HCl** were determined by using KI/KIO_3_ (Φ_254_ = 0.74) actinometer [58,60], as recently described by us [79]. Solutions of peptides in CH_3_OH and actinometer were freshly prepared and their concentrations were adjusted to have absorbances of 0.4–0.8 at 254 nm. After adjustment of the concentrations and measurement of the corresponding UV-vis spectra, the solutions were purged with a stream of N_2_ (20 min), and then, sealed with a cap. The cells were placed in a holder which ensured equal distance of all samples from the lamp and were irradiated at the same time in the reactor with 1 lamp at 254 nm for 10 min. Before and after the irradiation, the samples were taken from the cells using a syringe and analyzed by HPLC to determine the photochemical conversions. The conversion did not exceed 30% to avoid a change of the absorbance, or filtering of the light by the product. From the conversion of actinometer, irradiance was calculated according to Equations (S1)–(S5) reported in the SI. The average value of three measurements was reported.

### 3.6. Irradiation of N-Boc-L-Trp-L-Trp-L-Tyr[CH_2_N(CH_3_)_2_]-OBn (3) in the Presence of Nucleotides

Quartz NMR tubes or fluorescence cells were filled with a solution of **3** (1 mM) in CD_3_CN-D_2_O (1:1, *v*/*v*) or CH_3_CN-H_2_O (1:1, *v*/*v*), which contained different polynucleotide (10 mM): 2′-deoxyadenosine; 2′-deoxycytidine; 2′-deoxyguanidine, 2′-deoxyadenosine-5′-diphosphate; 2′-deoxycytidine-5′-monophosphate, and 2′-deoxyguanidine-5′-monophosphate. The solutions were irradiated in a Luzchem reactor equipped with 8 lamps with the maximum output at 300 nm (1 lamp 8W) for 15 min, 30 min, or 45 min, followed by the analysis by NMR or HPLC and HPLC-MS.

### 3.7. Absorption and Fluorescence Measurements

Absorption spectra were recorded on a PG T80/T80+ or a Varian Cary 100 Bio spectrophotometer at rt. Fluorescence measurements were performed on an Agilent Cary Eclipse fluorometer by using slits corresponding to the bandpass of 10 nm for the excitation and the emission. The samples were dissolved in EtOAc, THF, CH_3_CN, or CH_3_CN-H_2_O (1:9) and the concentrations were adjusted to absorbances of less than 0.1 at the excitation wavelengths of 280, 290, or 300 nm. Fluorescence quantum yields were determined by comparison of the integral of the emission bands with the one of NATA in H_2_O (Φ_f_ = 0.14) [58]. One fluorescence measurement was performed by exciting sample at three different wavelengths and the average value was calculated (Equation (S5) in the SI). Prior to the measurements, the solutions were purged with Ar for 15 min. The measurement was performed at rt (25 °C).

TC-SPC measurements were performed on an Edinburgh FS5 spectrometer equipped with a pulsed LED at 280 nm. The duration of the pulse was ≈800 ps. Fluorescence signals at 370 nm were monitored over 1023 channels with the time increment of ≈20 ps/channel. The decays were collected until they reached 3000 counts in the peak channel. A suspension of silica gel in H_2_O was used as a scattering solution to obtain instrument response function (IRF). Absorbances at 280 nm were 0.07–0.09. Prior to the measurements the solutions were purged with a stream of nitrogen for 20 min. The measurement was performed at rt (25 °C). Decays of fluorescence were fit to a sum of exponentials according to Equation (S7) in the SI, using software implemented with the instrument.

### 3.8. Preparation of the Annealed Double Stranded Oligonucleotides

Stock solutions of dG_12_, dC_12_, dA_10_, and dT_10_ (*c* = 1.0 × 10^−3^ M), were prepared in ammonium acetate buffer (100 mM, pH 7.0). The solution of dG_12_ (250 μL) was mixed with the solution of dC_12_ (250 μL) in a UV cuvette and their mixture was heated to 90 °C for 5 min, and slowly cooled to rt and stored for 24 h at 4 °C before the use in experiments. Analogously, the solution of dA_10_ (180 μL) was mixed with the solution of dT_10_ (180 μL) in a UV cuvette, heated to 90 °C for 5 min, slowly cooled to rt and stored for 24 h at 4 °C before using. The solutions of annealed oligonucleotides were used in the thermal denaturation experiments, CD and photochemical alkylation experiments.

### 3.9. Thermal Denaturation Experiments

A stock solution of **3×HCl** was prepared in mQ H_2_O (*c* = 1.74 × 10^−3^ M), and a stock solution of ct-DNA *c*(ct-DNA) = 1.6 × 10^−2^ M was used. The solution of ct-DNA was sonicated and filtered (pores 0.45 μm). In the denaturation experiments, the ct-DNA solution was diluted in a quartz UV-vis cell (with the optical path of 1.0 cm) by cacodylate buffer to the concentration of *c* = 3.0 × 10^−5^ M, and the appropriate amount of the solution of **3×HCl** was added to reach the desired ratio *r* ([**3×HCl**]/[ct-DNA]) = 0.3. For the thermal denaturation experiments of annealed oligonucleotides dG_12_-dC_12_ or dA_10_-dT_10_ the stock solutions were diluted with ammonium acetate buffer (pH = 7.0, 100 mM) to concentrations of *c* = 2.0 × 10^−5^ M. The dependence of the absorbance at 260 nm as a function of temperature was measured on a Cary 100 Bio (Agilent Varian, Santa Clara, CA, USA) UV-vis spectrometer. The temperature was varied from 10 °C to 98 °C in intervals of 0.5 °C. The denaturation temperature *T*_m_ values are the midpoints of the transition curves, determined from the maximum of the first derivative [72]. ∆*T*_m_ values were calculated by subtracting *T*_m_ of the free nucleic acid from that of the respective complex with ∆*T*_m_ values (Equation (S7) in the SI) are the average of at least two independent measurements and the error in ∆*T*_m_ is ca. ± 0.5 °C.

### 3.10. Fluorescence Titrations

Stock solutions of solution **1×HCl**–**3×HCl** were prepared in DMSO (*c* = 5 × 10^−3^ M). For the titrations, the stock solutions were diluted in a fluorescence cell (3 mL) with cacodylate (pH 7.0, 50 mM) to reach the concentration *c* = 2.0–6.0 × 10^−6^ M, thus having <1% DMSO. Polynucleotide stock solutions were *c* = 1–2 × 10^−4^ M. The fluorescence spectra were measured on a Cary Eclipse (Agilent Technologies, Santa Clara, CA, USA) at 25 °C. The samples were excited at 295 nm, and the emission was recorded in the range 300–600 nm. The excitation slit was set to the bandpass of 10 nm, and the emission slit to 20 nm. Small aliquots of the solutions of polynucleotides were added to the solution of **1×HCl**–**3×HCl** and after an incubation time of 2 min, fluorescence spectra were taken. Data obtained by fluorescence titrations were processed by nonlinear regression analysis according to the Scatchard model, McGhee, von Hippel formalism [68]. Each titration with ct-DNA was performed five times, and with other polynucleotides at least twice.

### 3.11. Circular Dichroism Spectroscopy

Circular dichroism spectra were measured on a Jasco J-815 spectrometer in quartz cells with the optical path of 1 cm at 25 °C. To the polynucleotide solutions in cells (*c* = 3.0 × 10^−5^ M) in cacodylate buffer (pH = 7.0, 50 mM), aliquots of the solution of **1×HCl**–**3×HCl** in buffer (*c* = 1.0 × 10^−3^ M) were added to reach the concentration ratio *r*[**1×HCl**–**3×HCl**]/[polynucleotide] = 0.1–0.7. For the measurement of CD spectra of annealed oligonucleotides dG_12_-dC_12_ or dA_10_-dT_10_ the stock solutions were diluted with ammonium acetate buffer (pH = 7.0, 100 mM) to concentrations of *c* = 2.0 × 10^−5^ M. The CD spectra were recorded in the wavelength range 220–400 nm with the scanning rate of 200 nm/min and with two accumulations.

### 3.12. Photochemical Alkylation ct-DNA and of Oligonucleotides

ct-DNA: A stock solution of **3×HCl** (*c* = 1.0 × 10^−2^ M) was prepared by dissolving 1.0 mg in DMSO (100 μL), whereas the stock solutions of ct-DNA was prepared in aqueous cacodylate buffer (pH = 7.0, 50 mM) *c*(ct-DNA) = 1.6 × 10^−2^ M. DNA and **3×HCl** were mixed in a UV cuvette as described in the thermal denaturation experiment and exposed to light (Luzchem reactor equipped with 8 lamps with the output at 300 nm over 15 min), and thermal denaturation of ds-DNA was performed.

The stock solution of **3×HCl** was diluted with ammonium acetate buffer (100 mM, pH = 7.0) to the concentration of *c* = 5.0 × 10^−3^ M. The solution of **3×HCl** (100 µL, *c* = 5.0 × 10^−3^ M) was mixed with the annealed solution of dG_12_-dC_12_ (340 µL, *c* = 5.0 × 10^−4^ M) in a quartz UV-vis cell (1 mL). In another cell, the solution of **3×HCl** (60 µL, *c* = 5.0 × 10^−3^ M) was mixed with the annealed solution of dA_10_-dT_10_ (260 µL, *c* = 5.0 × 10^−4^ M). The cells were irradiated in a Luzchem reactor equipped with 8 lamps with the output at 300 nm over 15 min and 60 min. The composition of the irradiated solutions was analyzed by HPLC equipped with a PLRP-S 5 µm column and diode array detector. Details on the chromatographic method can be found in the SI (Table S4). Experiments were performed twice.

### 3.13. Laser Flash Photolysis (LFP)

The measurements were performed on a LP980 Edinburgh Instruments spectrophotometer. For the excitation the fourth harmonic of a Q-smart Q450 Quantel YAG laser at 266 nm was used. The energy of the laser pulse at 266 nm was set to 20 mJ and the pulse duration was 7 ns. Absorbances at the excitation wavelength were set to 0.3–0.4. The static cells were used and they were frequently exchanged to assure no absorption of light by photoproducts. At least three decays were collected to determine the decay kinetics, and the average values were reported, whereas the quoted errors correspond to maximal standard deviations. The solutions were purged for 15 min with Ar or O_2_ prior to the measurements, which were conducted at 25 °C.

## 4. Conclusions

We have demonstrated that photochemically reactive modified tyrosine can be incorporated in peptides containing Trp amino acids, where it remains photochemically reactive (Φ_R_ = 0.11–0.13), whereas the peptide retains the Trp fluorescence (Φ_f_ = 0.03–0.06). The intrinsic fluorescence properties of Trp residues can be used for the study of peptide interactions with polynucleotides. The affinities of **2×HCl** and **3×HCl** to all studied ds-polynucleotides were similar (log*K*_a_ = 6.0–6.8). However, **3×HCl** fluorescence spectral responses were strongly dependent on the base pair composition: the GC-containing polynucleotides efficiently quenched Trp emission, at variance to AT- or AU-polynucleotides, which induced bisignate response. Namely, addition of AT(U) polynucleotides at excess over studied peptide induced emission quenching (attributed to Trp-aggregation in the grooves of polynucleotides), whereas at excess of DNA/RNA over peptide, Trp-fluorescence increase was observed. This result suggested that studied peptides bind into the DNA minor groove, deep inserted into the AT-sequence and much superficially inserted into the sterically blocked GC-sequence.

Upon UV-irradiation of the photochemically reactive peptide **3×HCl** bound to ds-oligonucleotides, only QM-induced alkylation of dA_10_-dT_10_ was detected, whereas no covalent reaction with dG_12_-dC_12_ was observed. This reactivity pattern is not the typical for QMs, showing that non-covalent binding and positioning of the QM precursor within the polynucleotide-binding site might be the deciding factor for the QM reactivity. It seems that just deep insertion into the AT-DNA minor groove ensured effective alkylation, at variance to the GC-DNA binding, in which peptide is significantly more exposed to aqueous environment.

Therefore, our investigations show a proof of principle that QM precursor can be introduced into a peptide and used as a photochemical switch to enable primarily non-covalent interaction and consequently light-induced alkylation of ds-DNA, enabling further applications and important impact in chemistry and biology.

## Figures and Tables

**Table 1:** Quantum yields of fluorescence Φ_f_ and fluorescence decay times (τ_f_).

Compound	Φ_f_ (CH_3_CN) ^1^	τ_f_ (CH_3_CN) ^2^/ns	Compound	Φ_f_ (CH_3_CN-H_2_O) ^3^	τ_f_ (CH_3_CN-H_2_O)/ns
**1**	0.11 ± 0.03	0.12 ± 0.03 (4%) 3.49 ± 0.06 (96%)	**1×HCl**	0.06 ± 0.01	-
**2**	0.11 ± 0.02	0.14 ± 0.03 (5%) 2.69 ± 0.06 (95%)	**2×HCl**	0.06 ± 0.01	0.36 ± 0.03 (7%) 2.63 ± 0.07 (93%)
**3**	0.06 ± 0.01	0.24 ± 0.03 (7%) 2.37 ± 0.06 (93%)	**3×HCl**	0.03 ± 0.01	≈0.03 (11%) 1.62 ± 0.05 (60%) 2.9 ± 0.1 (29%)

^1^ Quantum yield of fluorescence was measured using NATA in mQ-H_2_O (Φ_f_ = 0.14) [58]. The fluorescence spectra were measured by exciting at 280, 290, and 300 nm, and the average values of the quantum yields (Equation (S1) in the SI) were calculated. The associated errors correspond to the maximum absolute deviation. ^2^ Decay times were measured by TC-SPC. Fluorescence decays were obtained by exciting samples at 280 nm and detecting fluorescence at 370 nm. Relative contribution of decay components is given in parenthesis. The associated errors correspond to maximal standard deviations obtained from the fitting. ^3^ CH_3_CN-H_2_O (1:9), containing sodium cacodylate buffer (pH = 7.0, 50 mM).

**Table 2:** Binding constants and spectroscopic properties of complexes (log *K*_a_ ^1^/Int ^2^) of **1**–**3×HCl** with ds-polynucleotides, calculated by processing fluorometric titrations (*c* = 3 × 10^−6^ M), at pH = 7.0, sodium cacodylate buffer 50 mM.

	ct-DNA	*p*(dAdT) ^1^	*p*(dGdC) ^2^	pApU
**1×HCl**	5.7/0.62 ^3^	-	-	-
**2×HCl**	6.8/0.69	- ^4^	6.5/0.3	5.0/3 ^5^
**3×HCl**	6.5/0.24	5.9/1.02	6.4/0.48	6.0/1.48

^1^ Processing of the titration data by Scatchard equation gave values of ratio *n*[bound dye]/[polynucleotide] = 0.2 and 0.4, for easier comparison all log *K*_a_ values were re-calculated for fixed *n* = 0.3. Thus, the error of binding constant value varies within the half order of magnitude, and only differences of at least one order of magnitude can be considered as significant. Correlation coefficients were >0.98 for all calculated *K*_a_. Minimally two fluorescence titrations were performed (see details in the experimental). ^2^  Int—ratio of fluorescence intensity of the dye/polynucleotide complex calculated by Scatchard equation, divided by fluorescence intensity of the dye. ^3^ Performed in 20% DMSO/buffer, and therefore, not comparable to other values obtained in buffer. ^4^ Too small emission changes for accurate processing of data. ^5^ Almost linear increase of emission allowed only approximation of log*K*_a_ and Int.

## Data Availability

The data presented in this study are available in supplementary material.

## Supplementary Materials

The following are available online, Synthetic procedures for the preparation of known precursors, fluorescence spectra, LFP data, non-covalent binding to DNA, covalent binding to oligonucleotides, and copies of ^1^H and ^13^C NMR spectra.

## Sample Availability

Samples of the compounds are not available from the authors.
