# Supplementary material for: Non-Covalent Binding of Tripeptides-Containing Tryptophan to Polynucleotides and Photochemical Deamination of Modified Tyrosine to Quinone Methide Leading to Covalent Attachment
Source: Molecules. 2021 Jul 16;26(14):4315. doi: 10.3390/molecules26144315 (PMC8306964; doi:10.3390/molecules26144315)
Supplement: Supplementary file 1 [file molecules-26-04315-s001.zip › molecules-1291283-supplementary.pdf]

**Electronic supporting information**

**for**

# Noncovalent binding of tripeptides containing tryptophan to polynucleotides and photochemical deamination of modified tyrosine to quinone methide leading to covalent attachment


Antonija Erben,[a] Igor Sviben,[a] Branka Mihaljević,[b] Ivo Piantanida,[a] Nikola Basarić[a]*

[a] Department of Organic Chemistry and Biochemistry, Ruđer Bošković Institute, Bijenička cesta 54, 10 000 Zagreb, Croatia. Fax: + 385 1 4680 195; Tel: +385 1 4680 196

[b] Department of Material Chemistry, Ruđer Bošković Institute, Bijenička cesta 54, 10 000 Zagreb

Corresponding author's E-mail address: nbasaric@irb.hr


Content:


1. Synthetic procedure for the preparation of known compounds (Eqs S1-S5, Tables S1 and S2) S2
2. UV-vis and fluorescence data (Figs S1-S10, Eq S6 and S7) S9
3. Laser flash photolysis (Figs S11-S35) S14
4. Non-covalent binding to polynucleotides (Figs S38-S54, Table S3, Eq S8) S25
5. Covalent binding to oligonucleotides (Fig S55-S75 and Table S4) S35
6. NMR spectra S46
7. References S61

## 1. Synthetic procedure for the preparation of known compounds

### *N*-Boc-L-Tyr[$CH_2N(CH_3)_2$]-OBn[1]

A flask was charged with *N*-Boc-L-Tyr-OBn (1.11 g, 3.0 mmol) dissolved in $CH_2Cl_2$ (150 mL). To the reaction mixture, $K_2CO_3$ (0.21 g, 1.5 mmol) and Eschenmoser's salt (0.28 g, 3.0 mmol) were added and the reaction was stirred at rt for 5 days. When the reaction was finished, the reaction mixture was filtered through a sinter funnel, and from the filtrate the solvent was removed on a rotary evaporator. The product was purified by column chromatography on aluminum oxide (III) using 0→5% $CH_3OH$ in $CH_2Cl_2$to afford the pure product (0.72 g, 56 %) in the form of colorless crystals.

$^1$H NMR ($CD_3OD$, 300 MHz) *δ*/ppm: 7.35-7.22 (m, 5H), 6.91 (dd, *J*= 1.6, 8.0 Hz, 1H), 6.84 (d, *J*= 1.6 Hz, 1H), 6.64 (d, *J*= 8.0 Hz, 1H), 5.12 (d, *J*= 12.0 Hz, 1H), 5.06 (d, *J*= 12.0 Hz, 1H), 4.37-4.23 (m, 1H), 3.53 (s, 2H), 2.96 (dd, *J*= 6.4, 13.6 Hz, 1H), 2.84 (dd, *J*= 13.6, 8.0 Hz, 1H), 2.26 (s, 6H), 1.38 (s, 9H); $^{13}$C NMR ($CD_3OD$, 75 MHz) *δ*/ppm: 173.7 (s, 1C), 157.8 (s, 1C), 157.7 (s, 1C), 137.1 (s, 1C), 131.1 (d, 2C), 130.5 (d, 3C), 129.5 (d, 1C), 129.3 (d, 1C), 128.6 (s, 1C), 123.5 (s, 1C), 116.6 (d, 1C), 80.6 (s, 1C), 67.8 (t, 1C), 62.4 (t, 1C), 56.9 (d, 1C), 44.7 (q, 2C), 37.9 (t, 1C), 28.6 (q, 3C).

### TFA×H-L-Tyr[$CH_2N(CH_3)_2$×TFA]-OBn[1]

*N*-Boc-L-Tyr[$CH_2N(CH_3)_2$]-OBn (335 mg, 0.8 mmol) was dissolved in $CH_2Cl_2$ (3 mL). TFA/$CH_2Cl_2$ (1:1, 4 mL) was added and the reaction mixture was stirred at rt 2 h. The solvent was removed by distillation in vacuum to afford oily product quantitatively.

$^1$H NMR ($CD_3OD$, 300 MHz) *δ*/ppm: 7.41-7.28 (m, 5H), 7.14-7.09 (m, 2H), 6.86 (d, *J*= 8.8 Hz, 1H), 5.26 (d, *J*= 11.5 Hz, 1H), 5.20 (d, *J*= 11.5 Hz, 1H), 4.31 (dd (t), *J*= 7.0 Hz), 4.22 (d, *J*= 12.9 Hz, 1H), 4.13 (d, *J*= 12.9 Hz, 1H), 3.15 (d, *J*= 7.5 Hz, 2H), 2.79 (d, *J*= 7.5 Hz, 6H); $^{13}$C NMR ($CD_3OD$, 75 MHz) *δ*/ppm: 169.9 (s, 1C), 157.5 (s, 1C), 136.2 (s, 1C), 134.4 (d, 1C), 134.0 (d, 1C), 129.9 (d, 1C), 129.8 (d, 2C), 129.7 (d, 2C), 126.6 (s, 1C), 118.0 (s, 1C), 117.0 (d, 1C), 69.3 (t, 1C), 58.2 (t, 1C), 55.2 (d, 1C), 43.2 (q, 2C), 36.5 (t, 1C).

### *N*-Boc-L-Trp-OSu[2]

*N*-Boc-L-Trp (5.00 g, 16.4 mmol), *N*-hydroxysuccinimide (NHS, 2.07 g, 18.0 mmol) and *N,N′*-dicyclohexylcarbodiimide (DCC, 3.72 g, 18.0 mmol) were dissolved in $CH_2Cl_2$ (40 mL). The reaction mixture was stirred for 2 h and cooled by an ice bath, and after that kept in a refrigerator overnight. The precipitate was filtered off and the solvent was removed on a rotary evaporator.

The residue was suspended in EtOAc (100 mL) and left to stand in a refrigerator. After filtration, the solution was washed with aqueous $Na_2CO_3$ (0.5 M, 100 mL), HCl (0.5 M, 100 mL) and water (100 mL). The organic solution was dried over anhydrous $MgSO_4$, filtered and the solvent was removed on a rotary evaporator to afford yellowish product (6.07 g, 89%), which was used in the next step without purification.

$^1$H NMR ($CD_3OD$, 300 MHz) $\delta$/ppm: 7.58 (d, $J$ = 7.5 Hz, 1H), 7.33 (d, $J$ = 8.0 Hz, 1H), 7.22 (br. s, 1H), 7.10 (dd (t), $J$ = 8.0 Hz, 1H), 7.03 (dd (t), $J$ = 8.0 Hz, 1H), 4.80 (dd, $J$ = 5.0, 8.0 Hz, 1H), 3.47 (dd, $J$ = 5.0, 14.7 Hz, 1H), 3.22 (dd, $J$ = 8.0, 14.7 Hz, 1H), 2.84 (s, 4H), 1.36 (s, 9H).

***N*-Boc-L-Trp- L-Trp-OH**[3]

A solution of *N*-Boc-L-Trp-OSu (2.21 g, 5.5 mmol) in THF (25 mL) was added slowly to the suspension of L-Trp (1.02 g, 5.0 mmol) and $NaHCO_3$ (0.84 g, 10.0 mmol) in a mixture of THF and $H_2O$ (1:1, 48 mL). The reaction mixture was stirred at rt for 3 days. THF was removed on a rotary evaporator and the aqueous residue acidified with HCl (0.5 M) to pH 2-3. An extraction with EtOAc (3×50 mL) was carried out, the extracts were dried over anhydrous $Na_2SO_4$, filtered and the solvent was removed on a rotary evaporator to afford product in the form of colorless solid (2.34 g, 95%).

$^1$H NMR ($CD_3OD$, 300 MHz) $\delta$/ppm: 7.57 (d, $J$ = 7.9 Hz, 1H), 7.32 (dd (t), $J$ = 7.9 Hz, 3H), 7.13-6.96 (m, 5H), 6.90 (dd (t), $J$ = 6.5 Hz 1H), 4.77-4.67 (m, 1H), 4.37-4.27 (m, 1H), 3.25-3.13 (m, 3H), 3.09-2.97 (m, 1H), 1.28 (s, 9H); $^{13}$C NMR ($CD_3OD$, 75 MHz) $\delta$/ppm: 174.9 (s, 1C), 174.4 (s, 1C), 157.4 (s, 1C), 138.1 (s, 1C), 137.9 (s, 1C), 128.9 (s, 1C), 128.8 (s, 1C), 124.7 (d, 1C), 124.5 (d, 1C), 122.42 (d, 1C), 122.40 (d, 1C), 119.85 (d, 1C), 119.81 (d, 1C), 119.4 (d, 1C), 119.2 (d, 1C), 112.3 (d, 1C), 112.2 (d, 1C), 110.9 (s, 1C), 110.4 (s, 1C), 80.7 (s, 1C), 56.7 (d, 1C), 54.6 (d, 1C), 28.9 (t, 1C), 28.5 (q, 3C), 28.3 (t, 1C).

***N*-Boc-L-Trp-L-Trp-OSu**[2]

*N*-Boc-L-Trp-L-Trp (0.96 g, 2.0 mmol), NHS (0.25 g, 2.2 mmol) and DCC (0.34 g, 2.2 mmol) were dissolved in $CH_2Cl_2$ (15 mL). The reaction mixture was stirred for 2 h and cooled by an ice bath, and after that kept in a refrigerator overnight. The reaction mixture was washed with aqueous $Na_2CO_3$ (0.5 M, 75 mL), HCl (0.5 M, 75 mL) and water (75 mL). The organic solution was dried over anhydrous $Na_2SO_4$, filtered and the solvent was removed on a rotary evaporator to afford product in the form of colorless solid (0.70 g, 60%), which was used in the next step without purification.

$^1$H NMR ($CD_3OD$, 300 MHz) $\delta$/ppm: 7.55 (d, $J$ = 7.7 Hz, 1H), 7.37-7.26 (m, 2H), 7.20 (br. s, 1H), 7.14-6.80 (m, 6H), 5.13 (dd (t), $J$ = 6.1 Hz, 1H), 4.37-4.25 (m, 1H), 3.35-3.00 (m, 4H), 2.81 (s, 4H), 1.25 (s, 9H).

**Synthesis of tripeptides – general procedure**

A solution of *N*-Boc-L-Trp-L-Trp-OSu (387 mg, 0.65 mmol) in THF-u (6 mL) was added slowly to a suspension of the salt TFA×H-L-Tyr-OBn or TFA×H-L-Phe-OBn (0.6 mmol) and $NaHCO_3$ (252 mg, 3.0 mmol) in a mixture of THF and $H_2O$ (1:1, 12 mL). The reaction mixture was stirred at rt over 3 days. THF was removed on a rotary evaporator and the aqueous residue was acidified by HCl (0.5 M) to the pH value of 2-3. The extraction with EtOAc (3×50 mL) was carried out and the extracts were dried over anhydrous sodium sulfate. After filtration, the solvent was removed on a rotary evaporator and the crude residue was purified on a column of silica gel using $CH_3OH/CH_2Cl_2$ (1→20%) as eluent to afford the pure oily product.

***N*-Boc-L-Trp- L-Trp-L-Phe-OBn (1)**[3]

Prepared according to the general procedure from *N*-Boc-L-Trp-L-Trp-OH (387 mg, 0,65 mmol) and TFA×H-L-Phe-OBn (0.24 g, 0.6 mmol). After column chromatography, the oily product was obtained (0.25 g, 30%).

$^1$H NMR ($CD_3OD$, 300 MHz)$\delta$/ppm: 7.56 (d, $J$= 8.0 Hz, 1H), 7.36 (d, $J$= 8.4 Hz, 1H), 7.33-7.26 (m, 4H), 7.24-7.18 (m, 2H), 7.18-7.12 (m, 4H), 7.12-7.07 (m, 2H), 7.05-6.97 (m, 4H), 6.93-6.86 (m, 1H), 6.82 (br. s, 1H), 5.04 (d, $J$= 12.0 Hz, 1H), 4.98 (d, $J$= 12.0 Hz, 1H), 4.61-4.52 (m, 2H), 4.23 (dd (t), $J$= 5.6 Hz, 1H), 3.18-3.02 (m, 3H), 2.97-2.87 (m, 1H), 2.83-2.67 (m, 2H), 1.22 (s, 9H); $^{13}$C NMR ($CD_3OD$, 150 MHz) $\delta$/ppm: 174.3 (s, 1C), 173.4 (s, 1C), 172.1 (s, 1C), 138.1 (s, 1C), 138.0 (s, 1C), 137.7 (s, 1C), 137.0 (s, 1C), 130.2 (d, 2C), 129.5 (d, 4C), 129.4 (d, 2C), 129.3 (d, 1C), 128.9 (s, 1C), 128.8 (s, 1C), 127.9 (d, 1C), 124.9 (d, 1C), 124.8 (d, 1C), 122.6 (d, 1C), 122.5 (d, 1C), 120.0 (d, 2C), 119.5 (d, 1C), 119.2 (d, 1C), 112.4 (d, 2C), 110.7 (s, 1C), 110.0 (s, 1C), 80.9 (s, 1C), 67.9 (t, 1C), 57.1 (d, 1C), 55.6 (d, 1C), 55.1 (d, 1C), 38.6 (t, 1C), 28.6 (t, 1C),28.5 (q, 3C), 28.1 (t, 1C).

***N*-Boc-L-Trp-L-Trp-L-Tyr-OBn (2)**[3]

Prepared according to the general procedure from *N*-Boc-L-Trp-L-Trp-OH (387 mg, 0.65 mmol) and TFA×H-L-Tyr-OBn (0.23 g, 0.6 mmol). After column chromatography, the oily product was obtained (0.13 g, 30%).

$^{1}H$ NMR ($CD_3OD$, 300 MHz)*δ*/ppm: 7.56 (d, *J*= 7.8 Hz, 1H), 7.39-7.24 (m, 5H), 7.24-7.16 (m, 1H), 7.16-6.96 (m, 6H), 6.96-6.76 (m, 4H), 6.62 (d, *J*= 8.4 Hz, 2H), 5.03 (d, *J*= 13.0 Hz, 1H), 4.99 (d, *J*= 13.0 Hz, 1H), 4.60-4.46 (m, 2H), 4.24 (dd (t), *J*= 6.5 Hz, 1H), 3.19-3.02 (m, 3H), 2.87-2.65 (m, 3H), 1.22 (s, 9H); $^{13}C$ NMR ($CD_3OD$, 75 MHz) *δ*/ppm: 174.6 (s, 1C), 173.5 (s, 1C), 172.5 (s, 1C), 157.4 (s, 1C), 138.04 (s, 1C), 138.00 (s, 1C), 137.0 (s, 1C), 131.4 (d, 2C), 129.5 (d, 2C), 129.3 (d, 1C), 129.2 (d, 1C), 128.8 (s, 1C), 128.7 (d, 1C), 128.3 (s, 1C), 124.7 (d, 1C), 124.6 (d, 1C), 122.5 (d, 2C), 120.0 (d, 1C), 119.9 (d, 1C), 119.44 (d, 1C), 119.40 (d, 1C), 116.3 (d, 1C), 112.3 (d, 2C), 110.9 (s, 1C), 110.3 (s, 1C), 80.7 (s, 1C), 67.4 (t, 1C), 57.5 (d, 1C), 55.8 (d, 1C), 55.1 (d, 1C), 37.8 (t, 1C), 29.0 (t, 1C), 28.7 (q, 3C), 28.5 (t, 1C).

**Removal of Boc – general procedure**

Tripeptide **1**-**3** (0.03 mmol) was disolved in anhydrous EtOAc to which a saturated solution of HCl in EtOAc was added (5 M, 1 mL). The reaction mixture was stirred 1 h at rt and the solvent was removed in vacuum. The remaining crystals were washed with $Et_2O$ and dried on a rotary evaporator. The product was purified by preparative TLC on silica gel using 20% MeOH/$CH_2Cl_2$ as eluent or by semipreparative HPLC.

**HCl×H-L-Trp-L-Trp-L-Phe-OBn (1×HCl)**[3]

Prepared according to the general procedure from **1** (20 mg, 0.03 mmol) giving the oily product (10 mg, 55%).

$^{1}H$ NMR ($CD_3OD$, 300 MHz)*δ*/ppm: 7.64 (d, *J*= 8.1 Hz, 1H), 7.58 (d, *J*= 7.8 Hz, 1H), 7.37 (dd (t), *J*= 8.1 Hz, 2H), 7.33-7.27 (m, 3H), 7.26-7.21 (m, 3H), 7.20-7.15 (m, 3H), 7.14-7.05 (m, 6H), 7.04-6.96 (m, 3H), 5.08 (d, *J*= 12,1 Hz, 1H), 5.02 (d, *J*= 12,1 Hz, 1H), 4.73 (dd (t), *J*= 7.0 Hz, 1H), 4.65 (dd, *J*=6.4, 7.8 Hz, 1H), 4.07 (dd, *J*=5.6, 8.7 Hz, 1H), 3.21 (dd, *J*=6.4, 14.7 Hz, 1H), 3.15-3.02 (m, 3H), 2.94 (dd, *J*= 7.8, 13.7 Hz, 1H);$^{13}C$ NMR ($CD_3OD$, 150 MHz) *δ*/ppm: 173.2 (s, 1C), 172.3 (s, 1C), 169.8 (s, 1C), 138.3 (s, 1C), 138.1 (s, 1C), 137.8 (s, 1C), 136.9 (s, 1C), 130.4 (d, 1C), 129.5 (d, 3C), 129.4 (d, 1C), 128.7 (s, 1C), 128.2 (s, 1C), 127.9 (d, 1C), 125.8 (d, 1C), 124.8 (d, 1C), 122.9 (d, 1C), 122.5 (d, 1C), 120.3 (d, 1C), 119.8 (d, 1C), 119.4 (d, 1C), 119.3 (d, 1C), 119.1 (d, 1C), 112.6 (d, 1C), 112.4 (d, 1C), 110.5 (s, 1C), 107.9 (s, 1C), 68.0 (t, 1C), 55.55 (d, 1C), 55.53 (d, 1C), 54.7 (d, 1C), 38.4 (t, 1C), 29.2 (t, 1C), 28.9 (t, 1C).

**HCl×H-L-Trp-L-Trp-L-Tyr-OBn (2×HCl)**[3]

Prepared according to the general procedure from **2** (20 mg, 0.03 mmol) giving the oily product (11 mg, 61%).

**$^1$H NMR** ($CD_3OD$, 600 MHz)$\delta$/ppm: 7.61 (d, *J*= 8.0 Hz, 1H), 7.44 (d, *J*= 8.0 Hz, 1H), 7.36 (d, *J*= 8.0 Hz, 1H), 7.34-7.29 (m, 4H), 7.22 (dd, *J*= 1.8, 7.0 Hz, 2H), 7.11 (dd(t), *J*= 7.5 Hz 1H), 7.09-7.05 (m, 2H), 7.02 (dd(t), *J*= 7.5 Hz 1H), 6.97-6.93 (m, 2H), 6.85 (d, *J*= 8.3 Hz, 2H), 6.65 (d, *J*= 8.3 Hz, 2H), 5.05 (d, *J*= 12.0 Hz, 1H), 5.03 (d, *J*= 12.0 Hz, 1H), 4.69 (dd(t), *J*= 6.9 Hz, 1H), 4.57 (dd(t), *J*= 7.0 Hz, 1H), 3.85 (dd(t), *J*=6.5 Hz, 1H), 3.21 (dd, *J*=5.5, 14.6 Hz, 1H), 3.10-3.00 (m, 4H), 2.91 (dd, *J*=6.5, 14.0 Hz, 1H), 2.82 (dd, *J*=7.4, 14.0 Hz, 1H); $^{13}$C NMR ($CD_3OD$, 150 MHz) $\delta$/ppm: 176.2 (s, 1C), 173.5 (s, 1C), 172.4 (s, 1C), 157.4 (s, 1C), 138.2 (s, 1C), 138.0 (s, 1C), 131.4 (d, 2C), 129.52 (d, 2C), 129.51 (d, 2C), 129.3 (d, 1C), 128.85 (s, 1C), 128.83 (s, 1C), 128.2 (s, 1C), 125.0 (d, 1C), 124.8 (d, 1C), 122.6 (d, 1C), 122.4 (d, 1C), 120.0 (d, 1C), 119.8 (d, 1C), 119.5 (d, 1C), 119.4 (d, 1C), 116.3 (d, 1C), 112.4 (d, 1C), 112.3 (d, 1C), 110.6 (s, 1C), 110.3 (s, 1C), 67.9 (t, 1C), 56.1 (d, 1C), 55.5 (d, 1C), 55.0 (d, 1C), 37.7 (t, 1C), 31.1 (t, 1C), 28.8 (t, 1C).

**HPLC analysis and separation**

Analysis of the samples was performed by HPLC on a Phenomenex Luna C18

Table S1. The following method was used:

| *t* / min | B (%) |
|---|---|
| 0 | 100 |
| 10 | 100 |
| 20 | 0 |
| 30 | 0 |

A: $CH_3OH$, 100 %

B: $CH_3OH$-$H_2O$ (1:1 v/v) + TFA, 0.1%

Flow rate: 1 mL/ min.

Semipreparative HPLC separation on a Phenomenex Jupiter C18 5μ 300A column

Table S2. The following method was used:

| $t$ / min | B (%) |
|---|---|
| 0 | 100 |
| 20 | 100 |
| 40 | 0 |
| 60 | 0 |

A: $CH_3OH$, 100 %

B: $CH_3OH$-$H_2O$ (1:1 v/v) + TFA, 0.1%

Flow rate: 1 mL/ min.

**Determination of the quantum yield of photomethanolysis**

The **number of absorbed photons for the $KIO_3$/KI** was calculated from:

$$n(\text{absorbed photons}) = \frac{\Delta A_{352} \times V_{irr}}{\varepsilon_{352} \times \ell \times \Phi_{lit.}} \quad \text{(S1)}$$

where:

| | |
|---|---|
| $\Delta A_{352}$ | absorbance difference at 352 nm for the irradiated and non-irradiated sample |
| $V_{\text{irr}}$ | volume of the solution which was irradiated |
| $\varepsilon_{352}$ | molar absorption coefficient for $I_3^-$ in solution which contains iodides and iodates, 27600 $M^{-1}$ $cm^{-1}$ |
| $l$ | length of the optical path (1 cm in all experiments) |
| $\Phi_{\text{lit.}}$ | quantum yield ($\Phi_{254} \approx 0.74$), the precise value was calculated from S2 and S3 (depending on the iodine concentration and temperature) |

$$c(\text{I}^-) = A_{300}/1.061 \quad [\text{M}] \quad \text{(S2)}$$

$$\Phi_{\text{lit}} = 0.75 \times [1 + 0.02(T - 20.7)] \times [1+0.23(c(\text{I}^-) - 0.577)] \quad \text{(S3)}$$

**For the absorbances in the range 0.4-0.8 the number of absorbed photons was calculated according to:**

$$n(\text{absorbed photons}) = n(\text{total photons}) \times (1\text{-}T) \qquad \text{(S4)}$$

**The quantum yield for the photohydrolysis was calculated according to**:

$$\Phi = \frac{A_{254} \cdot V_{\text{irr}} \cdot x(\text{photoproduct})_{\text{HPLC}}}{\varepsilon_{254} \cdot \ell \cdot n(\text{total photons}) \cdot (1 - T_{254})} \qquad \text{(S5)}$$

where:

| | |
|---|---|
| $x(\text{photoproduct})_{\text{HPLC}}$ | conversion of reactant to photoproduct determined by HPLC |
| $T_{254}$ | transmittance of light at 254 nm |

## 2. UV-vis and fluorescence data

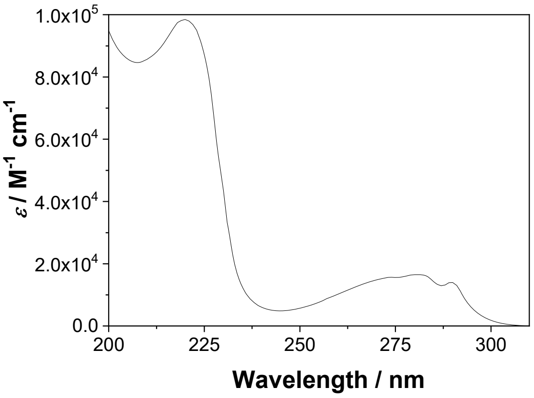


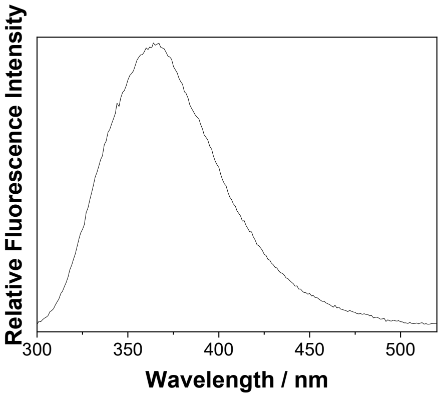


Fig. S1. Absorption spectrum (left) and emission spectrum ($\lambda_{ex}$ = 280 nm) of **1** in $CH_3CN$.

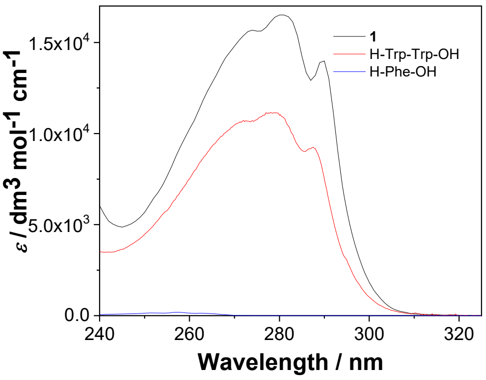


Fig. S2. Comparison of the absorptivity of **1** with the one of H-L-Trp-L-Trp-OH and H-L-Phe-OH from literature.[4]

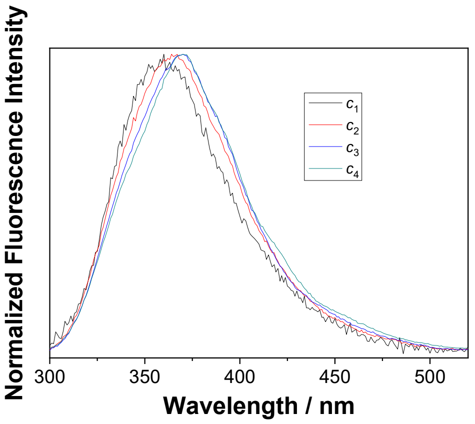


Fig. S3. Dependence of the normalized fluorescence spectra ($\lambda_{ex}$ = 280 nm) of **1** ($5\times10^{-7}$ - $2\times10^{-6}$ M), in aqueous solution in the presence of sodium cacodylate buffer (pH 7.0, 50 mM).

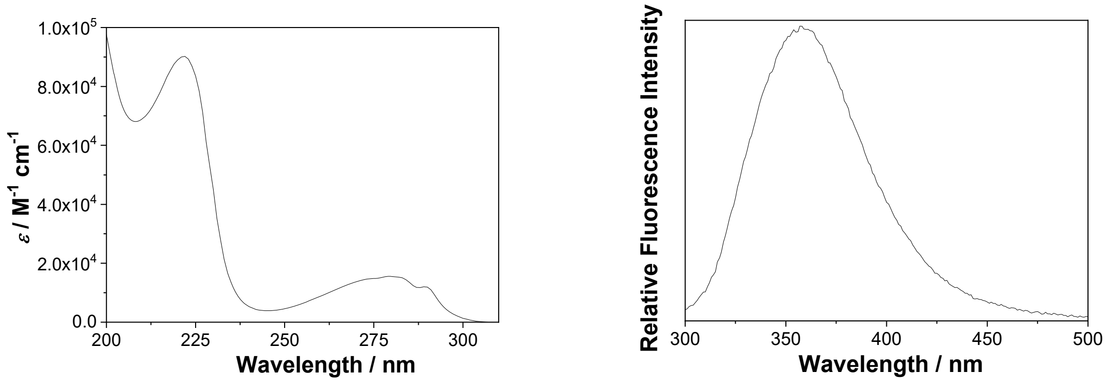


Fig. S4. Absorption spectrum (left) and emission spectrum ($\lambda_{ex}$ = 280 nm) of **2** in $CH_3CN$.

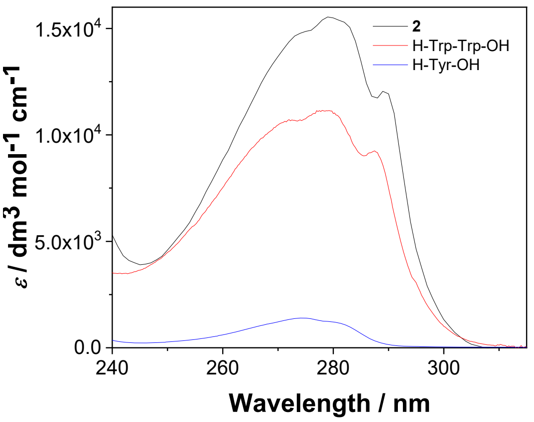


Fig. S5. Comparison of the absorptivity of **2** with the one of H-L-Trp-L-Trp-OH and H-L-Tyr-OH from literature.[5]

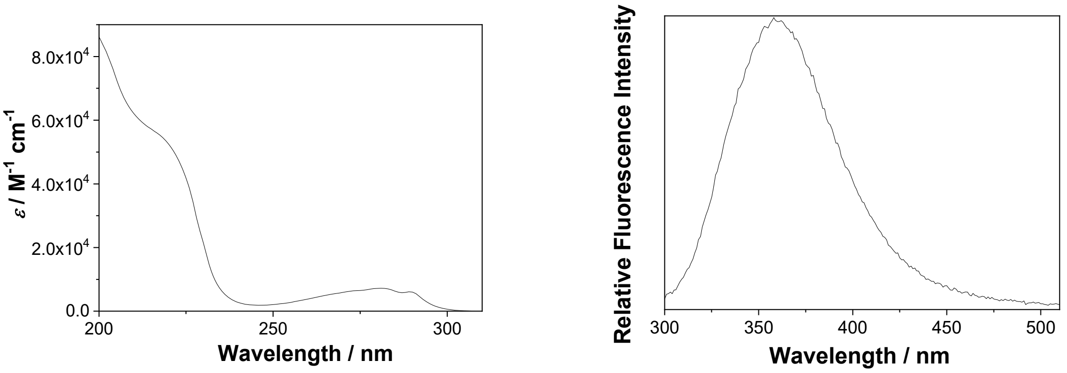


Fig. S6. Absorption spectrum (left) and emission spectrum ($\lambda_{ex}$ = 280 nm) of **3** in $CH_3CN$.

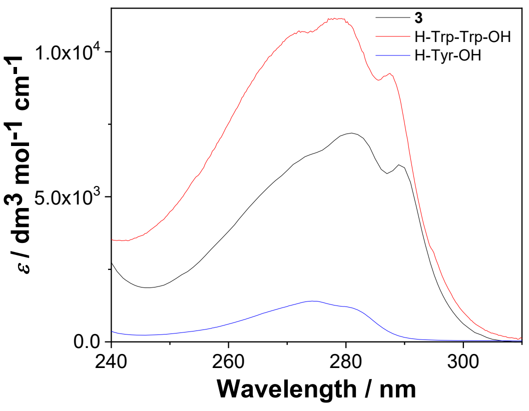


Fig. S7. Comparison of the absorptivity of **3** with the one of H- L-Trp-L-Trp-OH and H-L-Tyr-OH from literature.[5]

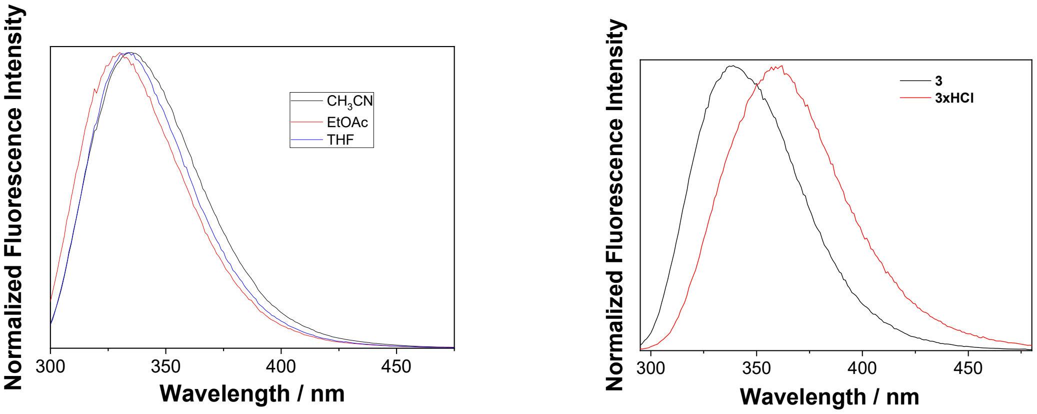


Fig. S8. Normalized fluorescence spectra ($\lambda_{ex}$ = 280 nm) of **3** in $CH_3CN$, EtOAc and THF (left), and comparison of the fluorescence spectra of **3** in $CH_3CN$ and **3×HCl** in $CH_3CN$-$H_2O$ (1:9), sodium cacodilate buffer (50 mM, pH = 7.0).

<u>Fluorescence quantum yield</u> was calculated according to:

$$\Phi_f = \Phi_{Ref} \left(\frac{n}{n_R}\right)^2 \frac{I}{I_R} \frac{1-10^{-A_R}}{1-10^{-A}} \quad (S6)$$

$\Phi_f$ and $\Phi_{Ref}$ – fluorescence quantum yield of compound and the reference;

$n$ and $n_R$ – refractive index of the solvent in which compound or the reference was dissolved;

$A$ and $A_R$ – absorbance of the compound and the reference at the excitation wavelength;

$I$ and $I_R$ – area under emission curve of the compound and the reference.

Fluorescence decays were obtained with time-correlated single-photon counting method on a FS5 Edinburgh Instruments spectrometer equipped for time correlated single photon counting method (TC-SPC). Pulsed LEDs at 280 was used for the excitation (pulse duration ≈ 800 ps). Fluorescence signals were monitored over 1023 channels with the time increment of ≈ 20 ps/channel. The decays were collected until they reached 3000 counts in the peak channel. The histograms were analyzed by a nonlinear least-squares deconvolution method using. The quality of the fit was judged by the reduced $\chi^2$ being close to unity and the random distribution of the weighted residuals. Fluorescence decays were fit to a sum of exponentials using the following expression:

$$F\,(t) = \alpha_1 \exp\left(-\frac{t}{\tau_1}\right) + \alpha_2 \exp\left(-\frac{t}{\tau_2}\right) + \alpha_3 \exp\left(-\frac{t}{\tau_3}\right) + \;... \qquad \text{(S7)}$$

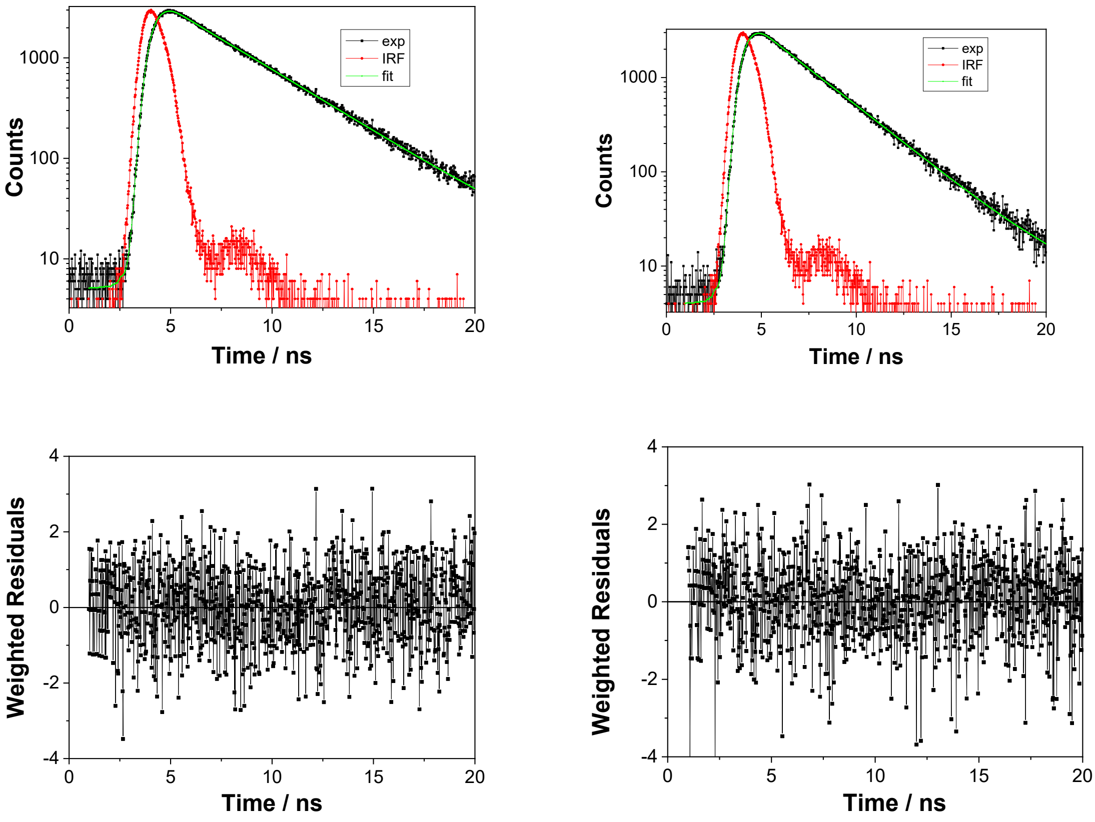


Fig. S9. Decay of fluorescence for **1** (left) and **2** (right) in $CH_3CN$ at 370 nm upon excitation at 280 nm; bottom panels show weighted residuals between the experimental and fitted values.

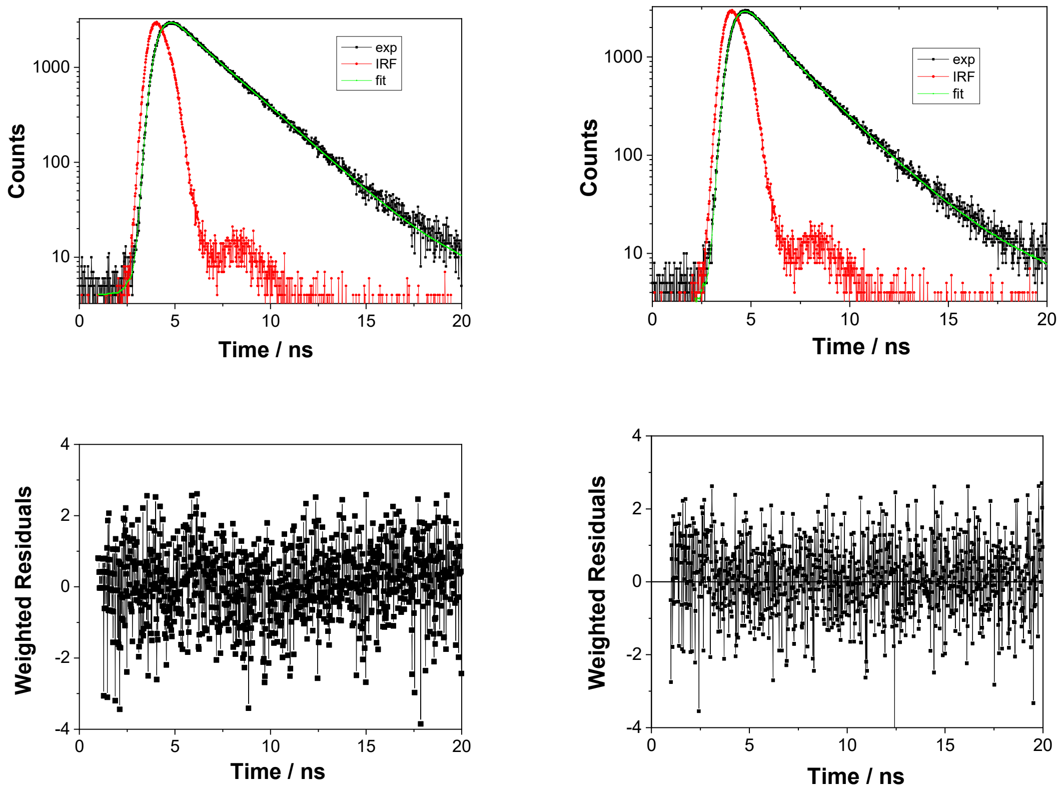


Fig. S10. Decay of fluorescence for **3** (left) in $CH_3CN$ and **3×HCl** (right) in $CH_3CN$-$H_2O$ (1:9) at 370 nm upon excitation at 280 nm; bottom panels show weighted residuals between the experimental and fitted values.

## 3. Laser Flash Photolysis

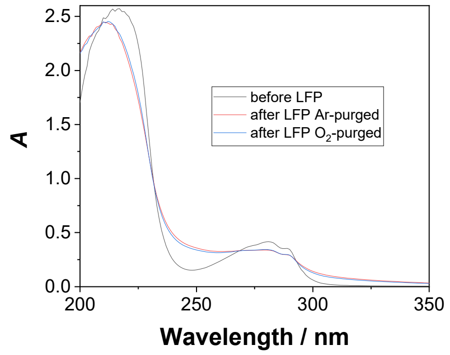


Fig. S11. UV-vis spectra of tripeptide **3** in $CH_3CN$ ($c$ = 6.1×10$^{-5}$ M) before and after the LFP experiment.

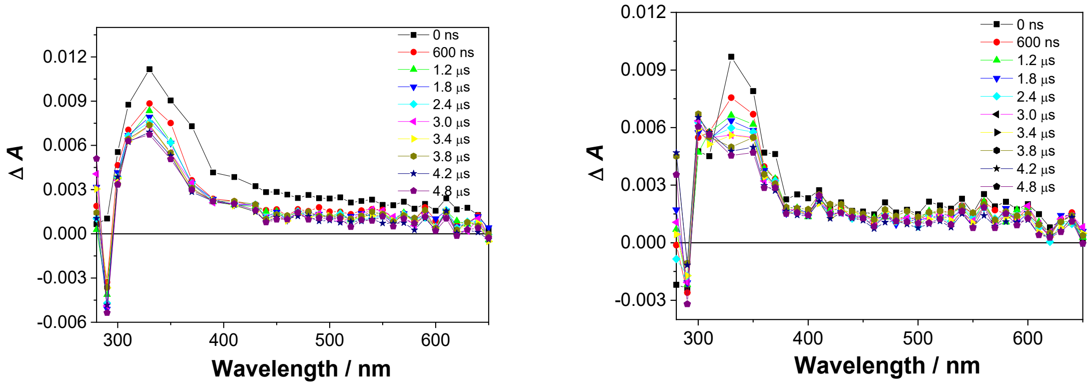


Fig. S12. Transient absorption spectra of Ar-purged (left) and $O_2$-purged (right) solution of **3** in $CH_3CN$ ($c$ = 6.1×10$^{-5}$ M), excited at 266 nm, $A_{266}$ = 0.28, laser power ≈ 18 mJ/pulse.

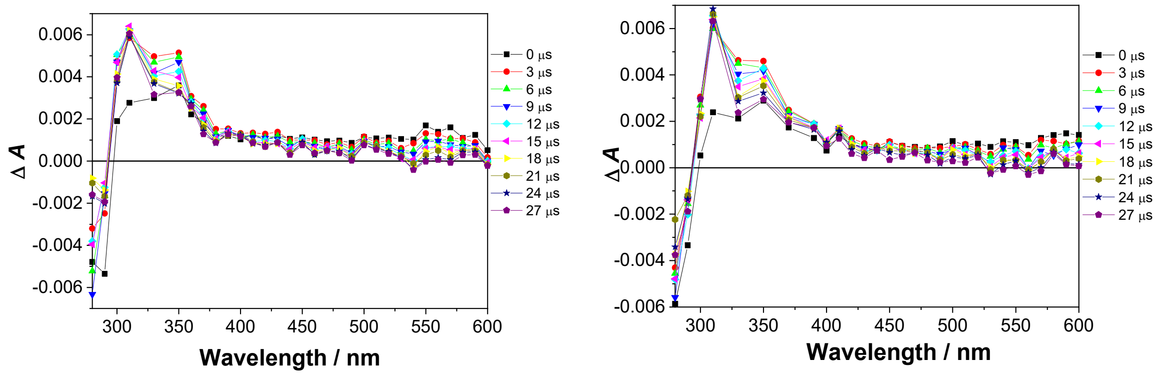


Fig. S13. Transient absorption spectra of Ar-purged (left) and $O_2$-purged (right) solution of **3** in $CH_3CN$ ($c$ = 6.1×10$^{-5}$ M), excited at 266 nm, $A_{266}$ = 0.28, laser power ≈ 18 mJ/pulse.

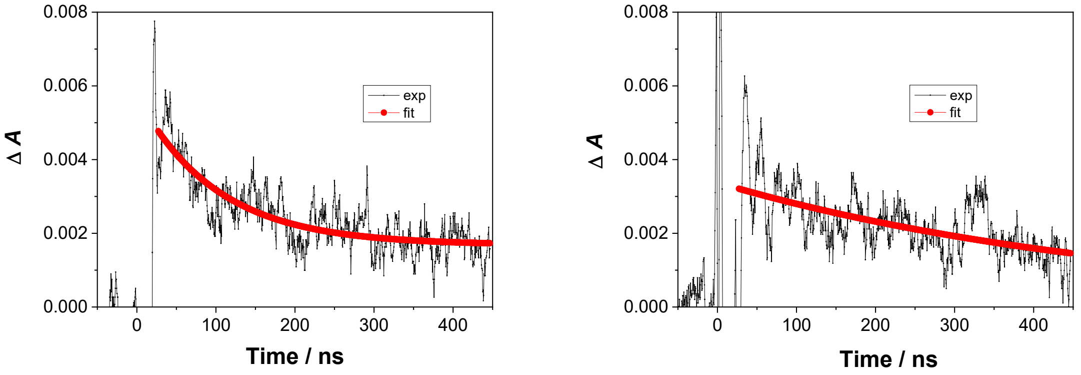


Fig. S14. Decay of transient absorption at 380 nm for Ar-purged (left) and $O_2$-purged (right) solution of **3** in $CH_3CN$ ($c = 6.1 \times 10^{-5}$ M), excited at 266 nm, $A_{266} = 0.28$.

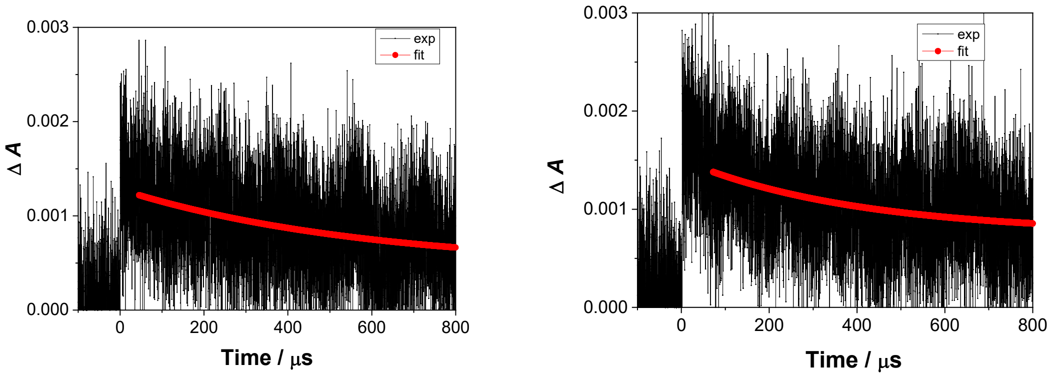


Fig. S15. Decay of transient absorption at 380 nm for Ar-purged (left) and $O_2$-purged (right) solution of **3** in $CH_3CN$ ($c = 6.1 \times 10^{-5}$ M), excited at 266 nm, $A_{266} = 0.28$.

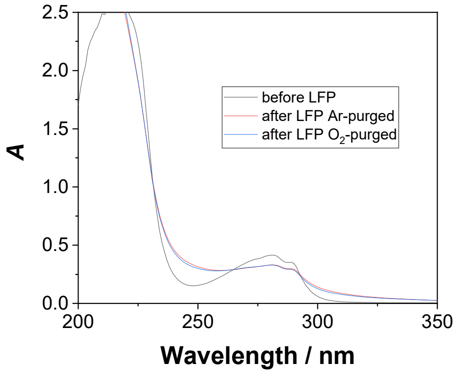


Fig. S16. UV-vis spectra of tripeptide **3** ($c$ = 6.1×10$^{-5}$ M) in $CH_3CN$-$H_2O$ (1:1 v/v, in the presence of phosphate buffer $c$ = 50 mM, pH = 7.0) before and after the LFP experiment.

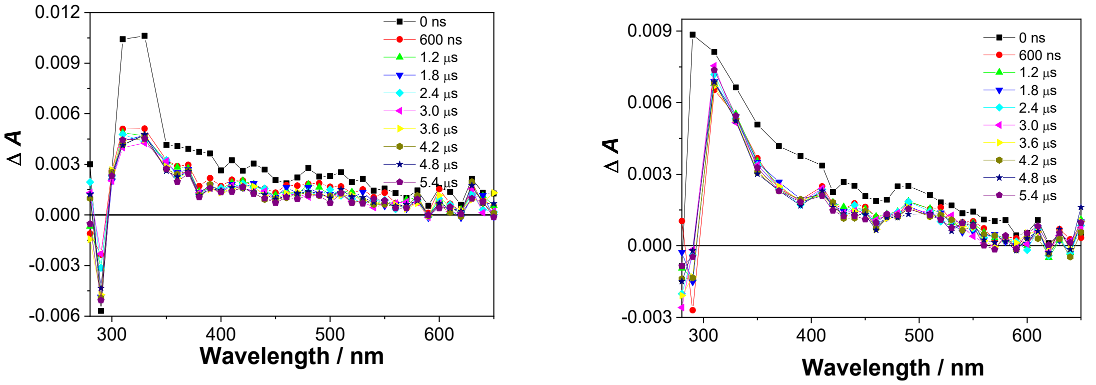


Fig. S17. Transient absorption spectra of Ar-purged (left) and $O_2$-purged (right) solution of **3** ($c$ = 6.1×10$^{-5}$ M) in $CH_3CN$-$H_2O$ (1:1 v/v, in the presence of phosphate buffer $c$ = 50 mM, pH = 7.0), excited at 266 nm, $A_{266}$ = 0.30, laser power ≈ 18-19 mJ/pulse.

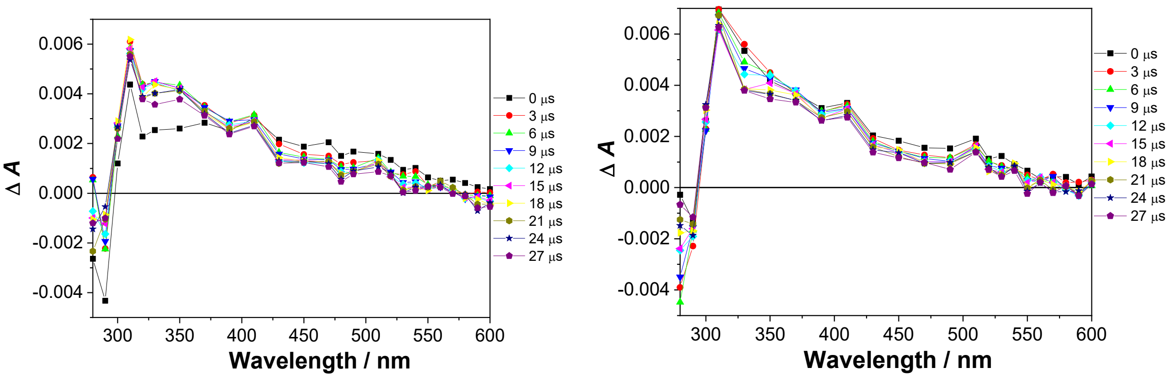


Fig. S18. Transient absorption spectra of Ar-purged (left) and $O_2$-purged (right) solution of **3** ($c$ = 6.1×10$^{-5}$ M) in $CH_3CN$-$H_2O$ (1:1 v/v, in the presence of phosphate buffer $c$ = 50 mM, pH = 7.0), excited at 266 nm, $A_{266}$ = 0.30, laser power 18-19 mJ/pulse.

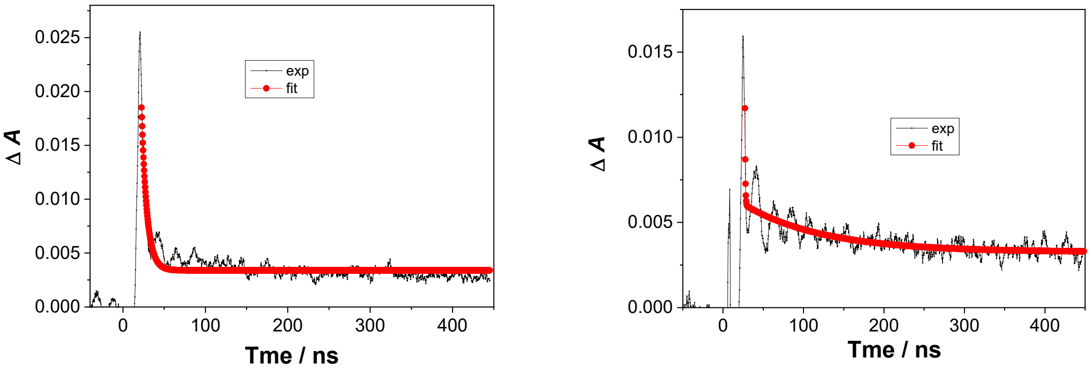


Fig. S19. Decay of transient absorption at 400 nm for Ar-purged (left) and $O_2$-purged (right) solution of **3** ($c$ = 6.1×10$^{-5}$ M) in $CH_3CN$-$H_2O$ (1:1 v/v, in the presence of phosphate buffer $c$ = 50 mM, pH = 7.0), excited at 266 nm, $A_{266}$ = 0.30.

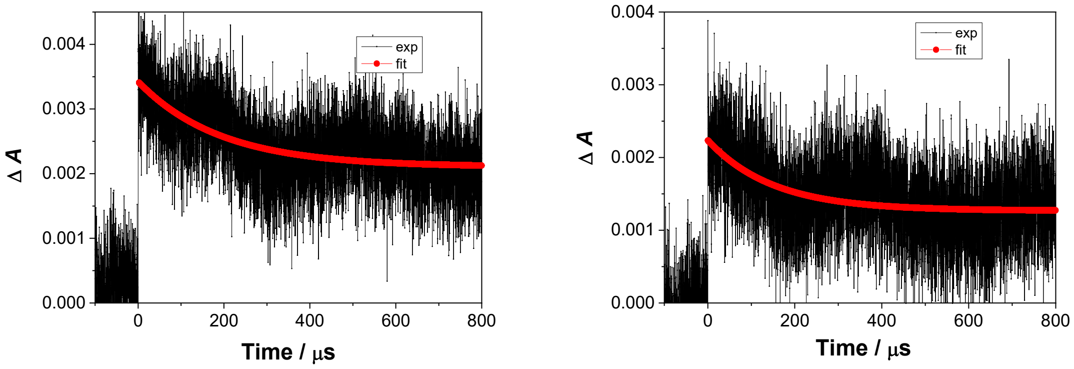


Fig. S20. Decay of transient absorption at 380 nm for Ar-purged (left) and $O_2$-purged (right) solution of **3** ($c$ = 6.1×10$^{-5}$ M) in $CH_3CN$-$H_2O$ (1:1 v/v, in the presence of phosphate buffer $c$ = 50 mM, pH = 7.0), excited at 266 nm, $A_{266}$ = 0.30.

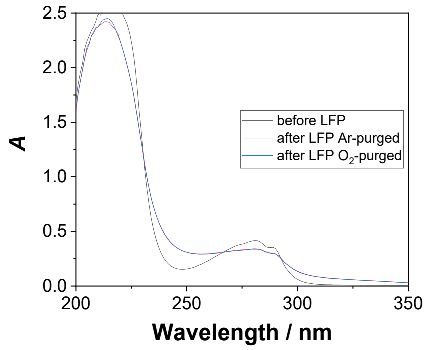


Fig. S21. UV-vis spectra of tripeptide **3×HCl** ($c$ = 9.5×10$^{-5}$ M) in $CH_3CN$-$H_2O$ (1:1 v/v, in the presence of phosphate buffer $c$ = 50 mM, pH = 7.0) before and after the LFP experiment.

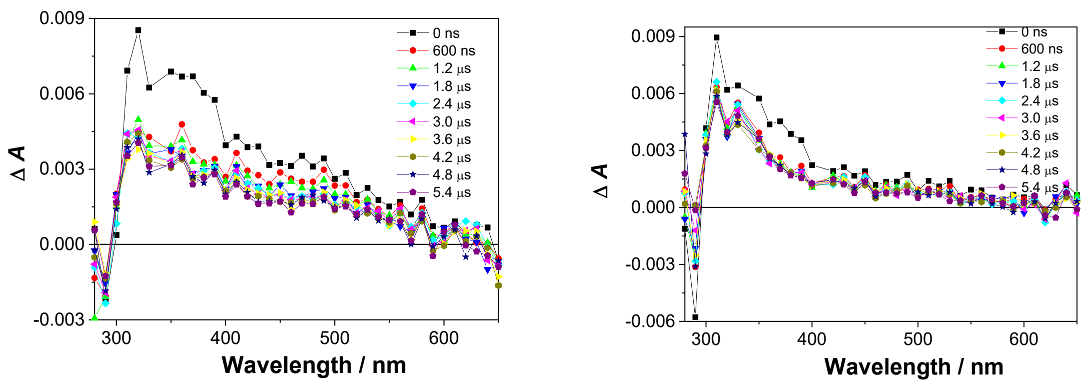


Fig. S22. Transient absorption spectra of Ar-purged (left) and $O_2$-purged (right) solution of **3×HCl** ($c$ = 9.5×10$^{-5}$ M) in $CH_3CN$-$H_2O$ (1:1 v/v, in the presence of phosphate buffer $c$ = 50 mM, pH = 7.0), excited at 266 nm, $A_{266}$ = 0.30, laser power ≈ 19 mJ/pulse.

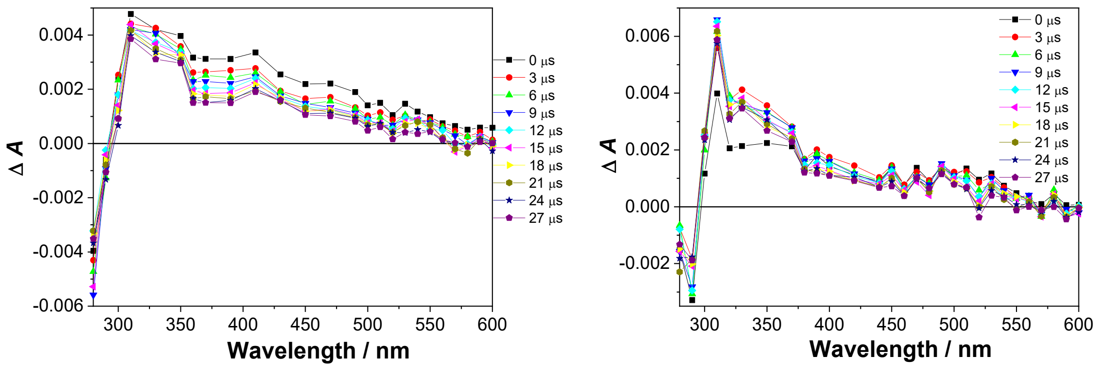


Fig. S23. Transient absorption spectra of Ar-purged (left) and $O_2$-purged (right) solution of **3×HCl** ($c$ = 9.5×10$^{-5}$ M) in $CH_3CN$-$H_2O$ (1:1 v/v, in the presence of phosphate buffer $c$ = 50 mM, pH = 7.0), excited at 266 nm, $A_{266}$ = 0.30, laser power ≈ 19 mJ/pulse.

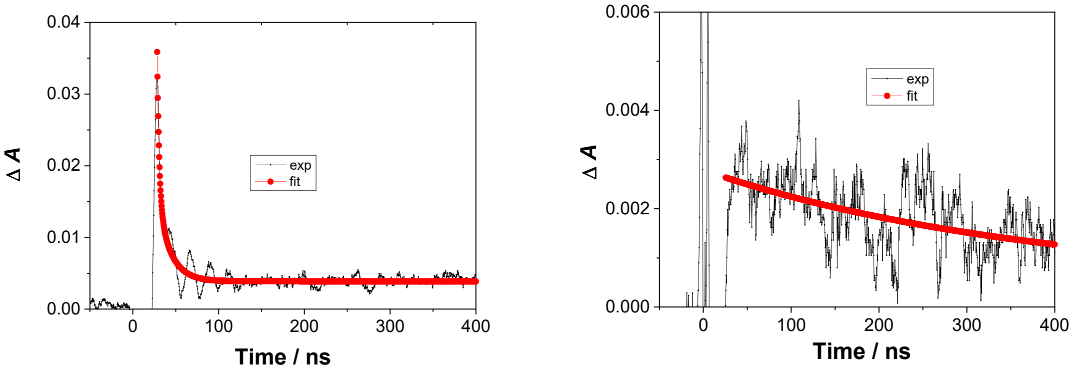


Fig. S24. Decay of transient absorption at 380 nm for Ar-purged (left) and $O_2$-purged (right) solution of **3×HCl** ($c$ = 9.5×$10^{-5}$ M) in $CH_3CN$-$H_2O$ (1:1 v/v, in the presence of phosphate buffer $c$ = 50 mM, pH = 7.0), excited at 266 nm, $A_{266}$ = 0.30, laser power ≈ 19 mJ/pulse.

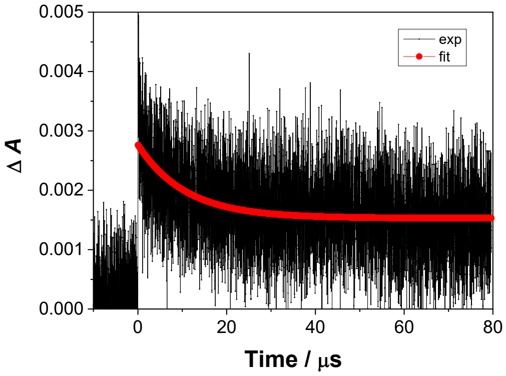


Fig. S25. Decay of transient absorption at 400 nm for Ar-purged solution of ($c$ = 9.5×$10^{-5}$ M) in $CH_3CN$-$H_2O$ (1:1 v/v, in the presence of phosphate buffer $c$ = 50 mM, pH = 7.0), excited at 266 nm, $A_{266}$ = 0.30, laser power ≈ 19 mJ/pulse.

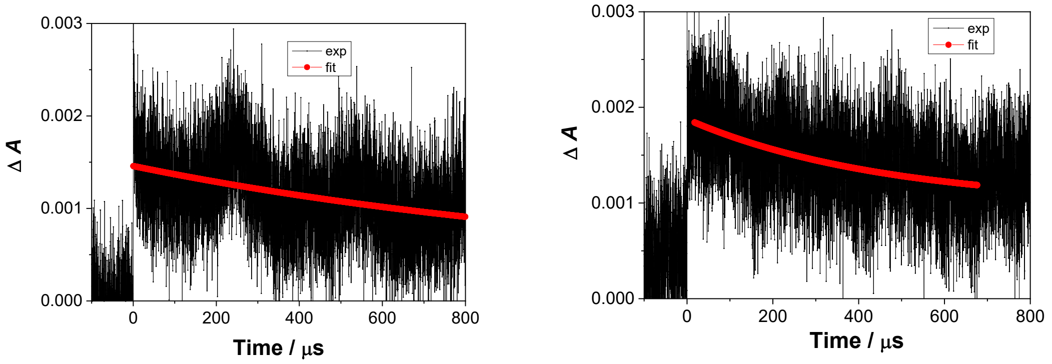


Fig. S26. Decay of transient absorption at 400 nm for Ar-purged (left) and $O_2$-purged (right) solution of **3×HCl** ($c$ = 9.5×$10^{-5}$ M) in $CH_3CN$-$H_2O$ (1:1 v/v, in the presence of phosphate buffer $c$ = 50 mM, pH = 7.0), excited at 266 nm, $A_{266}$ = 0.30, laser power ≈ 19 mJ/pulse.

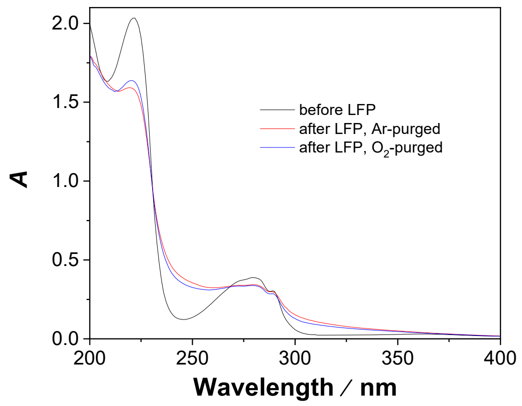


Fig. S27. UV-vis spectra of tripeptide **2** in $CH_3CN$ ($c = 2.95 \times 10^{-5}$ M) before and after the LFP experiment.

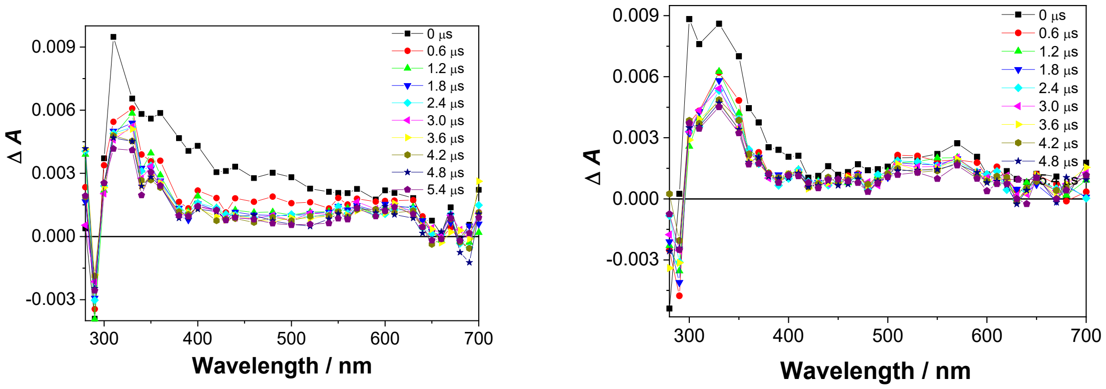


Fig. S28. Transient absorption spectra of Ar-purged (left) and $O_2$-purged (right) solution of **2** in $CH_3CN$ ($c = 2.95 \times 10^{-5}$ M), excited at 266 nm, $A_{266} = 0.31$, laser power ≈ 19 mJ/pulse.

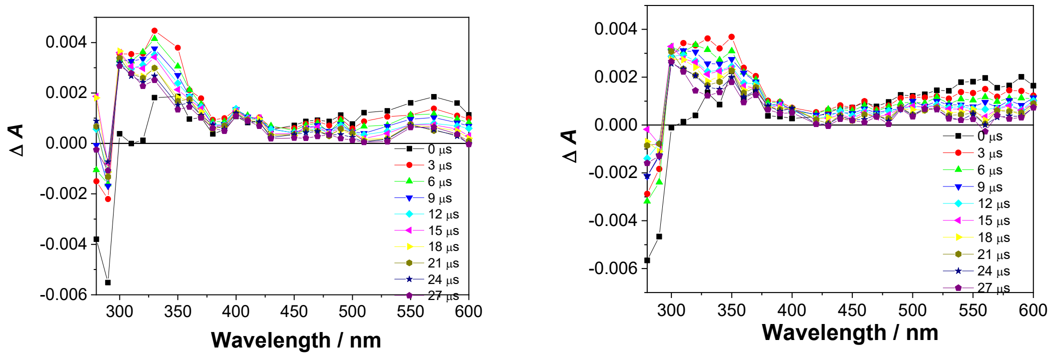


Fig. S29. Transient absorption spectra of Ar-purged (left) and $O_2$-purged (right) solution of **2** in $CH_3CN$ ($c = 2.95 \times 10^{-5}$ M), excited at 266 nm, $A_{266} = 0.31$, laser power ≈ 19 mJ/pulse.

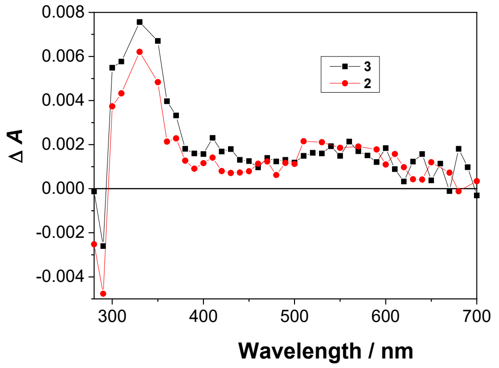


Fig. S30. Transient absorption spectra of optically matched ($A_{266}$ = 0.30) $O_2$-purged solution of **3** in $CH_3CN$ ($c = 6.1\times10^{-5}$ M) and **2** in $CH_3CN$ ($c = 2.95\times10^{-5}$ M). The spectra were measured with a delay of 600 ns, laser power ≈ 19 mJ/pulse.

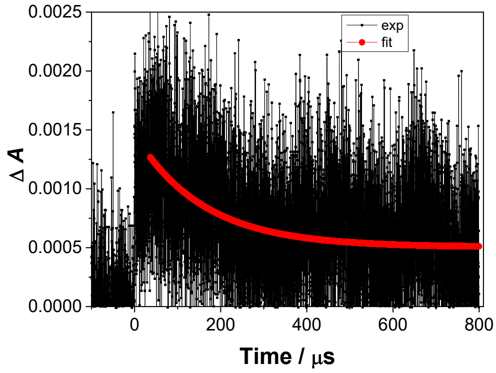


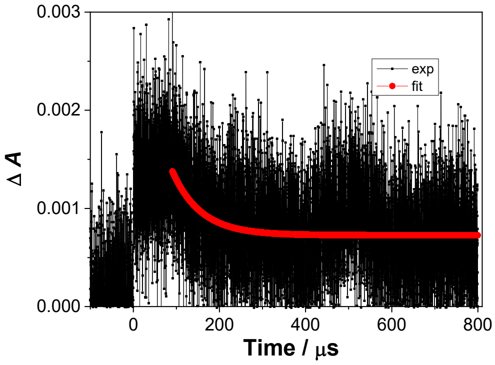


Fig. S31. Decay of transient absorption at 380 nm for Ar-purged (left) and $O_2$-purged (right) solution of **2** in $CH_3CN$ ($c = 2.95\times10^{-5}$ M), excited at 266 nm, $A_{266}$ = 0.28.

In the Ar-purged $CH_3CN$ solution of **2** we detected several transients with the lifetimes of 23±2 ns, 1.1 ±0.1 μs, 13 ± 1 μs and 120 ±40 μs. In the $O_2$-purged solution we detected transients with lifetimes 19±1 ns, 12 ± 2 μs and 55 ± 10 μs. The transient with the lifetime of 1.1 μs may therefore be assigned to the triplet excited state. The other transients were not assigned. A very long-lived transient, which was detected from **3** and tentatively assigned to QM here was not detected. Additional transient was detected at $\lambda$> 500 nm with $\tau \approx$ 9-12 μs, that was not quenched by $O_2$. It was tentatively assigned to Trp radical-cation, since it was not detected in the aqueous solvent (vide infra), and based on the literature precedent.

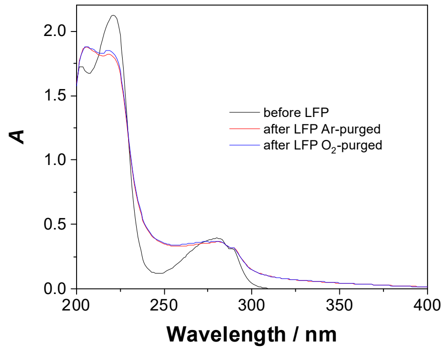


Fig. S32. UV-vis spectra of tripeptide **2** ($c$ = 3.2×10$^{-5}$ M) in $CH_3CN$-$H_2O$ (1:1 v/v, in the presence of phosphate buffer $c$ = 50 mM, pH = 7.0) before and after the LFP experiment.

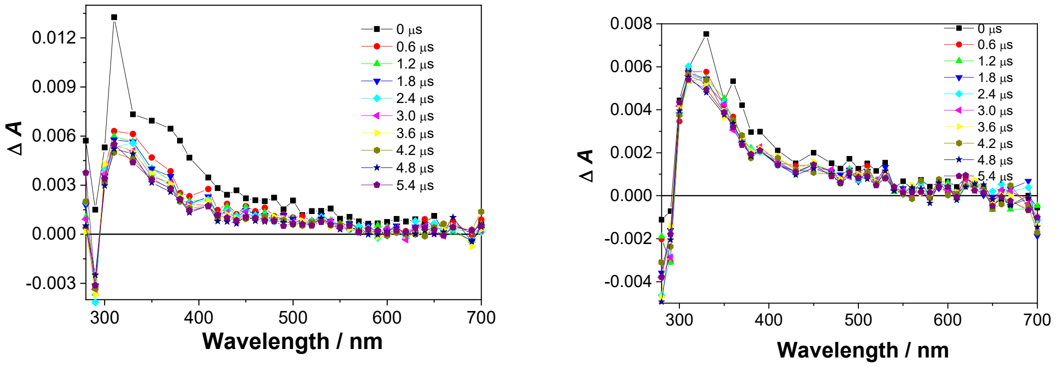


Fig. S33. Transient absorption spectra of Ar-purged (left) and $O_2$-purged (right) solution of **2** ($c$ = 3.2×10$^{-5}$ M) in $CH_3CN$-$H_2O$ (1:1 v/v, in the presence of phosphate buffer $c$ = 50 mM, pH = 7.0), excited at 266 nm, $A_{266}$ = 0.30, laser power ≈ 18 mJ/pulse.

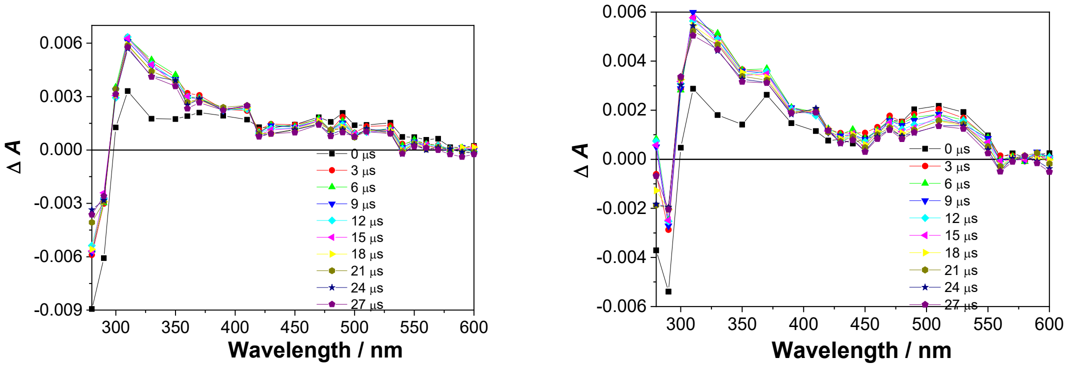


Fig. S34. Transient absorption spectra of Ar-purged (left) and $O_2$-purged (right) solution of **2** ($c$ = 3.2×10$^{-5}$ M) in $CH_3CN$-$H_2O$ (1:1 v/v, in the presence of phosphate buffer $c$ = 50 mM, pH = 7.0), excited at 266 nm, $A_{266}$ = 0.30, laser power ≈ 18 mJ/pulse.

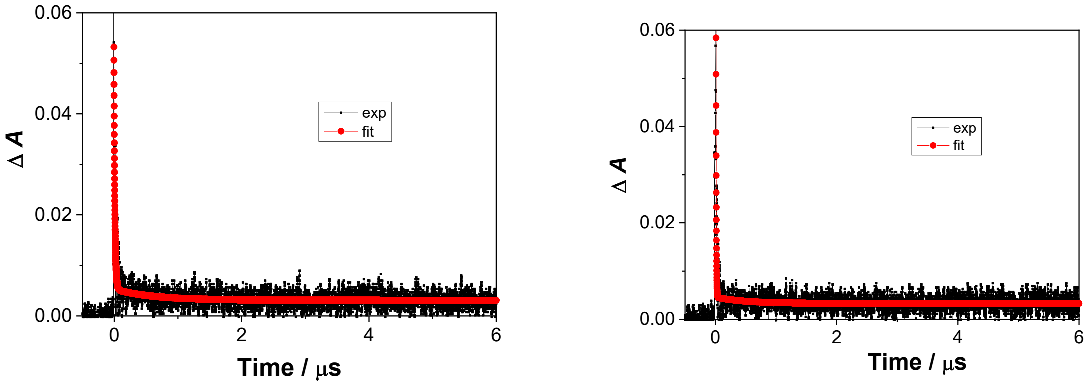


Fig. S35. Decay of transient absorption at 360 nm for Ar-purged (left) and $O_2$-purged (right) solution of **2** ($c$ = 3.2×$10^{-5}$ M) in $CH_3CN$-$H_2O$ (1:1 v/v, in the presence of phosphate buffer $c$ = 50 mM, pH = 7.0), $A_{266}$ = 0.30, laser power ≈ 18 mJ/pulse.

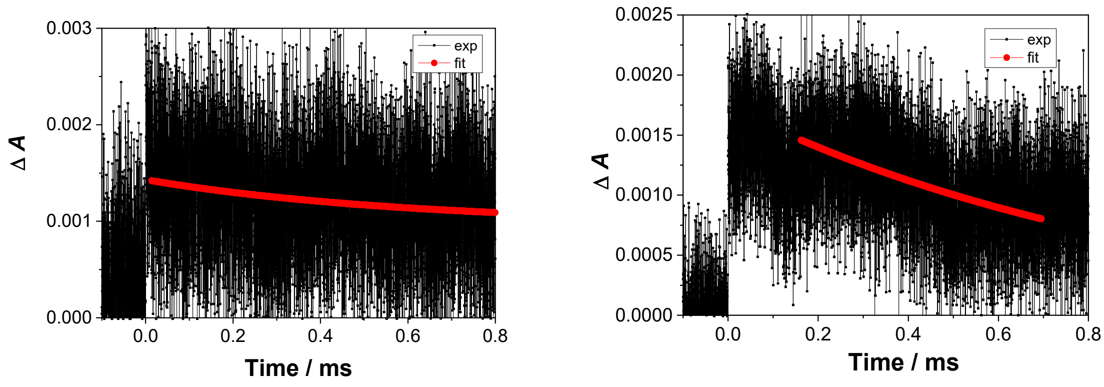


Fig. S36. Decay of transient absorption at 360 nm for Ar-purged (left) and $O_2$-purged (right) solution of **2** ($c$ = 3.2×$10^{-5}$ M) in $CH_3CN$-$H_2O$ (1:1 v/v, in the presence of phosphate buffer $c$ = 50 mM, pH = 7.0), $A_{266}$ = 0.30, laser power ≈ 18 mJ/pulse.

In the Ar-purged $CH_3CN$-$H_2O$ solution of **2** we detected several transients with the lifetimes of 14±3 ns, 500±100 ns, 13 ± 1 µs and 600 ±300 µs, and none of the transients was quenched by $O_2$. Thus, in the $O_2$-purged solution, similar transients were detectedwith lifetimes 15±5 ns, 1.1 ± 0.1 µs and 900 ± 500 µs. The transients at at $\lambda$> 500 nmwere not detected, in agreement with their assignment to Trp radical cation which in aqueous solution very readily deprotonates to the *N*-centered radical, for which quenching by $O_2$ is also not anticipated. Thus, the long-lived transient can plausibly be assigned to the Trp radical. Due to concomitant formation of Trp radical and **QM-3** the detection of **3** was more difficult and the spectra of **2** and **3** in aqueous solution are more similar (Fig S35).

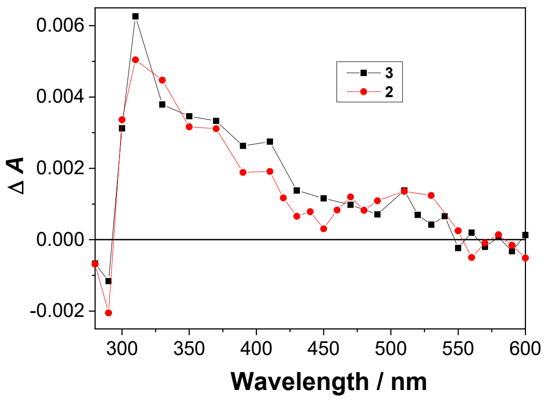


Fig. S37. Transient absorption spectra of optically matched ($A_{266}$ = 0.30) $O_2$-purged solution of **3** ($c$ = 6.1×10$^{-5}$ M) and **2** ($c$ = 3.2×10$^{-5}$ M) in $CH_3CN$-$H_2O$ (1:1 v/v, in the presence of phosphate buffer $c$ = 50 mM, pH = 7.0). The spectra were measured 600 ns after the laser pulse at 266 nm, laser power ≈ 19 mJ/pulse.

## 4. Noncovalent binding to polynucleotides

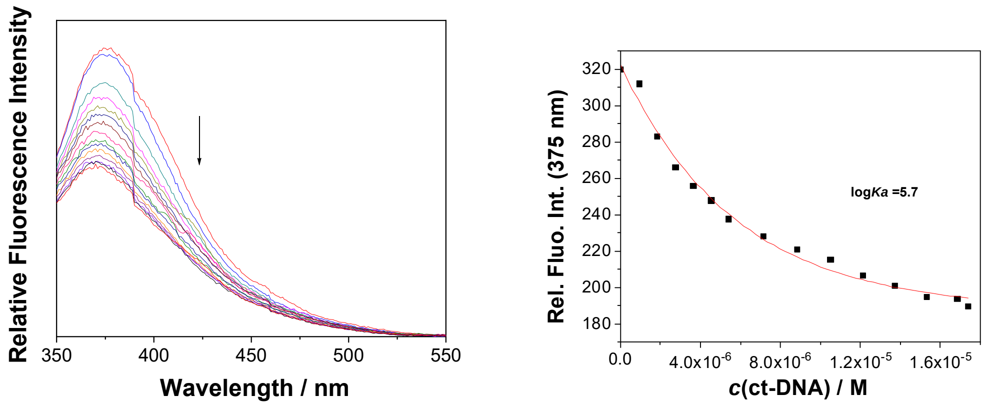


Fig. S38. Left: Fluorescence titration of **1×HCl** ($c$ = 3.3×10$^{-6}$ M, $\lambda_{exc}$ = 300 nm) with ct-DNA ($c$ = 1.2×10$^{-6}$- 6.7×10$^{-5}$ M); in cacodylate buffer (pH 7.0, 50 mM at 25 °C) with 20% DMSO. Right: Dependence of the fluorescence intensity at 375 nm on the ct-DNA concentration; dots are experimental values and the red line is calculated fit according to the Scatchard model.

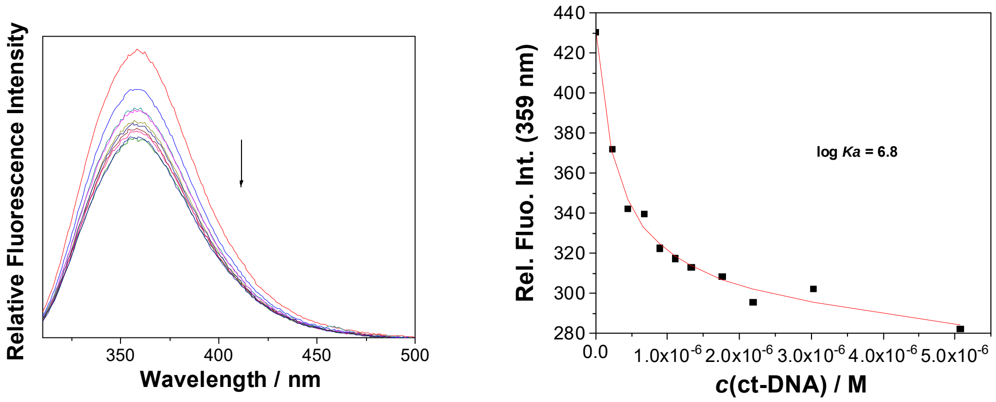


Fig. S39. Left: Fluorescence titration of **2×HCl** ($c$ = 2.4×10$^{-6}$ M, $\lambda_{exc}$ = 295 nm) with ct-DNA ($c$ = 1.2×10$^{-6}$- 6.7×10$^{-5}$ M); in cacodylate buffer (pH 7.0, 50 mM at 25 °C) with 20% DMSO. Right: Dependence of the fluorescence intensity at 359 nm on the ct-DNA concentration; dots are experimental values and the red line is calculated fit according to the Scatchard model.

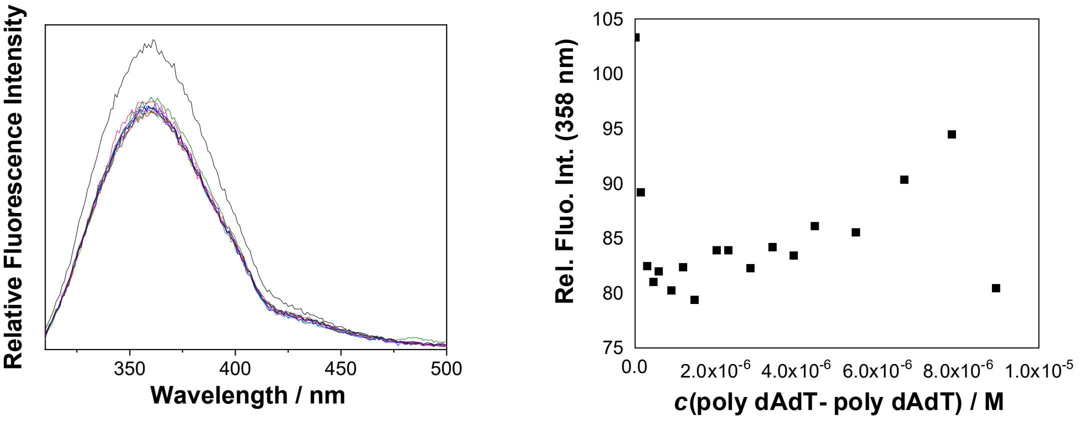


Fig. S40. Fluorescence titration of **2×HCl** ($c$ = 3.9×10$^{-6}$ M, $\lambda_{exc}$ = 295 nm) with poly dAdT - poly dAdT in cacodylate buffer (pH 7.0, 50 mM at 25 °C). Small emission changes in opposite directions (quenching at r[dye]/[DNA]>1 and emission increase for r <0.5) did not allow processing of data by Scatchard model.

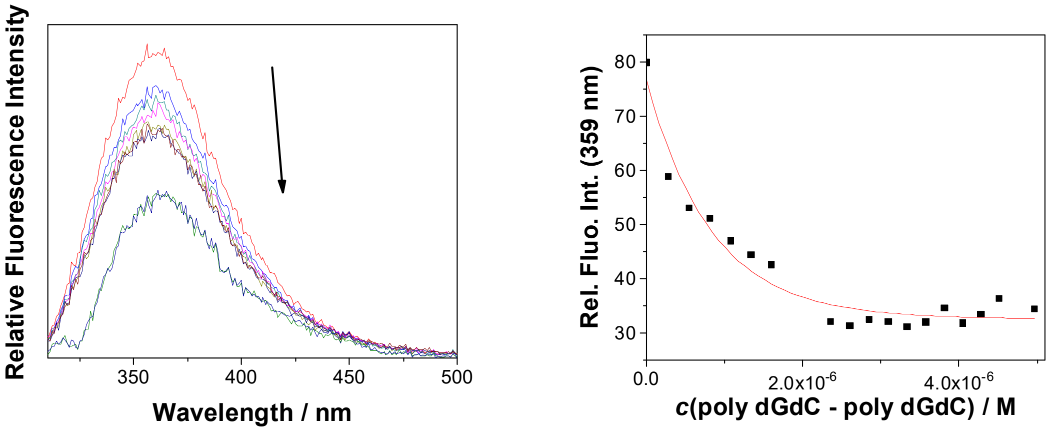


Fig. S41. LEFT: Fluorescence titration of **2×HCl** ($c$ = 3.0×10$^{-6}$ M, $\lambda_{exc}$ = 295 nm) with poly dGdC - poly dGdC in cacodylate buffer (pH 7.0, 50 mM at 25 °C); RIGHT: dependence of the fluorescence intensity (normalized to starting emission) at $\lambda_{em}$ = 359 nm on the $c$(polynucleotide), dots are experimental values and the line represents calculated fit to the Scatchard model, with the fixed value of n = [bound **2×HCl**]/[polynucletide] = 0.3.

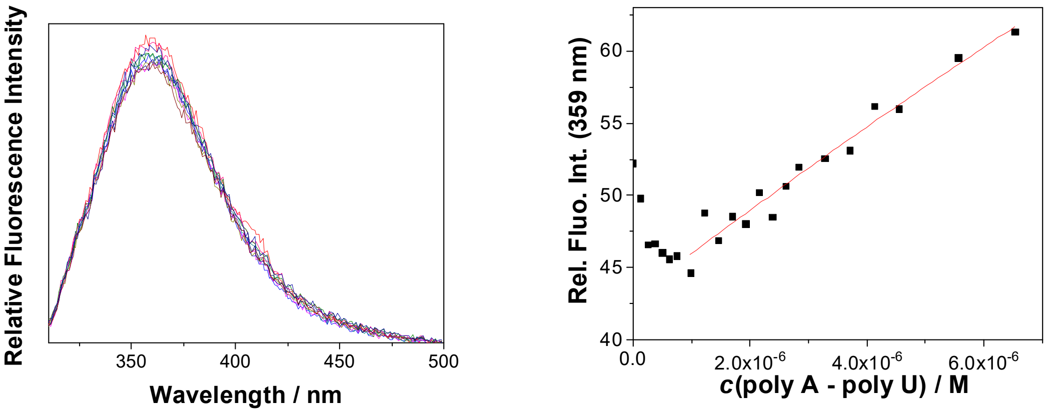


Fig. S42. LEFT: Fluorescence titration of **2×HCl** ($c = 3.0\times10^{-6}$ M, $\lambda_{exc} = 295$ nm) with poly A - poly U in cacodylate buffer (pH 7.0, 50 mM at 25 °C); RIGHT: dependence of the fluorescence intensity (normalized to starting emission) at $\lambda_{em} = 359$ nm on the *c*(polynucleotide), dots are experimental values and the lines represent calculated fit to the Scatchard model, with the fixed value of n = [bound **2×HCl**]/[polynucleotide] = 0.3.

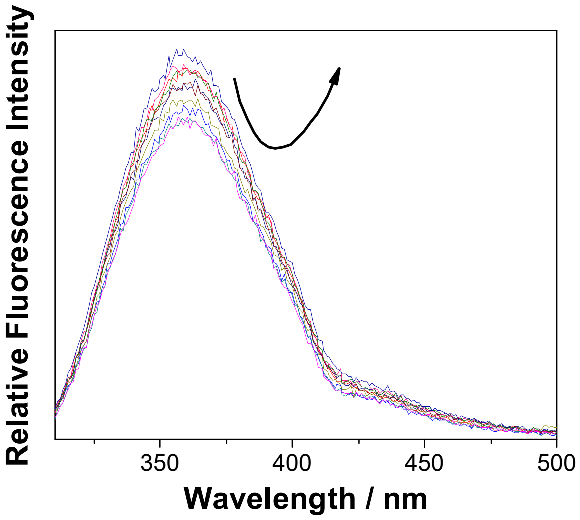


Fig. S43. Fluorescence titration of **3×HCl** ($c = 3.0\times10^{-6}$ M, $\lambda_{exc} = 295$ nm) with poly dAdT – poly dAdT in cacodylate buffer (pH 7.0, 50 mM at 25 °C); dependence of the fluorescence intensity at $\lambda_{em} = 380$ nm (normalized to starting emission) on the *c*(polynucleotide) is shown in Fig. 3.

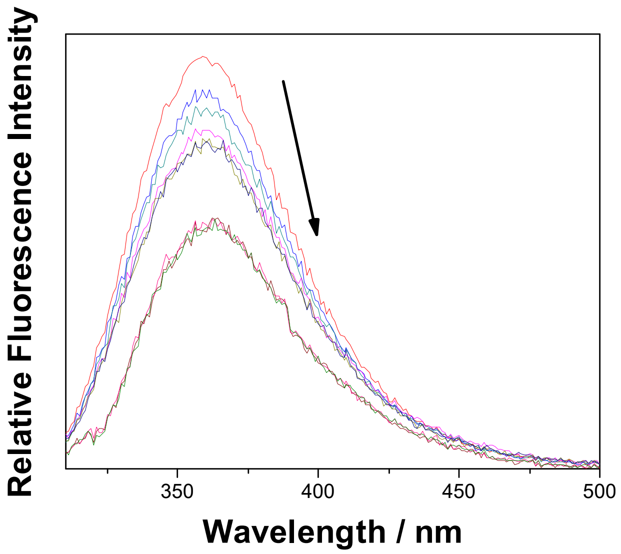


Fig. S44. Fluorescence titration of **3×HCl** ($c$ = 3.0×10$^{-6}$ M, $\lambda_{exc}$ = 295 nm) with poly dGdC – poly dGdC in cacodylate buffer (pH 7.0, 50 mM at 25 °C); dependence of the fluorescence intensity at $\lambda_{em}$ = 380 nm (normalized to starting emission) on the $c$(polynucleotide) is shown in Fig. 3.

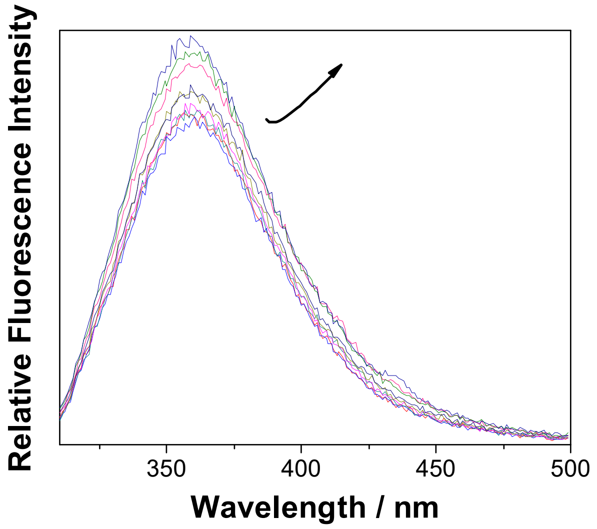


Fig. S45. Fluorescence titration of **3×HCl** ($c$ = 3.0×10$^{-6}$mol dm$^{-3}$, $\lambda_{exc}$ = 295 nm) with poly A – poly U in cacodylate buffer (pH 7.0, 50 mM at 25 °C); dependence of the fluorescence intensity at $\lambda_{em}$ = 380 nm (normalized to starting emission) on the $c$(polynucleotide) shown in Fig. 3. Done in cacodylate buffer (pH 7.0, $I$=50 mM, at 25 °C).

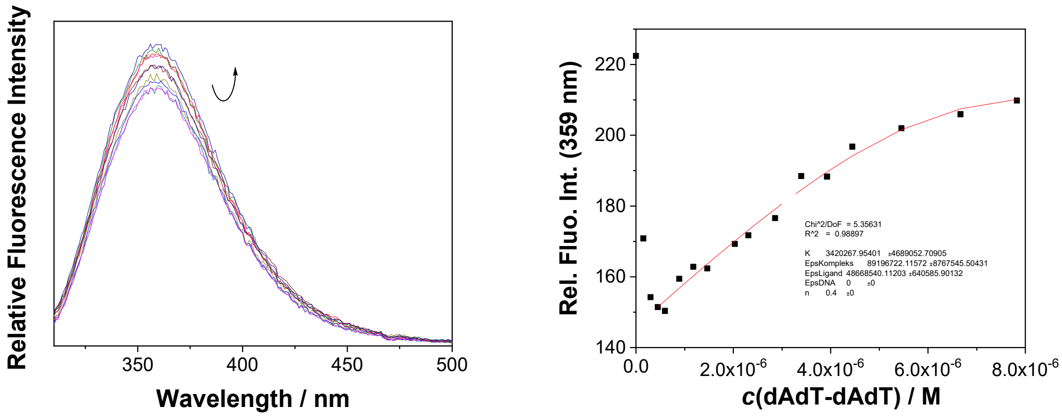


Fig. S46. Left: Fluorimetric titration of **3×HCl** ($c$ = 3.0×10$^{-6}$ M, $\lambda_{exc}$ = 280 nm) with poly dAdT - poly dAdT ($c$ = 1.2×10$^{-6}$ - 6.7×10$^{-5}$ M); in cacodylate buffer (pH 7.0, 50 mM, at 25 °C). Right: Dependence of the fluorescence intensity at 359 nm on the polynucleotide concentration; dots are experimental values and the red line is calculated fit according to the Scatchard model, with the fixed value of $n$ = [**3×HCl**]/[dAdT] = 0.4.

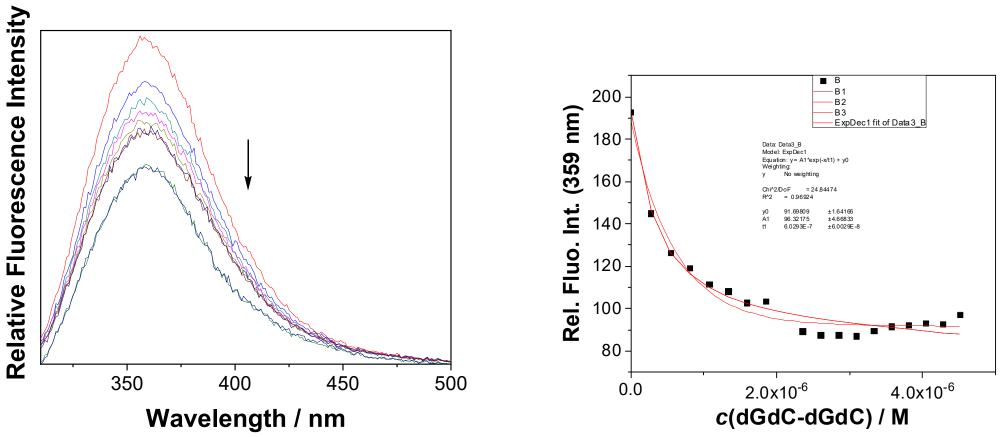


Fig. S47. Left: Fluorimetric titration of **3×HCl** ($c$ = 3.0×10$^{-6}$ M, $\lambda_{exc}$ = 280 nm) with poly dGdC - poly dGdC ($c$ = 1.2×10$^{-6}$ - 6.7×10$^{-5}$ M); in cacodylate buffer (pH 7.0, 50 mM, at 25 °C). Right: Dependence of the fluorescence intensity at 359 nm on the polynucleotide concentration; dots are experimental values and the red line is calculated fit according to the Scatchard model. Data could not be fit to the model with the fixed value of $n$ = [**3×HCl**]/[dAdT] < 1.

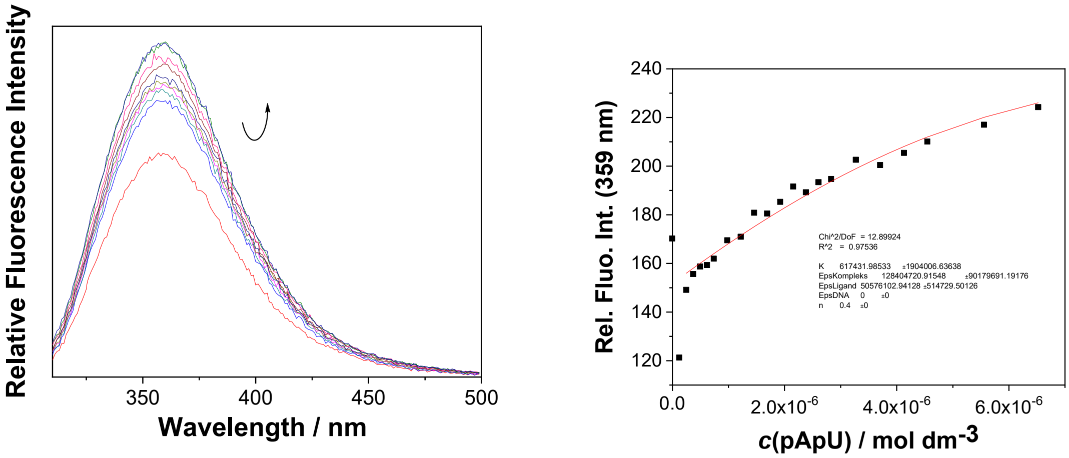


Fig. S48. Left: Fluorimetric titration of **3×HCl** ($c$ = 3.0×10$^{-6}$ M, $\lambda_{exc}$ = 280 nm) with polyA-poly U ($c$ = 1.2×10$^{-6}$ - 6.7×10$^{-5}$ M); in cacodylate buffer (pH 7.0, 50 mM, at 25 °C). Right: Dependence of the fluorescence intensity at 359 nm on the poly A – poly U concentration; dots are experimental values and the red line is calculated fit according to the Scatchard model with the fixed value of $n$ = [**3×HCl**]/[poly A – poly U] = 0.4.

The effect to the polynucleotide melting temperature is defined as:

$$\Delta T_m = T_m\,(\text{complex}) - T_m\,(\text{ct-DNA}) \qquad \text{(S8)}$$

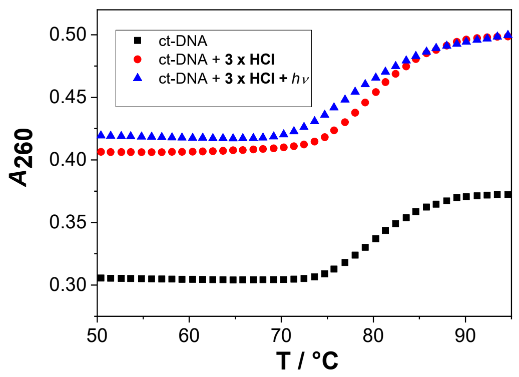


Fig. S49. Thermal denaturation of ct-DNA ($c = 3\times10^{-5}$ M), in the presence of **3×HCl** at ratio *r*[**3×HCl**] / [polynucleotide]=0.3, at pH = 7.0, cacodylate buffer (50 mM), and after irradiation of ct-DNA and 3 (300 nm, 5 min).

Table S3. The $\Delta T_m$ values[a] of ct-DNA upon the addition of **3×HCl** at pH 7.0 (sodium cacodylate buffer, $I = 0.05$ M) and upon irradiation (300 nm, 8 lamps, 5 min).

| Compound/condition | $\Delta T_m$ / °C |
|---|---|
| **3×HCl** (dark) | 0.5 |
| **3×HCl** + h*ν* | -2.2 |

[a] Error in $\Delta T_m$ : ± 0.5°C; The melting temperature was determined from the inflection point of the dependence of absorbance on temperature.[6] *r*[**3×HCl**] / [ct-DNA]= 0.3.

CD experiments

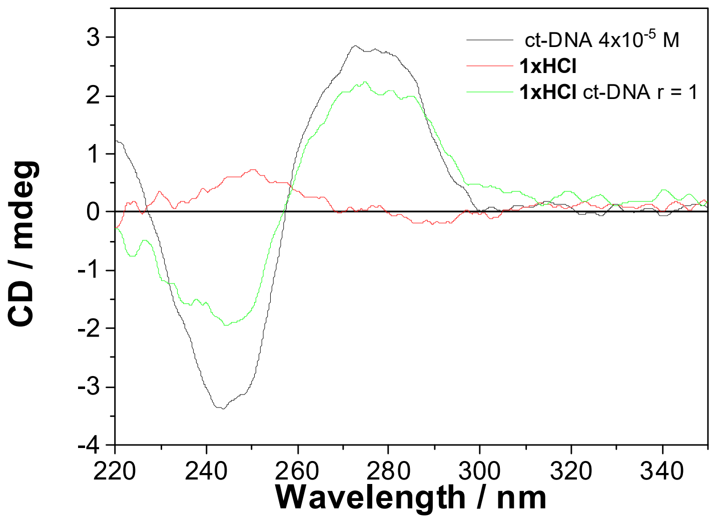


Fig. S50. CD spectra of ct-DNA ($c$ = 4.0×10$^{-5}$ M), **1×HCl** and their mixture at r[**1×HCl**]/[ct-DNA] = 1. The measurement was performed in cacodylate buffer / 20% DMSO (pH 7.0, 50 mM, at 25 °C).

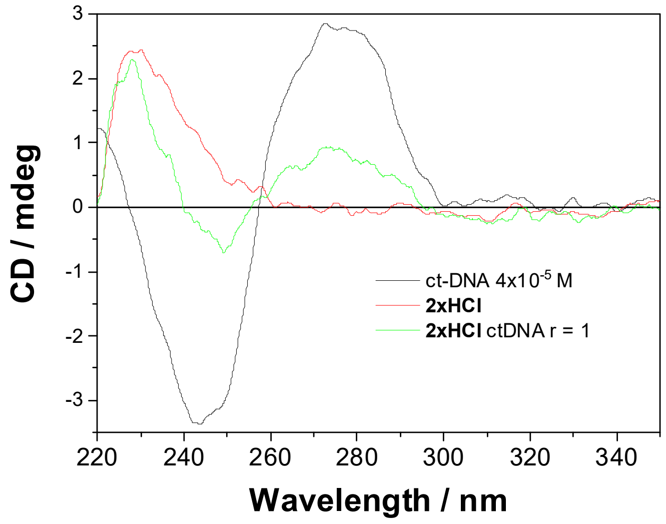


Fig. S51. CD spectra of ct-DNA ($c$ = 4.0×10$^{-5}$ M), **2×HCl** and their mixture at r[**2×HCl**]/[ct-DNA] = 1. The measurement was performed in cacodylate buffer / 20% DMSO (pH 7.0, 50 mM, at 25 °C).

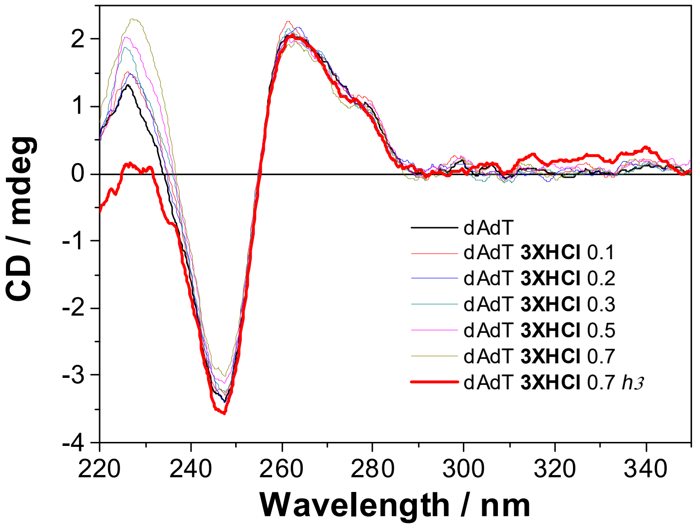


Fig. S52. CD spectra of poly dAdT – poly dAdT ($c$ = 2.0×10$^{-5}$ M) in the presence of different ratios r[**3×HCl**]/[AT-DNA]. The measurement was performed in cacodylate buffer / 20% DMSO (pH 7.0, 50 mM, at 25 °C).

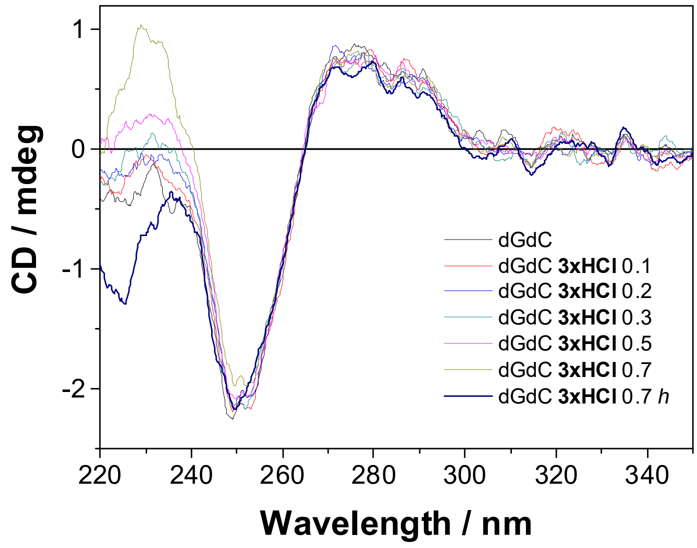


Fig. S53. CD spectra of poly dGdC – poly dGdC ($c$ = 2.0×10$^{-5}$ M) in the presence of different ratios r[**3×HCl**]/[GC-DNA]. The measurement was performed in cacodylate buffer (pH 7.0, 50 mM, at 25 °C).

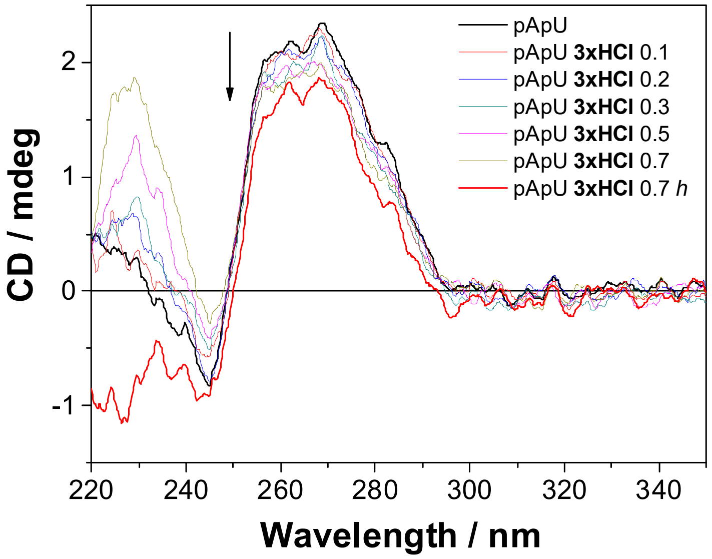


Fig. S54. CD spectra of poly A – poly U ($c$ = 2.0×10$^{-5}$ M) in the presence of different ratios r[**3×HCl**]/[AU-RNA]. The measurement was performed in cacodylate buffer (pH 7.0, 50 mM, at 25 °C).

## 5. Covalent binding to oligonucleotides

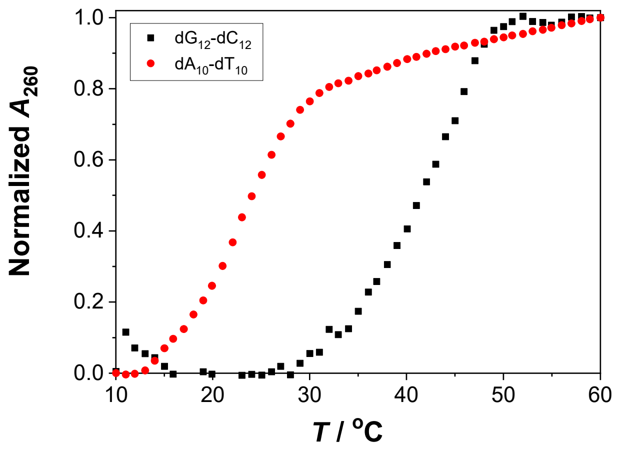


Fig. S55. Thermal denaturation of dG12-dC12 and dA10-dT10 ($c$ = 2.0 × $10^{-5}$ M) in ammonium acetate buffer (pH = 7.0, 100 mM). The measured $T_m$ for dG12-dC12 is 40 °C, and for dA10-dT10 is 22 °C.

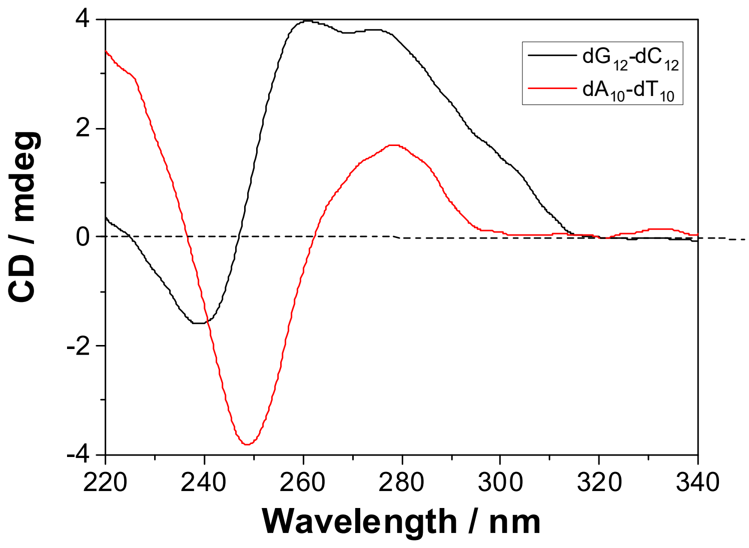


Fig. S56. CD spectra of dG12-dC12 and dA10-dT10 ($c$ = 2.0 × $10^{-5}$ M) in ammonium acetate buffer (pH = 7.0, 100 mM) at 25 °C.

Analyses of the solutions containing annealed oligonucleotides and **3×HCl** were performed on a Shimadzu HPLC equipped with a dyode array detector and a PLRP-S 5 μm column. The parameters for the chromatography method are given in Table S4.

Table S4. Chromatography method for the analysis of oligonucleotides and **3×HCl**.

| *t* / min | B (%) |
|---|---|
| 0 | 5 |
| 5 | 5 |
| 15 | 40 |
| 20 | 100 |
| 25 | 100 |
| 32 | 5 |
| 40 | 5 |

A: $NH_4Ac$ (100 mM, pH 7.0)
B: ACN
Flow rate: 0.5 mL/ min.

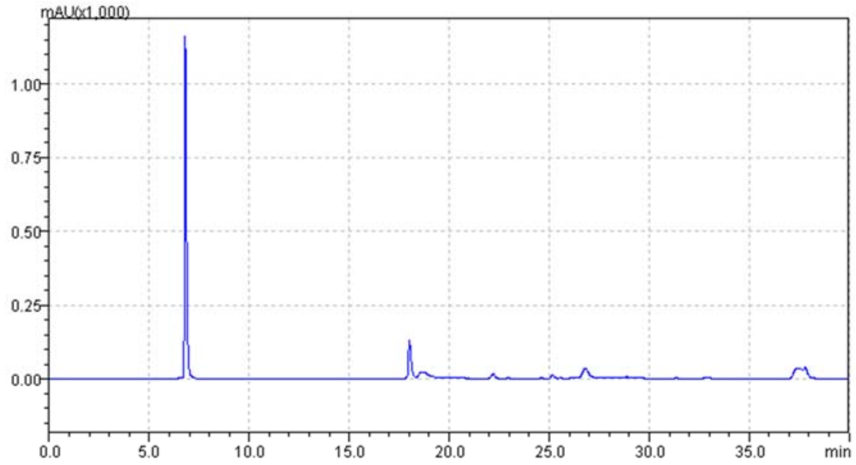


Fig. S57. HPLC chromatogram of $dG_{12}$-$dC_{12}$.

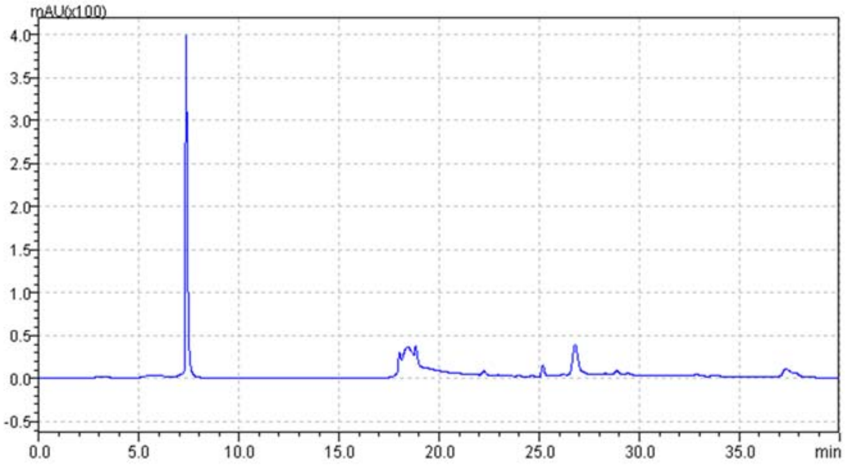


Fig. S58. HPLC chromatogram of dG12-dC12 after 15 min irradiation at 300 nm (8 lamps).

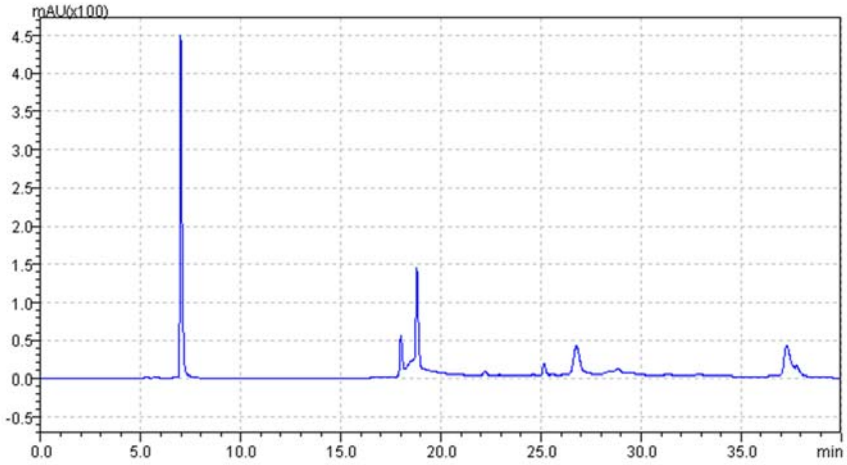


Fig. S59. HPLC chromatogram of dG12-dC12 and **3×HCl** before the irradiation.

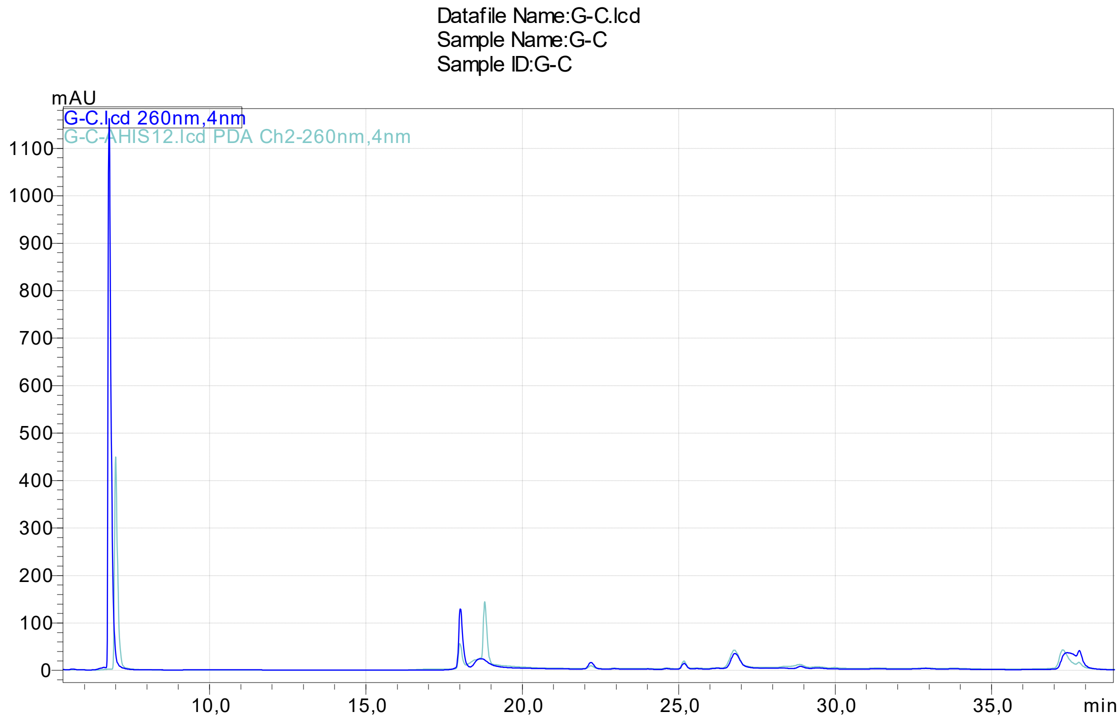


Fig. S60. Overlapped HPLC chromatogram of dG12-dC12 and **3×HCl** before the irradiation.

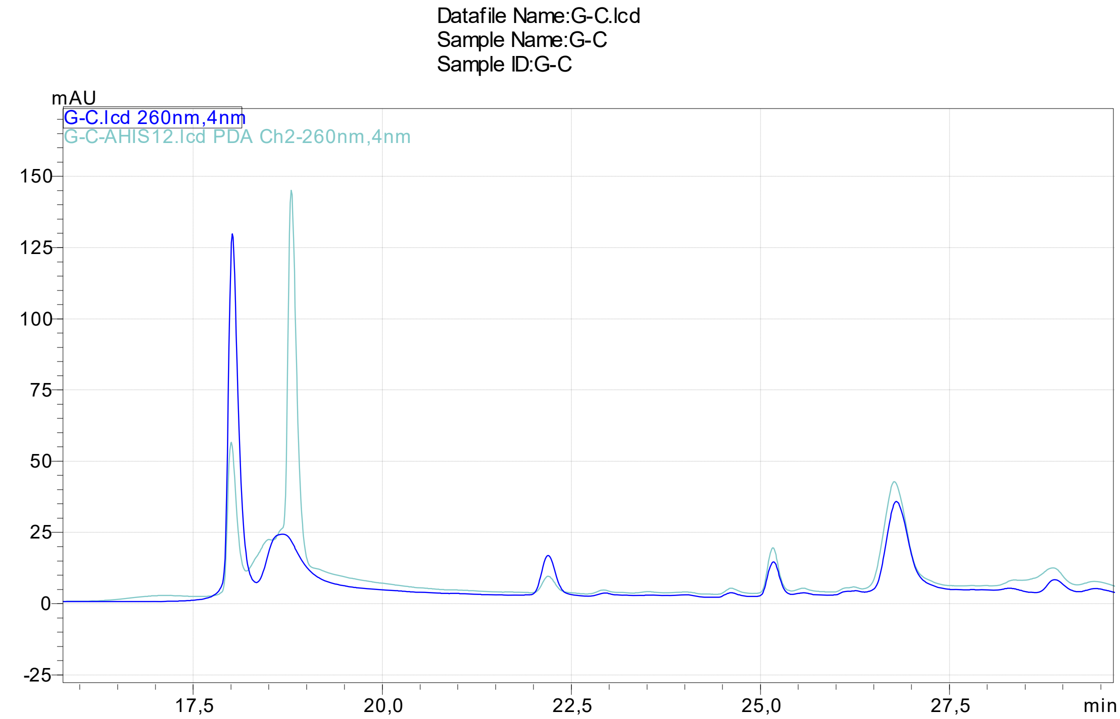


Fig. S61. Overlapped parts of HPLC chromatogram of dG12-dC12 and **3×HCl** before the irradiation.

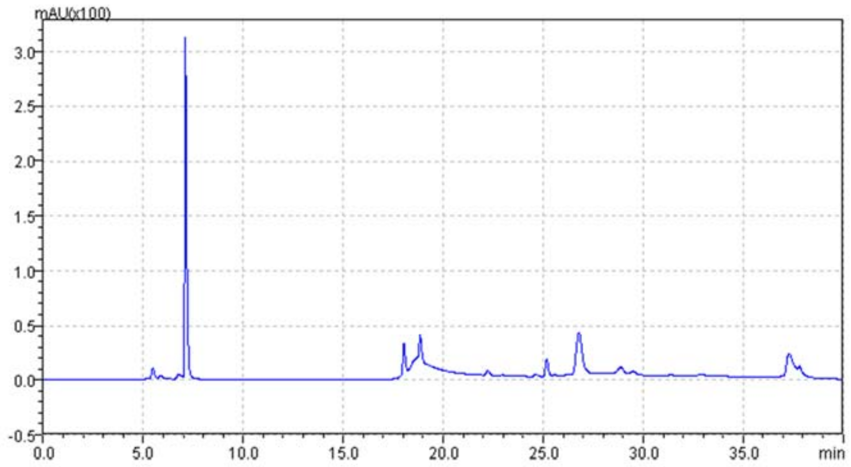


Fig. S62. HPLC chromatogram of dG12-dC12 and **3×HCl** after 1 h irradiation at 300 nm (8 lamps).

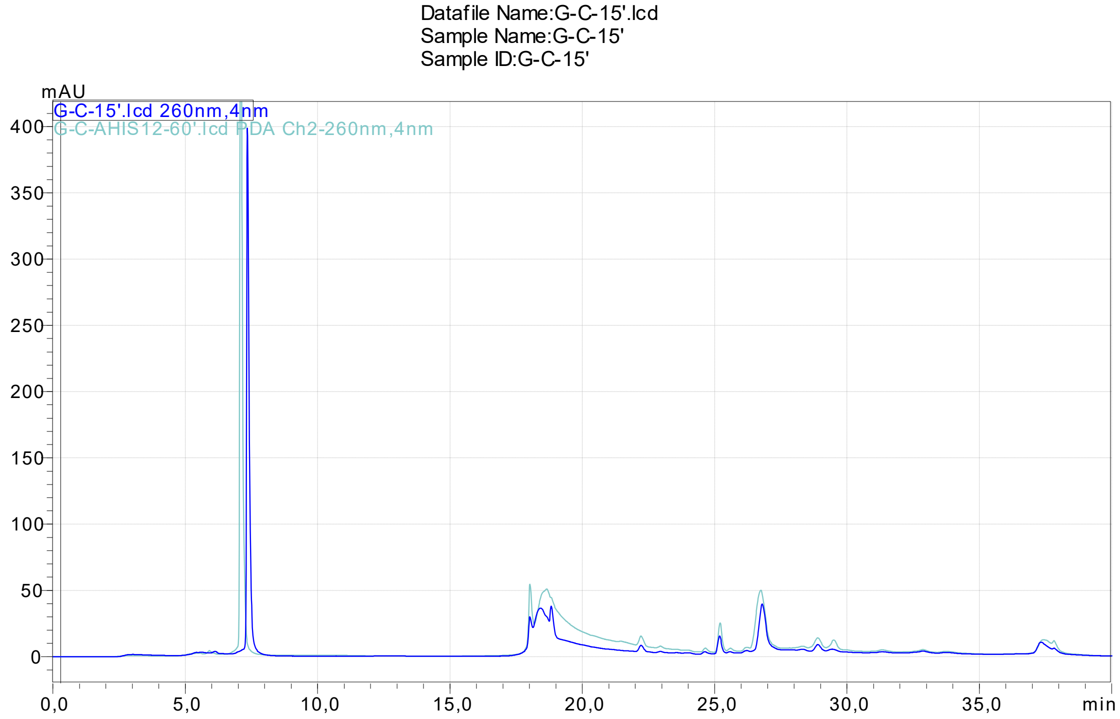


Fig. S63. Overlapped HPLC chromatogram of dG12-dC12 and **3×HCl** after 1 h irradiation at 300 nm (8 lamps).

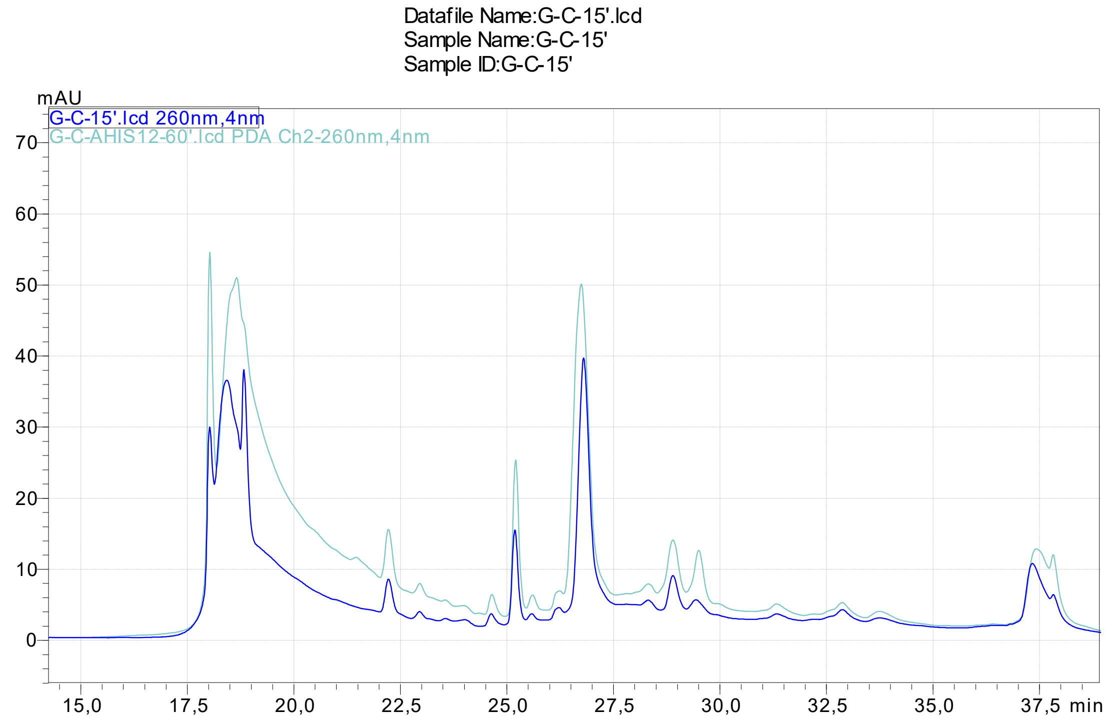


Fig. S64. Overlapped parts of HPLC chromatogram of dG12-dC12 and **3×HCl** after 1 h irradiation at 300 nm (8 lamps).

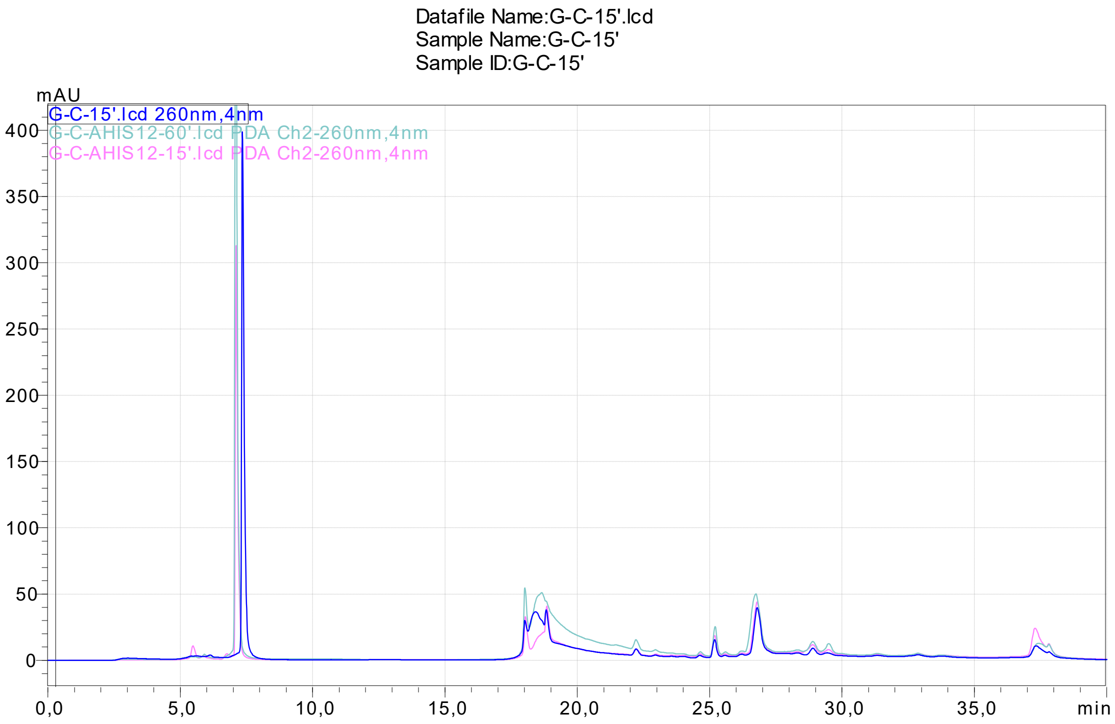


Fig. S65. Overlapped HPLC chromatogram of dG12-dC12 and **3×HCl** after 1 h irradiation at 300 nm (8 lamps).

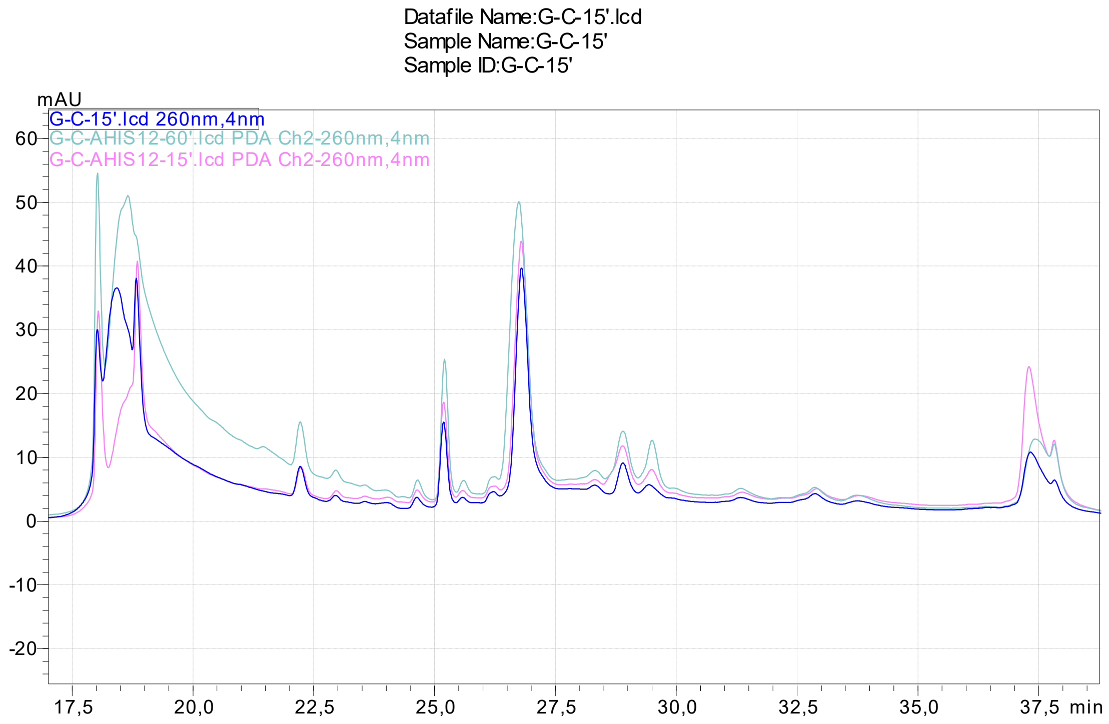


Fig. S66. Overlapped parts of HPLC chromatogram of dG$_{12}$-dC$_{12}$ and **3×HCl** after 1 h irradiation at 300 nm (8 lamps).

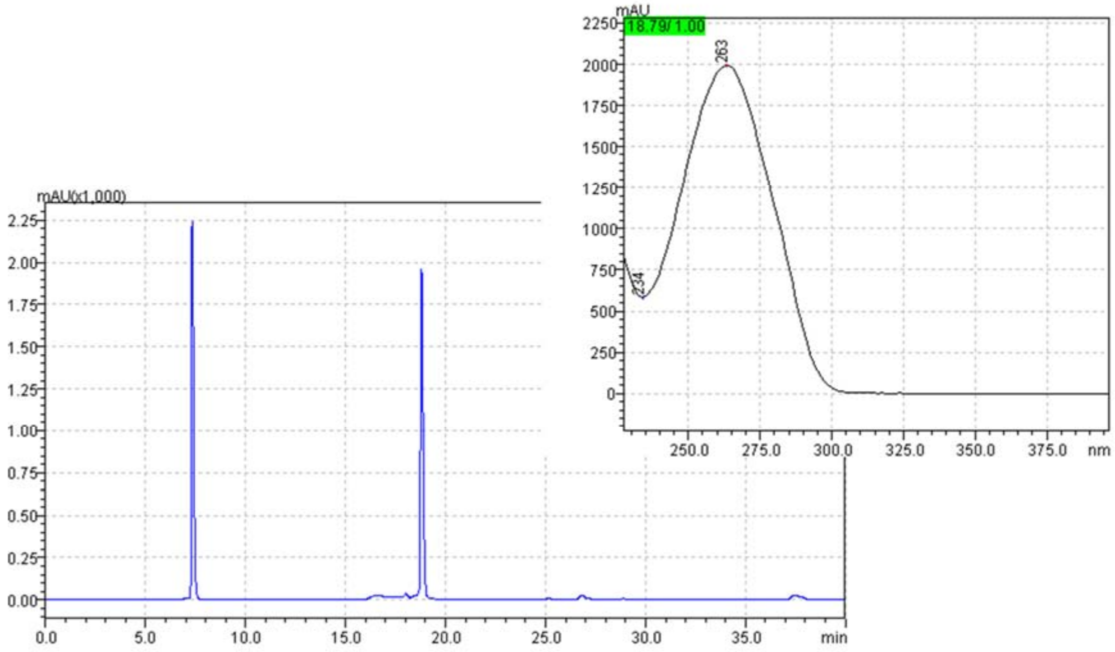


Fig. S67. HPLC chromatogram of dA$_{10}$-dT$_{10}$ (inset: absorption spectrum of the species with rt 18.8 min).

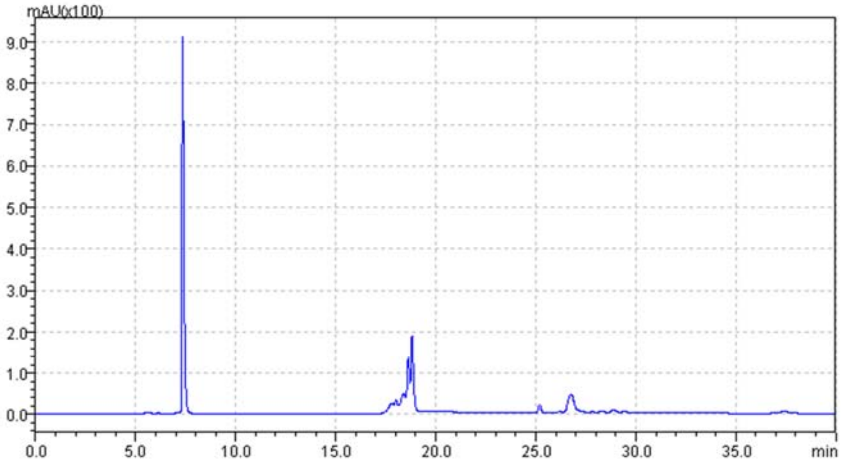


Fig. S68. HPLC chromatogram of $dA_{10}$-$dT_{10}$ after 15 min irradiation at 300 nm (8 lamps).

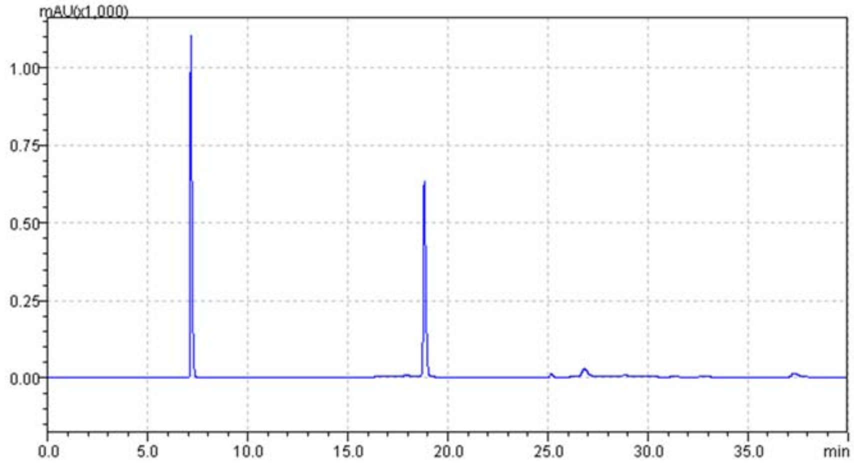


Fig. S69. HPLC chromatogram of $dA_{10}$-$dT_{10}$ and **3×HCl** before the irradiation.

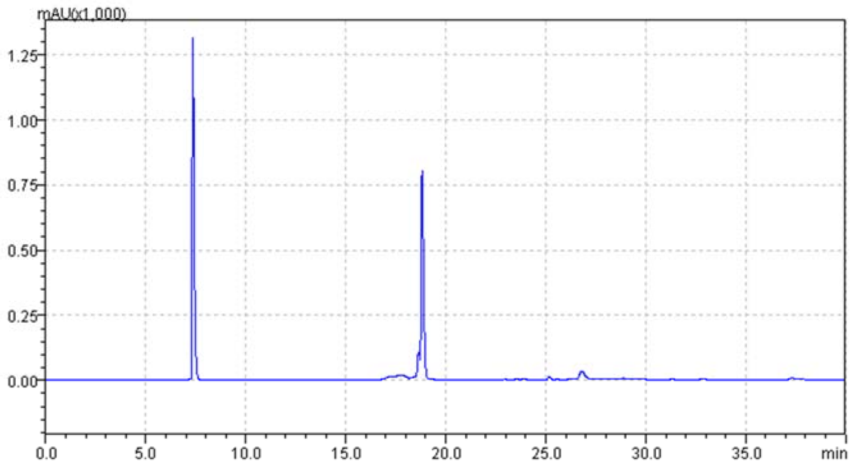


Fig. S70. HPLC chromatogram of $dA_{10}$-$dT_{10}$ and **3×HCl** after 1 h irradiation at 300 nm (8 lamps).

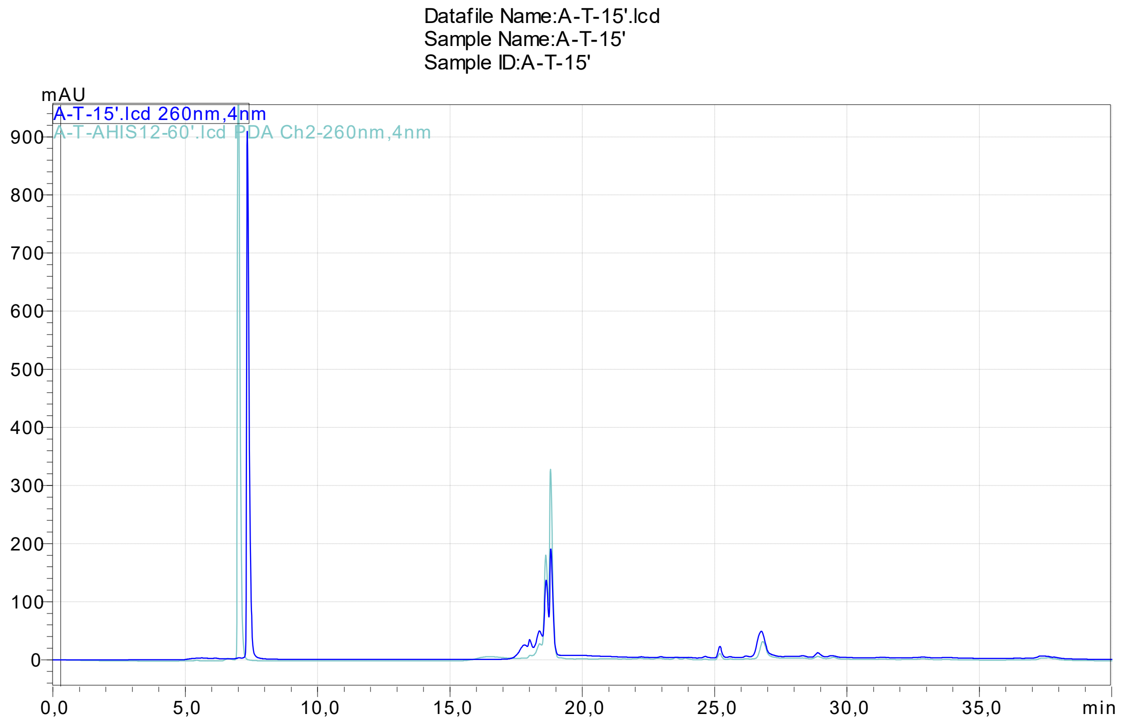


Fig. S71. Overlapped HPLC chromatogram of $dA_{10}$-$dT_{10}$ and **3×HCl** after 1 h irradiation at 300 nm (8 lamps).

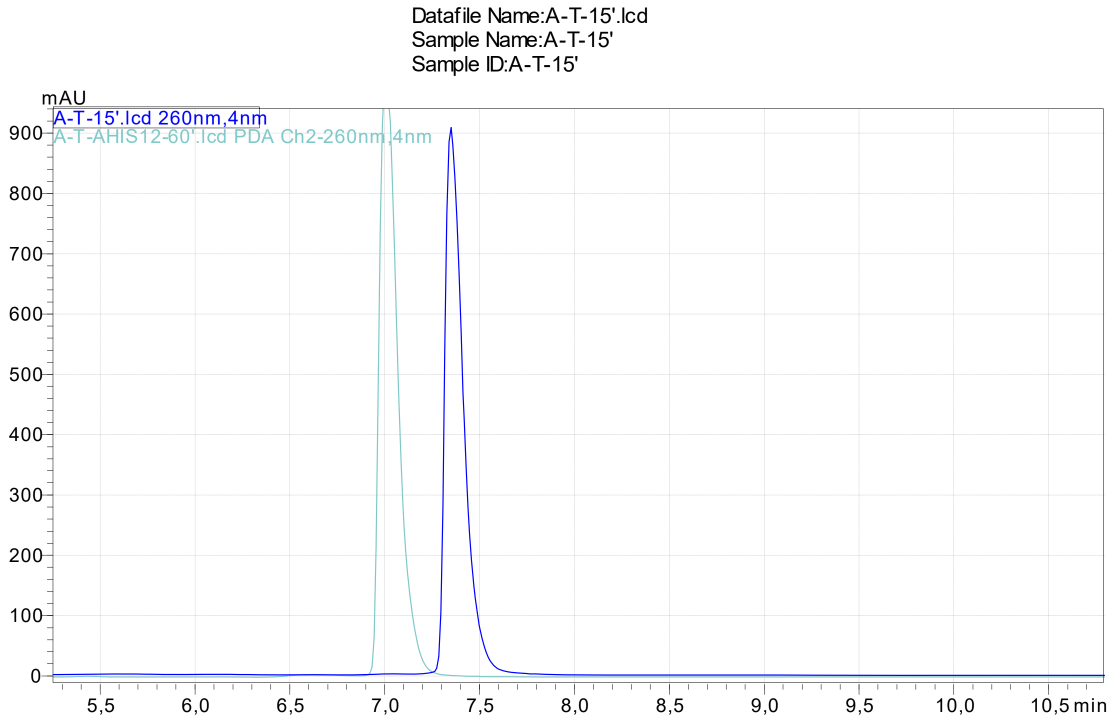


Fig. S72. Overlapped parts of HPLC chromatogram of dA$_{10}$-dT$_{10}$ and **3×HCl** after 1 h irradiation at 300 nm (8 lamps).

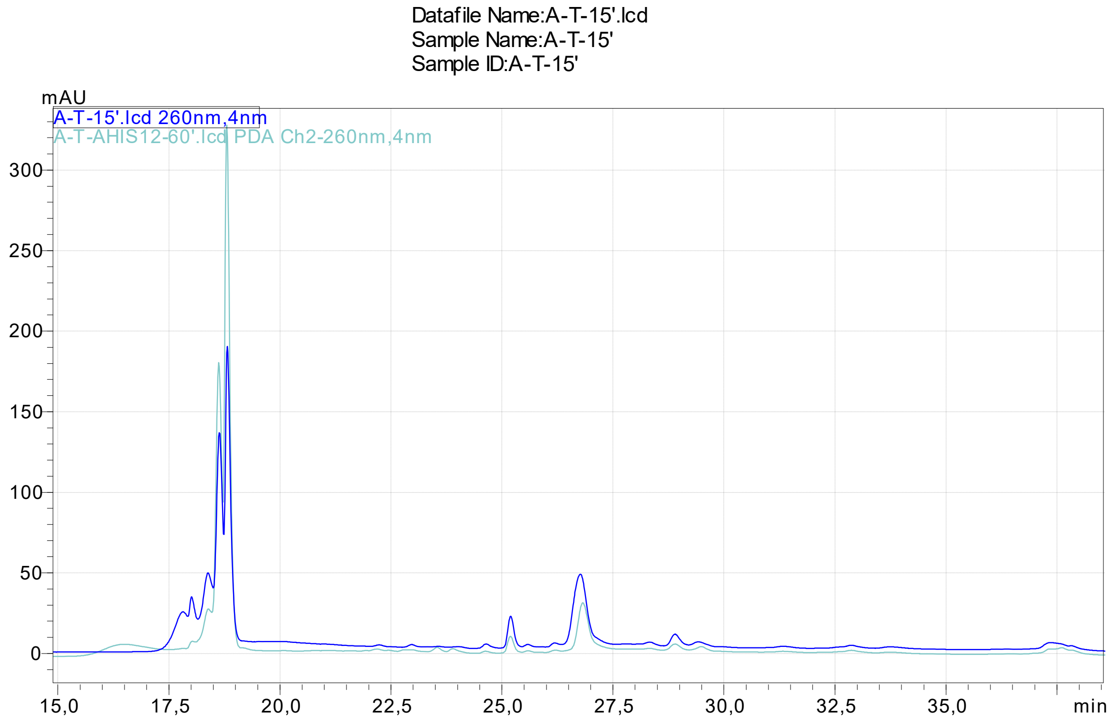


Fig. S73. Overlapped parts of HPLC chromatogram of dA$_{10}$-dT$_{10}$ and **3×HCl** after 1 h irradiation at 300 nm (8 lamps).

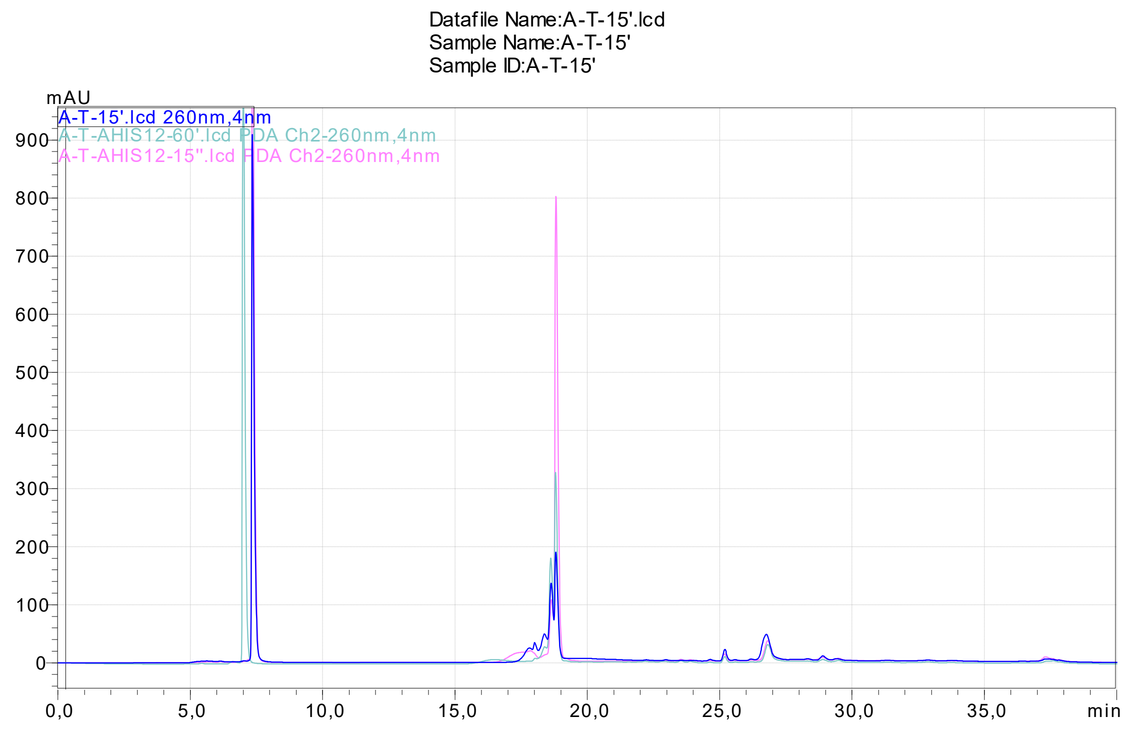


Fig. S74. Overlapped HPLC chromatogram of dA10-dT10 and **3×HCl** after 15 min and 1 h irradiation at 300 nm (8 lamps).

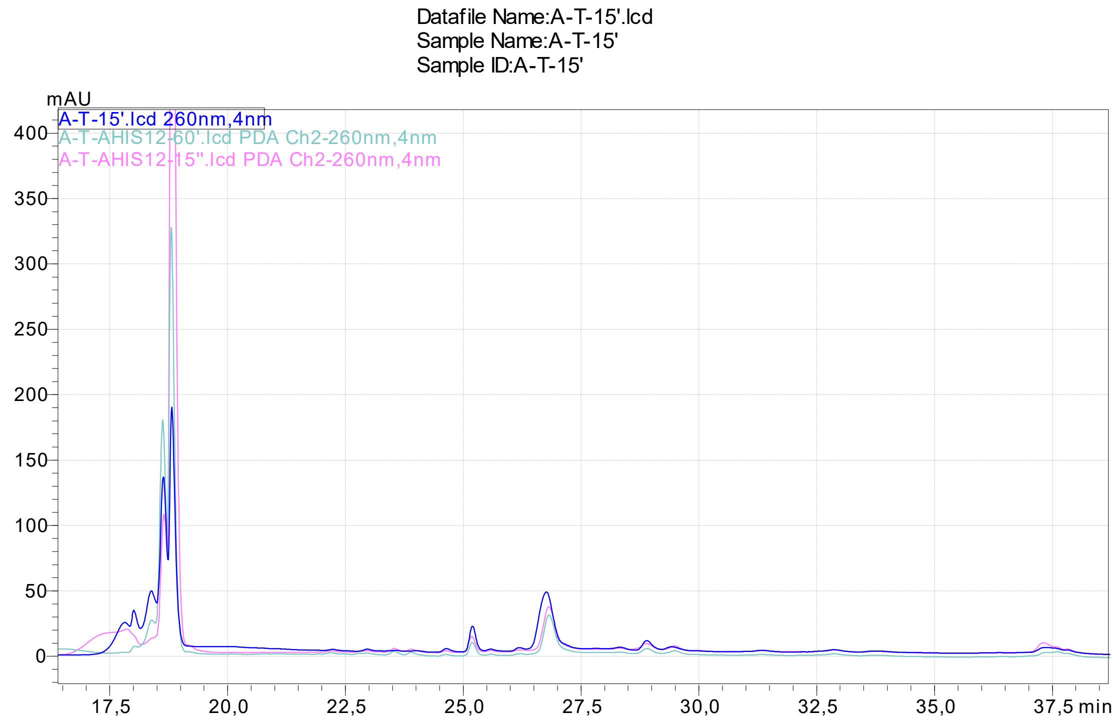


Fig. S75. Overlapped parts of HPLC chromatogram of dA10-dT10 and **3×HCl** after 15 min and 1 h irradiation at 300 nm (8 lamps).

## 5. NMR spectra

$^{1}$H NMR ($CD_3OD$, 300 MHz) of ***N*-Boc-L-Trp-OSu**

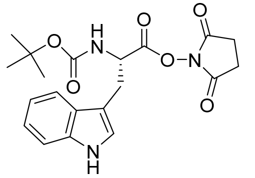

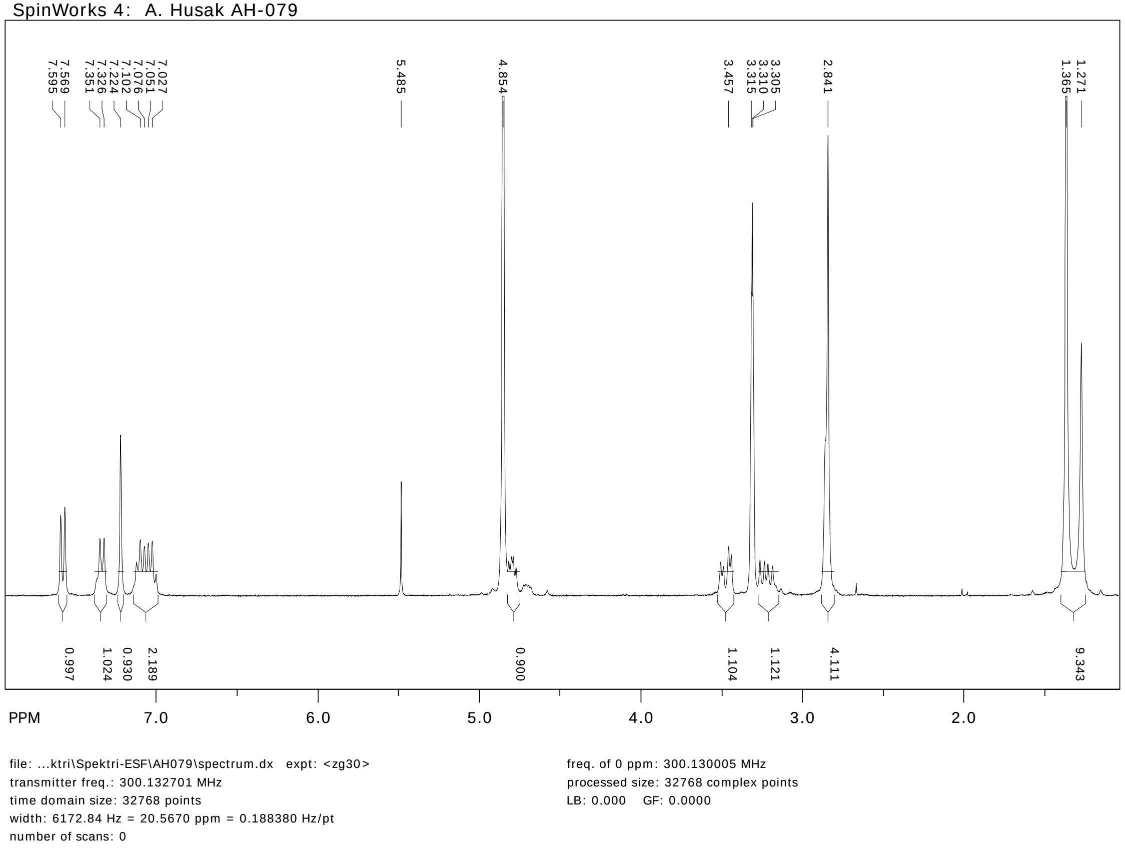

$^{1}H$ NMR ($CD_3OD$, 300 MHz) of ***N*-Boc-L-Trp-L-Trp-OH**

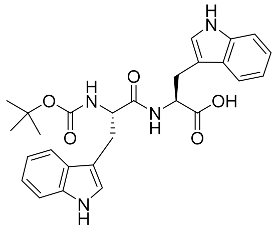

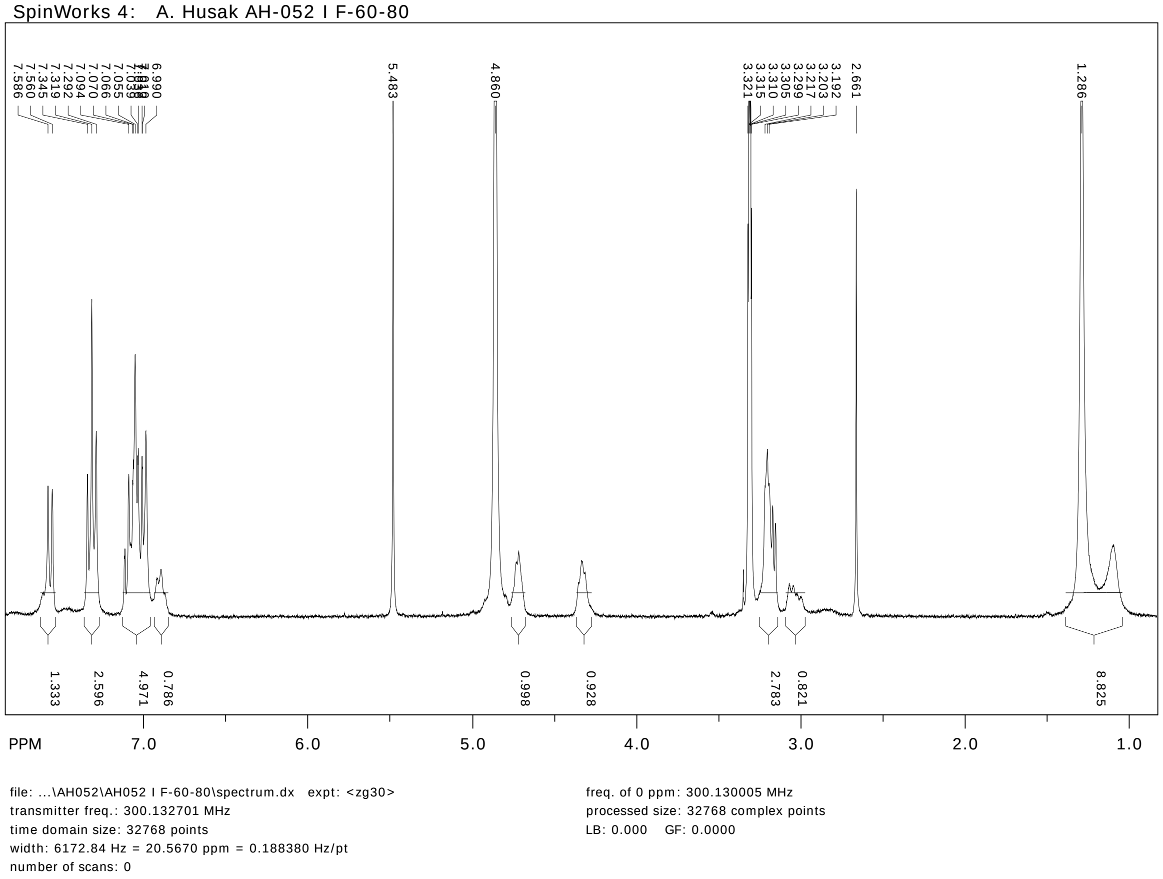

$^{1}$H NMR (CD$_3$OD, 300 MHz) of ***N*-Boc-L-Trp-L-Trp-OSu**

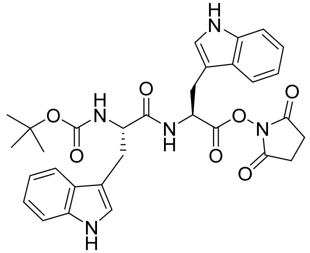

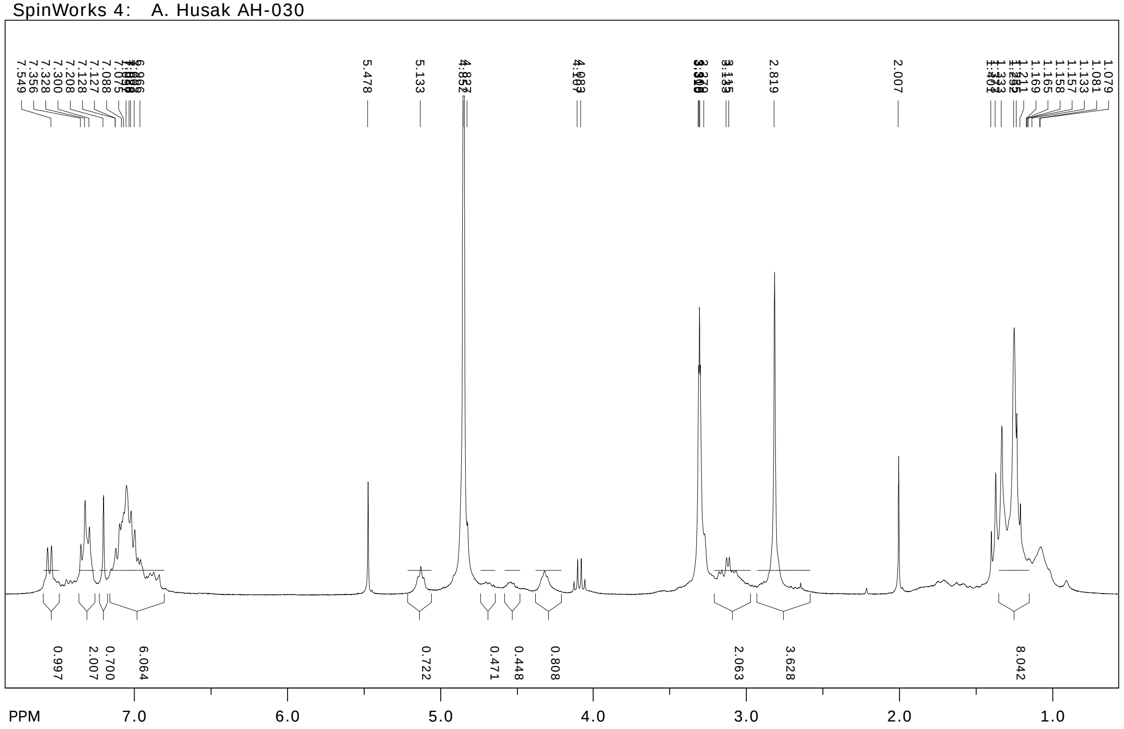


file: ...ktri\Spektri-ESF\AH030\spectrum.dx expt: <zg30>
transmitter freq.: 300.132701 MHz
time domain size: 32768 points
width: 6172.84 Hz = 20.5670 ppm = 0.188380 Hz/pt
number of scans: 0

freq. of 0 ppm: 300.130006 MHz
processed size: 32768 complex points
LB: 0.000 GF: 0.0000

$^{1}$H NMR ($CD_3OD$, 300 MHz) of **Boc-L-Trp-L-Trp-L-Phe-OBn (1)**

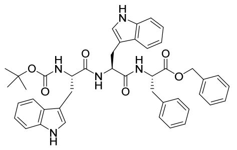


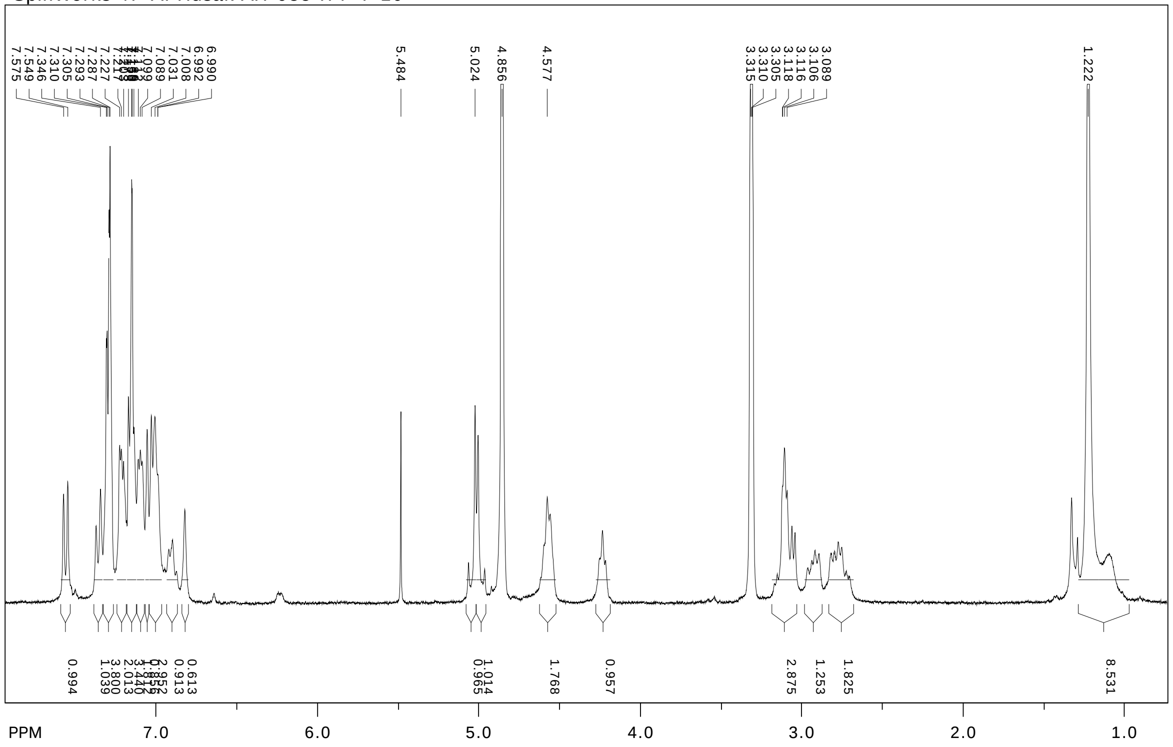


file: ...\AH085\AH085 II F-7-10\spectrum.dx expt: <zg30>
transmitter freq.: 300.132701 MHz
time domain size: 32768 points
width: 6172.84 Hz = 20.5670 ppm = 0.188380 Hz/pt
number of scans: 0

freq. of 0 ppm: 300.130005 MHz
processed size: 32768 complex points
LB: 0.000 GF: 0.0000

$^{1}$H NMR ($CD_3OD$, 300 MHz) of **HCl×H-L-Trp-L-Trp-L-Phe-OBn (1×HCl)**

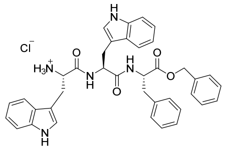

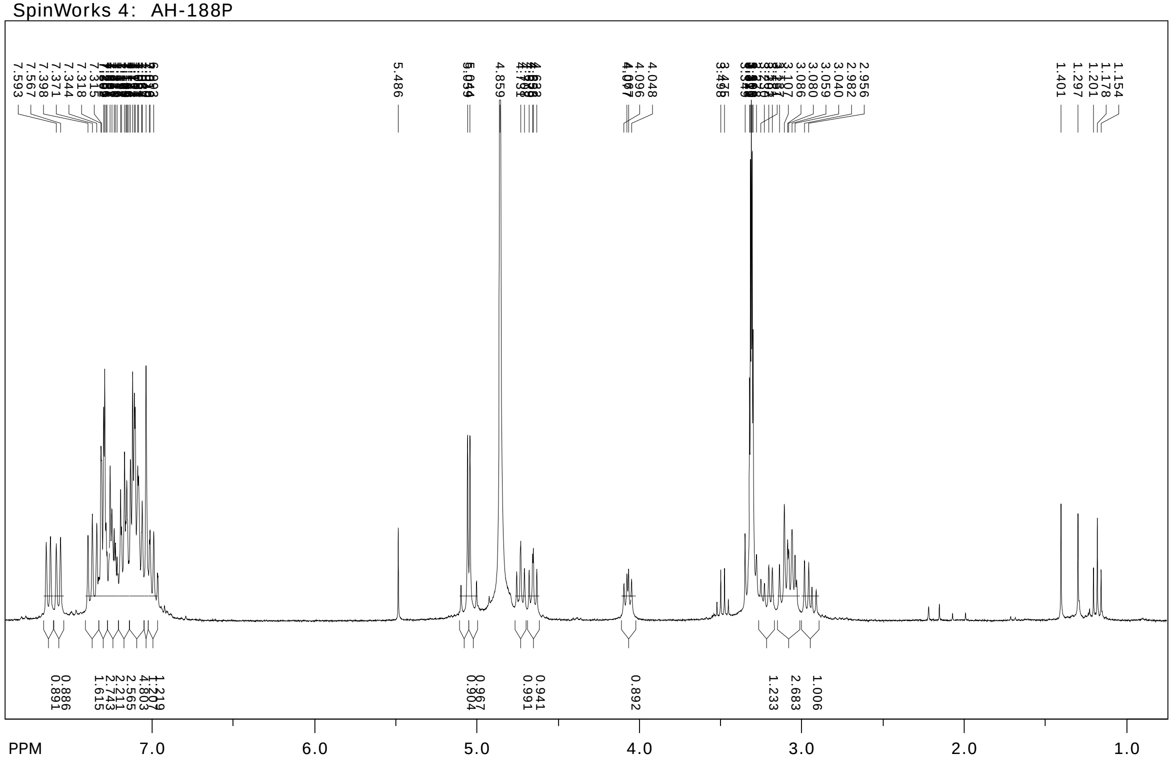


file: ...Desktop\husak_ah188p\1\spectrum.dx expt: <zg30>
transmitter freq.: 300.132701 MHz
time domain size: 32768 points
width: 6172.84 Hz = 20.5670 ppm = 0.188380 Hz/pt
number of scans: 0

freq. of 0 ppm: 300.130005 MHz
processed size: 32768 complex points
LB: 0.000 GF: 0.0000

$^{1}$H NMR ($CD_3OD$, 300 MHz) of **Boc-L-Trp-L-Trp-L-Tyr-OBn (2)**

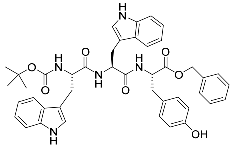


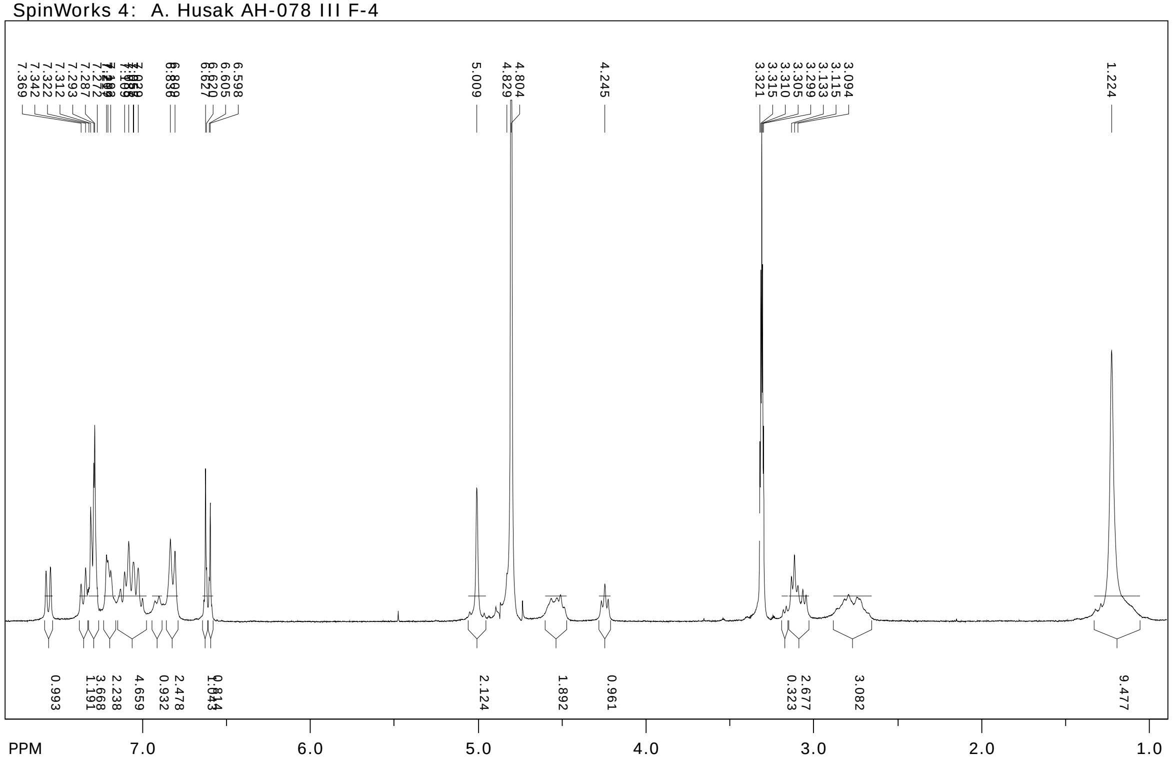


file: ...-spektri\AH078 III F-4\spectrum.dx expt: <zg30>
transmitter freq.: 300.132701 MHz
time domain size: 32768 points
width: 6172.84 Hz = 20.5670 ppm = 0.188380 Hz/pt
number of scans: 0

freq. of 0 ppm: 300.130005 MHz
processed size: 32768 complex points
LB: 0.000 GF: 0.0000

$^{1}$H NMR ($CD_3OD$, 300 MHz) of **HCl×H-L-Trp-L-Trp-L-Tyr-OBn (2×HCl)**

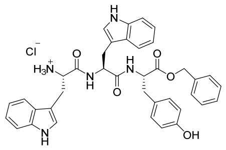


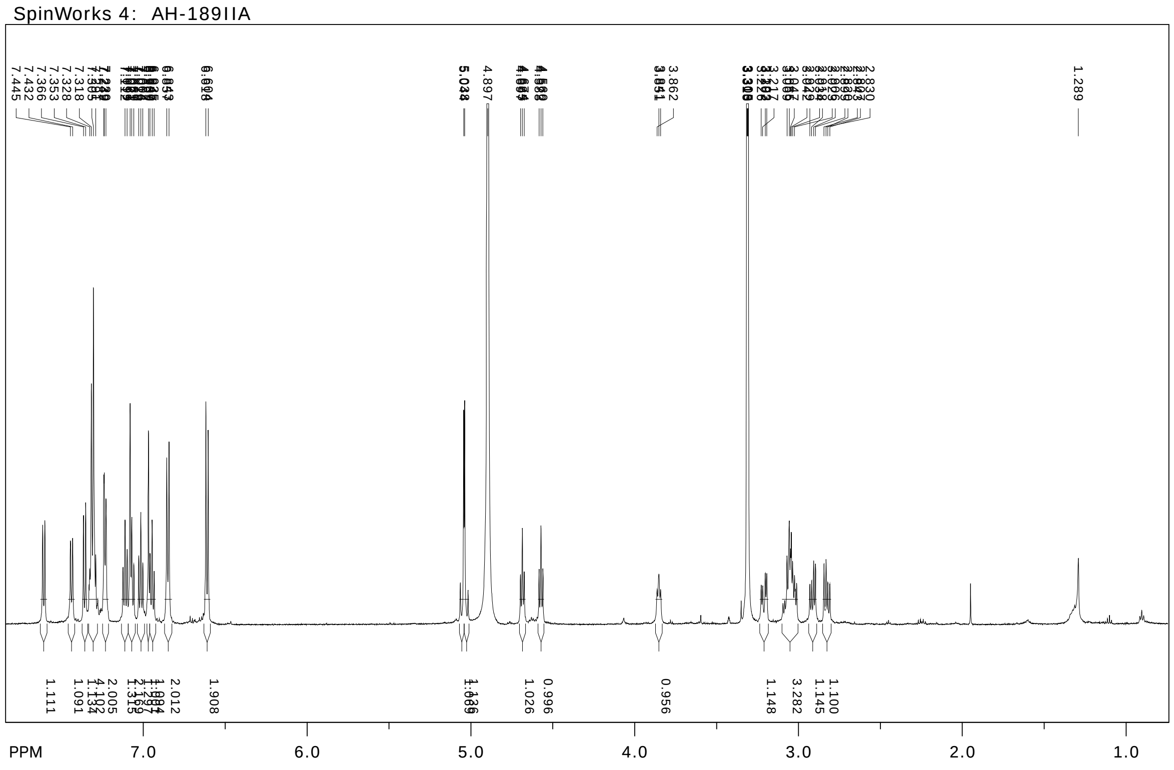


file: ...sktop\husak_ah189iia\1\spectrum.dx expt: <zg30>
transmitter freq.: 600.135401 MHz
time domain size: 32768 points
width: 12019.23 Hz = 20.0275 ppm = 0.366798 Hz/pt
number of scans: 0

freq. of 0 ppm: 600.130007 MHz
processed size: 32768 complex points
LB: 0.000 GF: 0.0000

# $^{1}$H NMR ($CD_3OD$, 300 MHz) of ***N*-Boc-L-Tyr[CH$_2$N(CH$_3$)$_2$]-OBn**

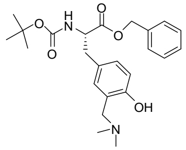

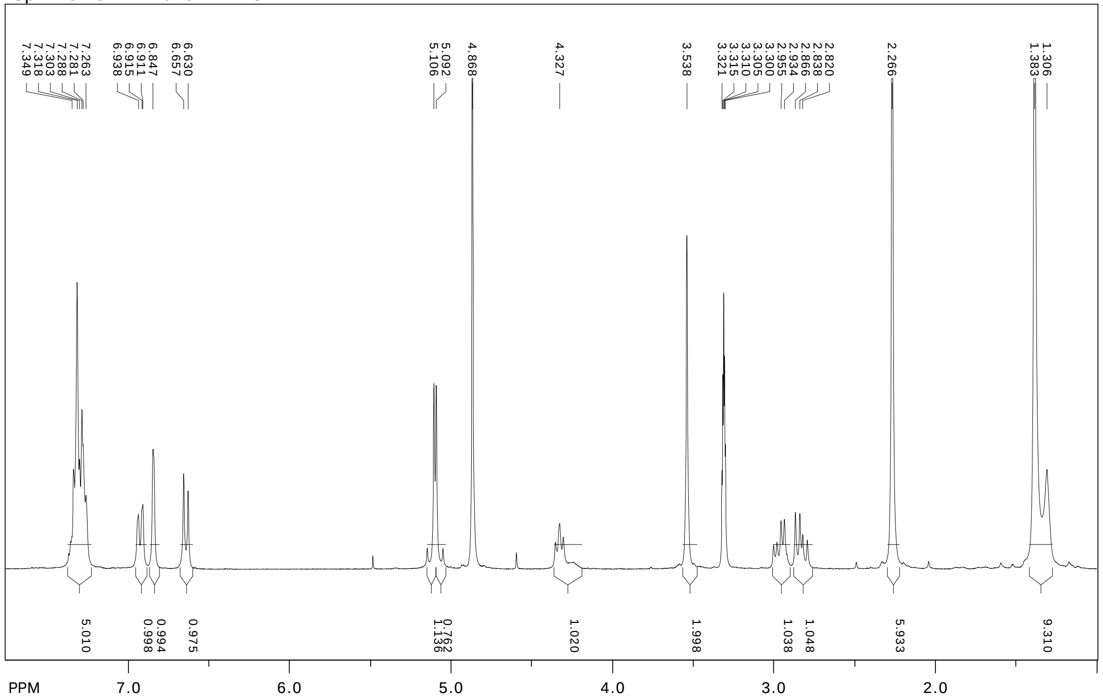


file: ...teza\BocTyr(Man)OBn\1H\spectrum.dx expt: <zg30>
transmitter freq.: 300.132701 MHz
time domain size: 32768 points
width: 6172.84 Hz = 20.5670 ppm = 0.188380 Hz/pt
number of scans: 0

freq. of 0 ppm: 300.130005 MHz
processed size: 32768 complex points
LB: 0.000 GF: 0.0000

$^{1}$H NMR ($CD_3OD$, 300 MHz) of **TFA×H-Tyr[$CH_2N(CH_3)_2$×TFA]-OBn**

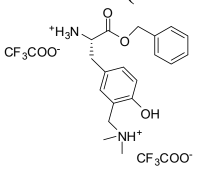


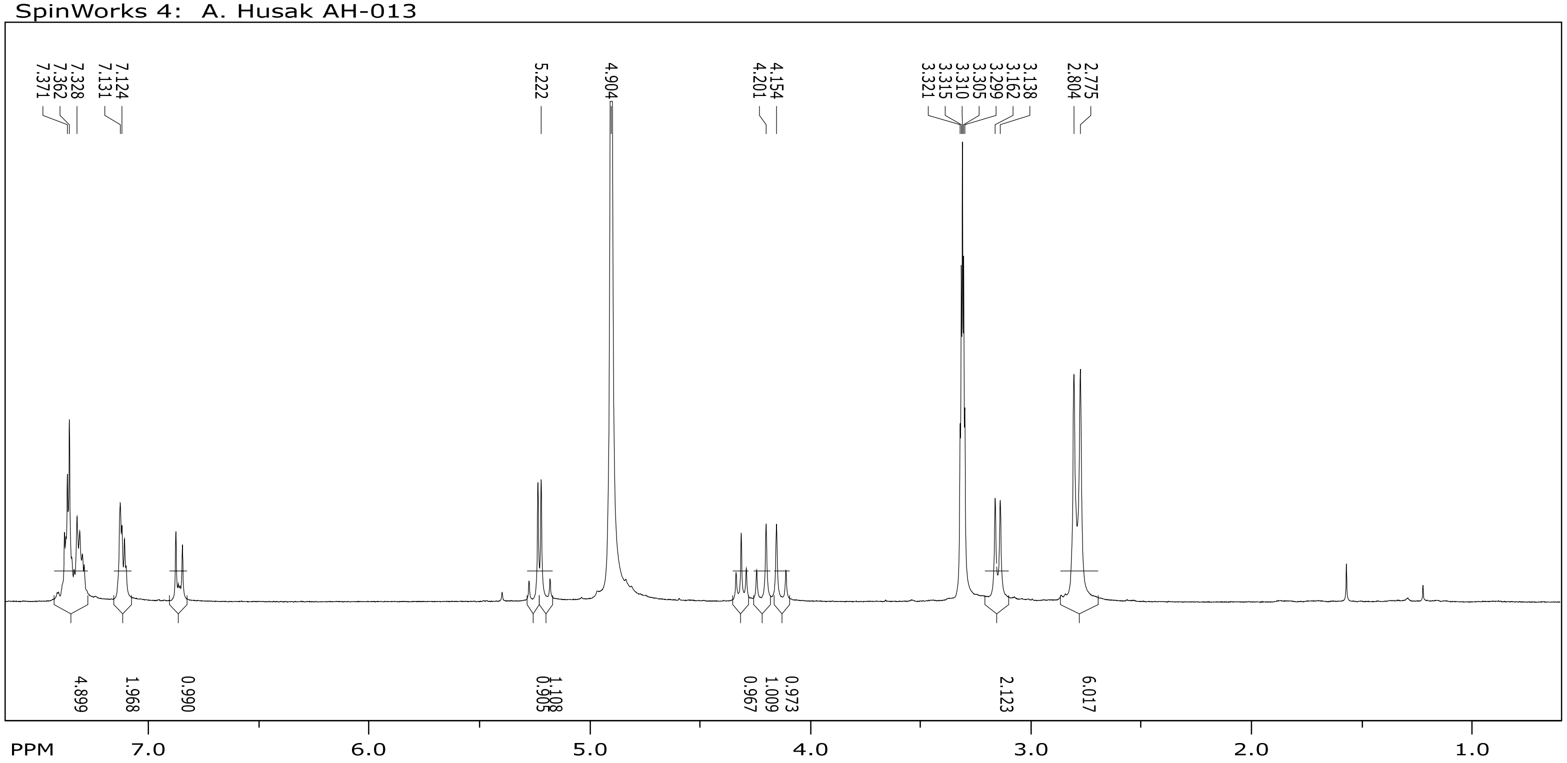


file: G:\Spektri\Rad\AH013\spectrum.dx expt: <zg30>
transmitter freq.: 300.132701 MHz
time domain size: 32768 points
width: 6172.84 Hz = 20.5670 ppm = 0.188380 Hz/pt
number of scans: 0

freq. of 0 ppm: 300.130005 MHz
processed size: 32768 complex points
LB: 0.000 GF: 0.0000

$^{1}$H NMR ($CD_3OD$, 600 MHz) of ***N*-Boc-L-Trp-L-Trp-L-Tyr[$CH_2N(CH_3)_2$]-OBn (3)**

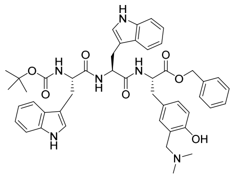


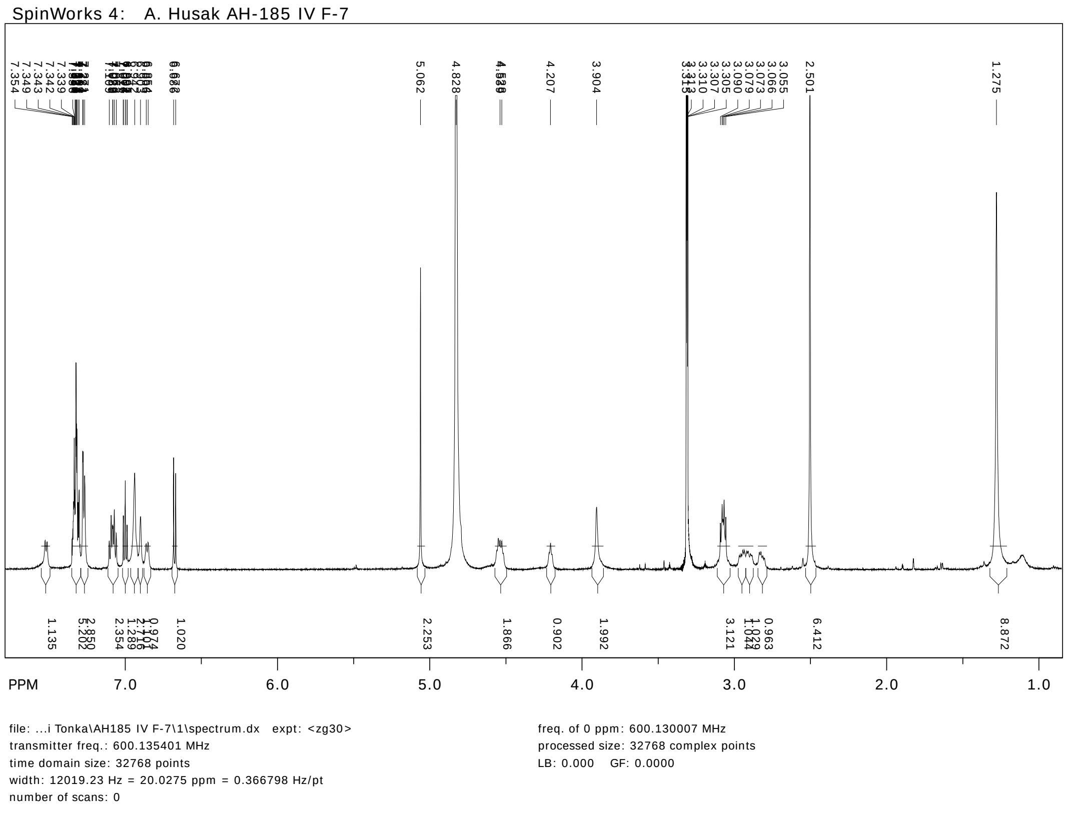

$^{13}$C NMR ($CD_3OD$, 75 MHz, APT) of ***N*-Boc-L-Trp-L-Trp-L-Tyr[CH$_2$N(CH$_3$)$_2$]-OBn (3)**

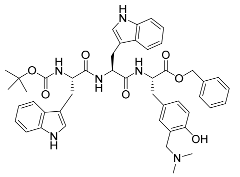


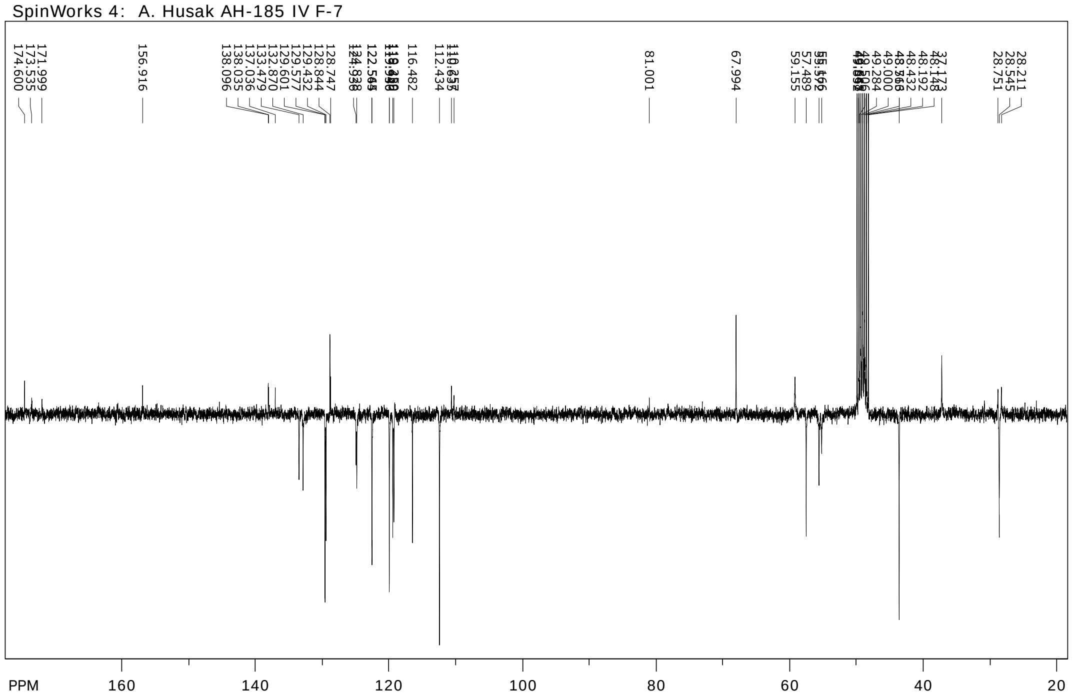


file: ...7\husak_ah185ivffapt\1\spectrum.dx expt: <jmod>
transmitter freq.: 75.475295 MHz
time domain size: 32768 points
width: 17985.61 Hz = 238.2980 ppm = 0.548877 Hz/pt
number of scans: 0

freq. of 0 ppm: 75.467642 MHz
processed size: 32768 complex points
LB: 0.000 GF: 0.0000

$^{1}$H NMR (CD3OD, 600 MHz) of ***N*-Boc-L-Trp-L-Trp-L-Tyr[CH$_2$OCH$_3$]-OBn (3-OMe)**

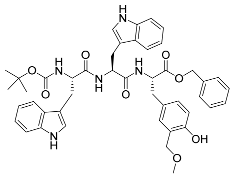


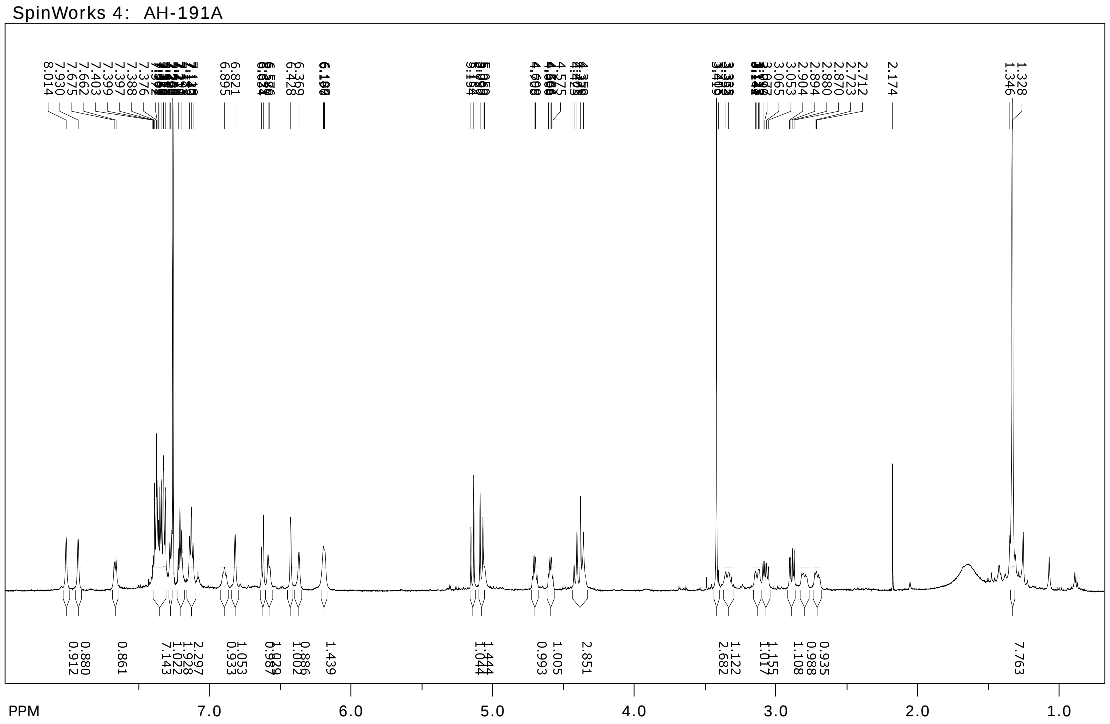


file: ...\Spektri Tonka\AH191 A\spectrum.dx expt: <zg30>
transmitter freq.: 600.135401 MHz
time domain size: 32768 points
width: 12019.23 Hz = 20.0275 ppm = 0.366798 Hz/pt
number of scans: 0

freq. of 0 ppm: 600.130010 MHz
processed size: 32768 complex points
LB: 0.000 GF: 0.0000

$^{13}C$ NMR ($CD_3OD$, 75 MHz, APT) of ***N*-Boc-L-Trp-L-Trp-L-Tyr[CH$_2$OCH$_3$]-OBn (3-OMe)**

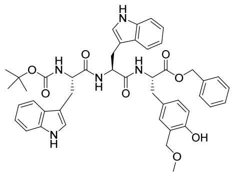

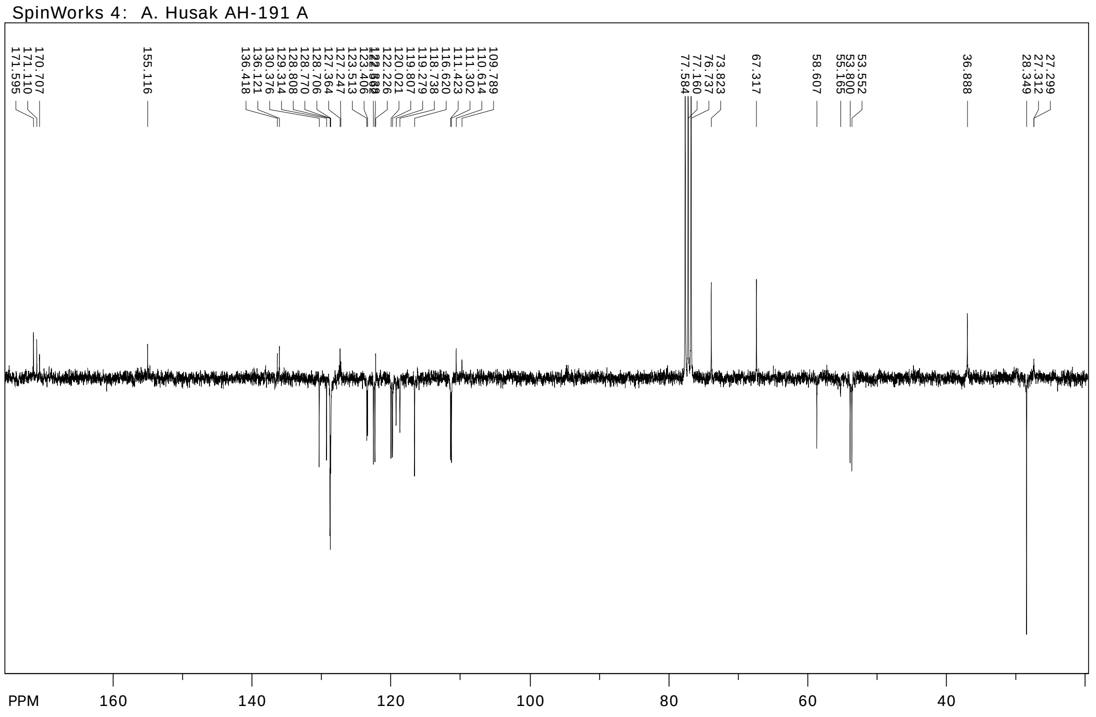


file: ...onka\husak_ah191aapt\1\spectrum.dx expt: <jmod>
transmitter freq.: 75.475295 MHz
time domain size: 32768 points
width: 17985.61 Hz = 238.2980 ppm = 0.548877 Hz/pt
number of scans: 0

freq. of 0 ppm: 75.467737 MHz
processed size: 32768 complex points
LB: 0.000 GF: 0.0000

$^{1}$H NMR ($CD_3OD$, 300 MHz) of **HCl×H-L-Trp-L-Trp-L-Tyr[$CH_2N(CH_3)_2$]-OBn (3×HCl)**

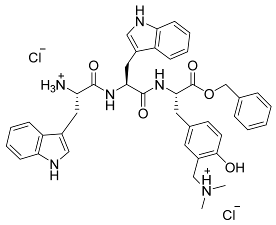

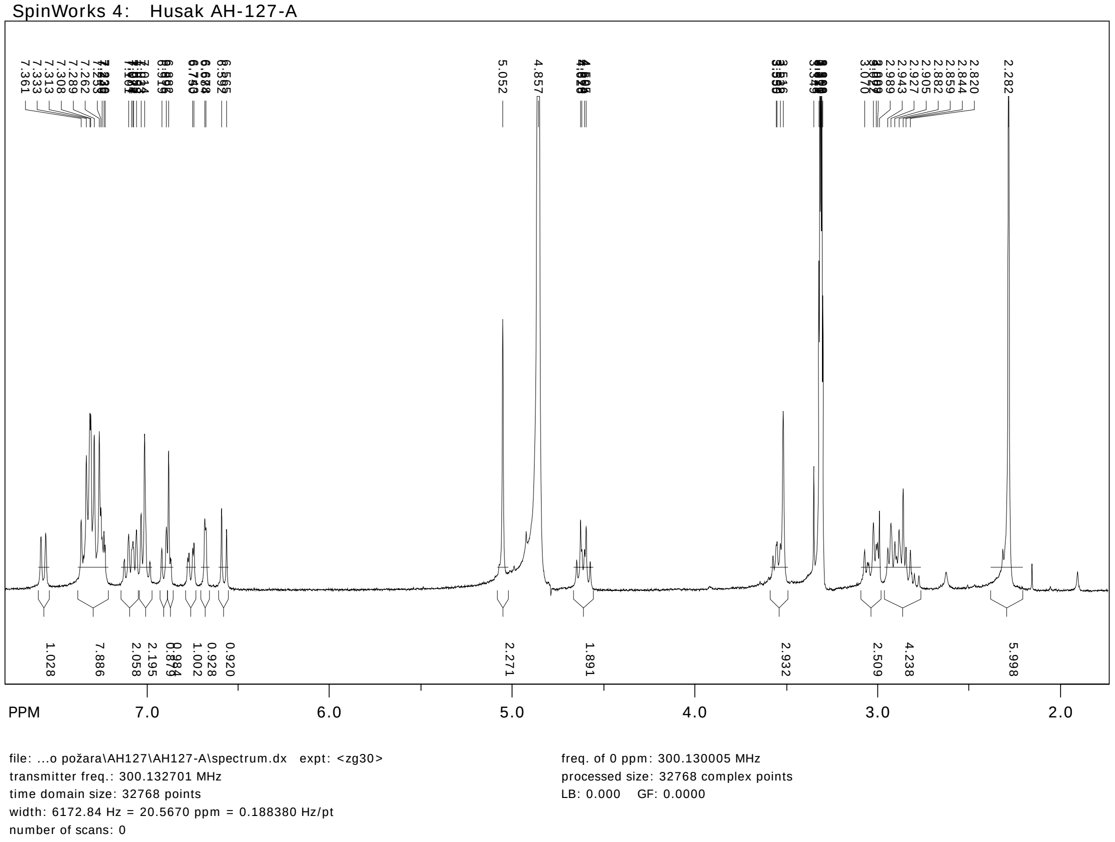

$^{13}$C NMR ($CD_3OD$, 150 MHz) of **HCl×H-L-Trp-L-Trp-L-Tyr[$CH_2N(CH_3)_2$]-OBn (3×HCl)**

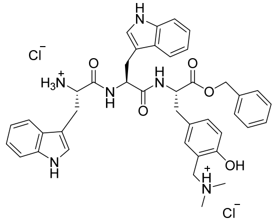

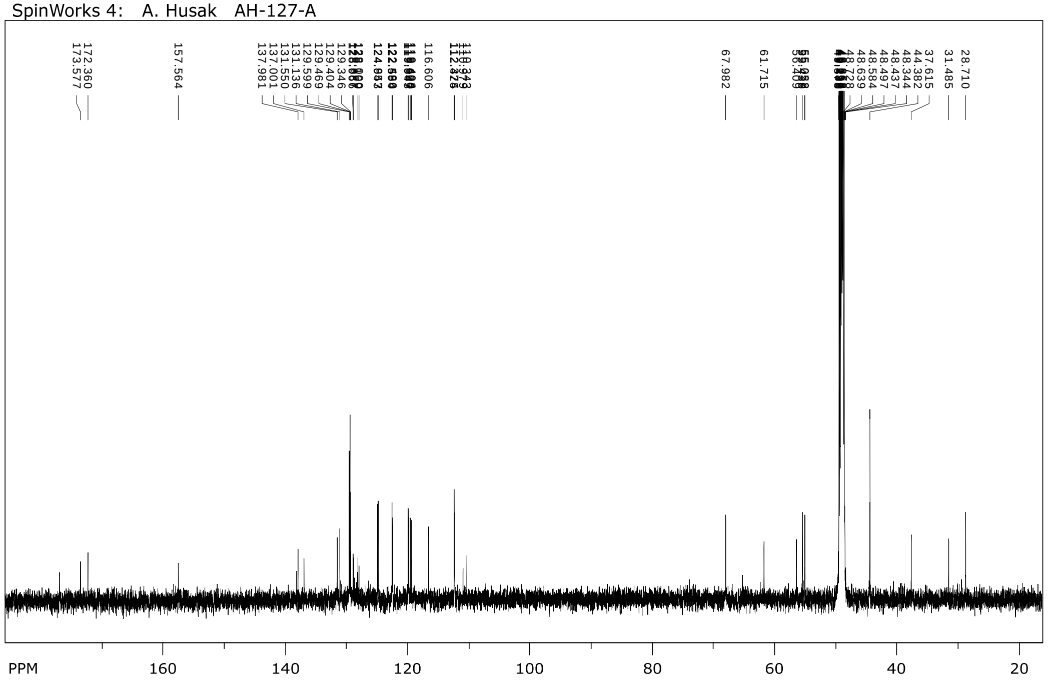


file: ...žara\husak_ah127ac13\2\spectrum.dx expt: <zgpg30>
transmitter freq.: 150.917899 MHz
time domain size: 32768 points
width: 35971.22 Hz = 238.3496 ppm = 1.097755 Hz/pt
number of scans: 0

freq. of 0 ppm: 150.902594 MHz
processed size: 32768 complex points
LB: 0.000 GF: 0.0000

**References**


[1] Husak, A.; Noichl, B. P.; Šumanovac Ramljak, T.; Sohora, M.; Škalamera, Đ.; Budiša, N.; Basarić, N. Photochemical Formation of Quinone Methides from Peptides Containing Modified Tyrosine. *Org. Biomol. Chem*. **2016**, *14*, 10894-10905.

[2] Kumar Mishra, N.; Ballabh Joshi, K.; Verma, S. Inhibitionof Human and Bovine Insulin Fibril Formation by Designed Peptide Conjugates. *Mol. Pharmaceutics* **2013**, *10*, 3903-3912.

[3] Chen, H.; Gao, P.; Zhang, M.; Liao, W.; Zhang, J. Synthesis and Biological Evaluation of a Novel Class of β-carboline Derivatives. *New J. Chem*. **2014**, *38*, 4155-4166.

[4]Molar absorption coefficients and spectra obtained from https://omlc.org/spectra/PhotochemCAD/html/091.html and https://omlc.org/spectra/PhotochemCAD/html/073.html

[5] Molar absorption coefficients and spectra obtained from https://omlc.org/spectra/PhotochemCAD/html/092.html.

[6] Trinquet, E.; Mathis, G. Fluorescence Technologies for the Investigation of Chemical Libraries. *Mol. Bio Syst*. **2006**, *2*, 380-387.
